# Serum piRNA-54265 is a New Biomarker for early detection and clinical surveillance of Human Colorectal Cancer

**DOI:** 10.7150/thno.46241

**Published:** 2020-07-09

**Authors:** Dongmei Mai, Yanfen Zheng, Huan Guo, Peirong Ding, Ruihong Bai, Mei Li, Ying Ye, Jialiang Zhang, Xudong Huang, Dingxin Liu, Qiaoqi Sui, Ling Pan, Jiachun Su, Junge Deng, Guandi Wu, Rui Li, Shuang Deng, Yansen Bai, Yanan Ligu, Wen Tan, Chen Wu, Tangchun Wu, Jian Zheng, Dongxin Lin

**Affiliations:** 1 Sun Yat-sen University Cancer Center, State Key Laboratory of Oncology in South China and Collaborative Innovation Center for Cancer Medicine, Guangzhou, China.; 2 Department of Occupational & Environmental Health, School of Public Health, Tongji Medical College, Huazhong University of Science and Technology, Wuhan, China.; 3 Department of Colorectal Surgery, Sun Yat-sen University Cancer Center, Guangzhou, China.; 4 Department of Pathology, Sun Yat-sen University Cancer Center, Guangzhou, China.; 5 Department of Etiology and Carcinogenesis, National Cancer Center/Cancer Hospital, Chinese Academy of Medical Sciences and Peking Union Medical College, Beijing, China.; 6 Key Laboratory of Environment and Health, Ministry of Education & Ministry of Environmental Protection, State Key Laboratory of Environmental Health (Incubating), School of Public Health, Tongji Medical College, Huazhong University of Science and Technology, Wuhan, Hubei, China.

**Keywords:** colorectal cancer, serum piRNA-54265, biomarker

## Abstract

**Background:** Our previous study has demonstrated an oncogenic role of PIWI-interacting RNA-54265 (piR-54265) in colorectal cancer (CRC). Here, we investigate whether it can be a blood biomarker for population screening and clinical applications.

**Methods:** Serum piR-54265 levels were determined by a digital PCR method in 209 cancer-free healthy controls, 725 patients with CRC, 1303 patients with other types of digestive cancer and 192 patients with benign colorectal tumors. A prospective case-control analysis was conducted to assess the predictive value of serum piR-54265 for future CRC diagnosis. Receiver operating characteristic (ROC) curve was constructed to quantify the diagnostic performance of serum piR-54265 levels by assessing its sensitivity, specificity and respective areas under curve (AUC). The odds ratios (ORs) were computed using multivariate logistic regression models.

**Results:** Serum piR-54265 levels were significantly elevated only in patients with CRC compared with controls and patients with other cancer types. The AUC for recognizing CRC was 0.896 (95% CI, 0.874-0.914), with a sensitivity and specificity being 85.7% and 65.1% at 1500 copies/µL as a cut-off value. The serum piR-54265 levels in patients declined substantially after surgery but increased significantly again when tumor relapses. The prediagnostic serum piR-54265 levels were significantly associated with future CRC diagnosis, with the ORs of 7.23, 2.80, 2.45, and 1.24 for those whose CRC was diagnosed within 1, 2, 3 and >3 years. Serum piR-54265 test is more sensitive than other blood CRC markers.

**Conclusion:** Serum piR-54265 may serve as a valuable biomarker for CRC screening, early detection and clinical surveillance.

## Introduction

Colorectal cancer (CRC) is one of the most common cancers in the developed countries with the morbidity rates rapidly increasing in many developing countries. In 2018, there were approximately 881,000 CRC deaths and more than 1,800,000 new CRC patients diagnosed worldwide 1-3. The prognosis of local advanced CRC (stage III) and CRC with distant metastasis (stage IV) are usually very poor, while early stage CRC (stages I and II) can largely be cured by radical resection 4. Unfortunately, 60-70% of CRC patients with symptom are detected at an advanced stage 5, limiting the curative outcomes of the disease. Detection in earlier stage of the disease or in asymptomatic patients through the use of screening strategies would allow for better outcomes in terms of reducing the disease burden and curing individual patients.

In the last few decades, several strategies have been used worldwide for early screening and detection of CRC that includes (1) stool-based tests such as immunochemical fecal occult blood test (iFOBT), guaiac-based fecal occult blood test (gFOBT) and stool DNA test (e.g., Cologuard®) and (2) visual exams such as colonoscopy, CT colonoscopy and flexible sigmoidoscopy 6-8. Recently, other methods such as plasma *SEPTIN9* gene methylation test have also being developed 9,10. Although several organizations have issued guidelines on individuals or population-based CRC screening and the efforts have contributed more or less to the declined morbidity and mortality of CRC 11-14, the increased annual CRC incidences worldwide highlight the need of developing more effective approaches. On the other hand, currently used screening methods either have uncertain or limited efficiency (e.g., stool-based tests) or are acceptable by few people without symptoms as routine test due to invasiveness, complicate processes and high cost (e.g., colonoscopy) despite its reliable for detection 12,15-17. Thus, discovery and development of more reliable and feasible biomarkers, which enable the identification of individuals at high risk or with early CRC who should further undergo colonoscopy, are in urgent need.

In recent years, circulating non-coding RNAs such as micro RNAs have attracted special attentions as hopeful non-invasive biomarkers for early cancer screening 18-22, although there is still lack of a breakthrough for clinical application. PIWI-interacting RNAs (piRNAs) are a new class of small non-coding RNAs consisting of more than 20,000 members 23-26. Although initially perceived as germline-specific, piRNAs are also expressed in somatic tissues 27. The multifaceted somatic functions and epigenetic regulation of piRNAs have enlightened compelling studies on their roles in human diseases including cancer 28-31. While little is known about their functions, emerging studies have suggested that piRNAs may play an important role in cancer development and may serve as diagnostic and prognostic biomarkers 32-35. Recently, we have identified piRNA-54265 (piR-54265) as an oncogenic RNA and a prognostic biomarker for CRC. More importantly, we found this RNA stably occurs in patient serum and the levels are positively correlated with the level in CRC tissue, suggesting that serum piR-54265 can be the surrogate of CRC piR-54265 36.

Because piR-54265 is associated with CRC and can be readily detected in serum, we proposed that it might be a useful screening biomarker. In this study, we demonstrate that piR-54265 is specifically presented in serum of patients with CRC, but not other digestive cancer types. Serum piR-54265 level decreased dramatically after surgical resection of CRC but increased again when tumor relapsed. We also detected significantly higher serum piR-54265 levels in individuals with high-grade intraepithelial neoplasia compared with controls. A nested case-control analysis in a prospective cohort study showed that prediagnostic serum piR-54265 levels were highly associated with future CRC diagnosis.

## Methods

### Study subjects

For investigating whether serum piR-54265 is a CRC-specific biomarker, we collected blood samples from CRC patients (*N* = 725) and patients with other digestive cancers including esophageal squamous-cell carcinoma (ESCC, *N* = 276), gastric cardia carcinoma (GCC, *N* = 248), gastric carcinoma (GC, *N* = 259), pancreatic ductal adenocarcinoma (PDAC, *N* = 256) or hepatocellular carcinoma (HCC, *N* = 264). Blood samples were also obtained from patients with benign colorectal tumors such as high-grade intraepithelial neoplasia (HIN, *N* = 81) or colorectal polyps (CP, included inflammatory polyps and adenomatous polyps, *N* = 111). In addition, we collected blood samples from 49 CRC patients before and after surgical resection, and blood samples from 3 CRC patients before and after surgery and the time tumor relapse was diagnosed. Patients were diagnosed at Sun Yat-sen University Cancer Center (SYSUCC, Guangzhou) and Chinese Academy of Medical Sciences Cancer Hospital (CAMSCH, Beijing) between 2008 and 2018. Peripheral blood samples (5 mL) from above subjects were collected before any anticancer treatment. All benign and malignant tumors were histopathologically diagnosed and tumor stage was classified according to the 7th edition of AJCC Cancer Staging System. We also collected blood samples from 209 cancer-free individuals defined by physical and colonoscopy examinations as controls. The basic demography characteristics and clinical information of these individuals were obtained from medical records (**Table S1 and S2**).

For investigating whether prediagnostic serum piR-54265 can serve as a screening and predicting biomarker for CRC, we collected blood samples (10 mL) from CRC cases (*N* = 307) and matched controls (*N* = 614) among an ongoing prospective cohort study of Dongfeng-Tongji (DFTJ) in Shiyan city, China, established between September 2008 and June 2010. Detailed information of DFTJ cohort has been published previously 37. All participants were retired employees from Dongfeng Motor Corporation. Each participant was followed up by questionnaires, physical examinations and blood biochemical tests every 5 years. Up to the first follow-up survey in 2013, there were 41,129 participants having the baseline demographical data and medical information. For the survey, the baseline demographic characteristics including age, sex, BMI), family history of cancer and lifestyles of smoking and drinking status and physical activity were collected from all participants. Physical examinations were also done to get their baseline disease status. Each individual donated first morning blood specimen (fasting overnight), which were then stored in -80 °C until laboratory detection. Cases with malignant cancer were found by self-report and confirmed by physician reviews based on medical records and death certificates from the tertiary hospitals in the Corporation Health-Care Service system. International Classification of Diseases 10th Revision was used to classify the diseases and CRC was defined as adenocarcinoma of the colon or rectum (ICD-O-3 code: C18.0-18.9, C19.9, and C20.9). Participants free of malignant diseases, diabetes and cardiovascular diseases at both baseline examination and follow-up survey were selected from the same cohort. They were age (± 1 year and extended to 5 years) and sex frequency-matched to cases at a ratio of 2:1. The demographic and clinical characteristics of cases and controls are shown in**Table S3, S4 and S5**.

Informed consent was obtained from each participant and this study was approved by the Ethical Review Board of Sun Yat-sen University Cancer Center, Chinese Academy of Medical Science Cancer Hospital and Tongji Medical College, Huazhong University of Science and Technology.

### Plasma and serum preparation

Periphery blood sample was collected from individuals fasting overnight. Blood sample was taken into a no addictive vacuum tube or a Vacutainer Plus K_3_EDTA tube (BD Biosciences) and gently stored in room temperature for < 2 h or 4 °C for < 4 h before serum or plasma isolation. To obtain the plasma, blood sample in Vacutainer Plus K_3_EDTA tube was centrifuged at 2,000 ×*g* at 12 °C for 10 min and the supernatant was carefully collected, which was centrifuged again at 2,500 ×*g* for 5 min. To obtain the serum, supernatant in the no additive vacuum tube was carefully collected and centrifuged at 2500 ×*g* at 12 °C for 5 min. Whether the blood sample had hemolysis was evaluated by spectrophotometric measurement of free hemoglobin in a NanoDrop 2000 (Thermo) as described in literature 38,39 and hemolytic sample was excluded for further analysis. The plasma, serum and blood cell samples were stored at -80 °C until use.

### Cell lines and cell culture

Human cell lines used in this study included normal colon epithelial cell line NCM460, CRC cell lines HCT116, LoVo, SW480, SW620, HT-29 and DLD-1, esophageal squamous-cell carcinoma cell lines Kyse30 and Kyse510, gastric carcinoma cell lines HGC27 and SGC7901, pancreatic duct adenocarcinoma cell lines PANC-1 and CFPAC-1 and hepatocarcinoma cell lines Bel-7402, Bel-7404, Hep-G2 and SMMC-7721. NCM460 cells were originated from ATCC cell bank while Kyse30 and Kyse510 cells were generously provided by Dr. Y. Shimada of Hyogo College of Medicine, Japan. The rest cell lines were purchased from the Cell Bank of Type Culture Collection of Chinese Academy of Sciences, Shanghai Institute of Biochemistry. All cell lines passaged for fewer than 6 months and were authenticated by DNA finger printing analysis and were maintained in DMEM medium (Gibco) supplemented with 10% fetal bovine serum (FBS) in an atmosphere of 5% CO_2_ at 37 °C, tested free from mycoplasma infection. Cells (10^5^) were respectively plated in the wells of 12-well plates overnight until adherence and then cultured with 500 μL of DMEM medium free of FBS for 2 h. Cells and corresponding cell-free medium per well were completely and separately collected for piR-54265 analysis.

### Total RNA preparation and reverse transcription

As described previously 36, total RNAs from serum, plasma and blood cells of human blood samples, cell lines and cell culture medium were extracted by using a magnetic beads-based assay (MagMAX™ mirVana™ Total RNA Isolation Kit, Applied Biosystems). An appropriate amount of synthesized *cel*-miR-39 (RuiBiotech) was added as external standard to each sample for estimation of extraction recovery. Reverse transcription reactions were conducted with specific miRNA/piRNA stem-loop primers using the Revert Aid First Strand cDNA Synthesis Kit (Thermo).

### Measurement of piR-54265 levels

The optimally diluted cDNA sample was mixed with the specific primers and probes of piR-54265 and *cel*-miR-39 (**Table S6**) and 2× ddPCR Supermix (Bio-Rad) followed by emulsification (droplet generator, Bio-Rad) and transferring to a 96-well plate. After heat-sealing with foil sheet, the PCR amplification was accomplished in a droplet digital PCR system (C1000 Touch Thermal Cycler, Bio-Rad), which was then transferred to a QX200 droplet reader to determine the positive droplet quantity. Water was included as a negative control in every PCR reaction. Positive and negative events were displayed as dot plots and the piR-54265 level in the reaction was then analyzed by QuantaSoft software (Bio-Rad). The absolute piR-54265 copy numbers were computed based on the known parameters including the level detected by digital PCR, the extraction recovery rate of external standard *cel*-miR-39 and the dilution factor. All measurements were conducted in triplicate and persons who performed the assays were unaware of the sample status.

### Measurement of other clinically used cancer biomarkers

Carcinoembryonic antigen (CEA), carbohydrate antigen 125 (CA125) and carbohydrate antigen 19-9 (CA19-9) levels were measured with chemiluminescence immunoassay (Abbott Architect) in the same serum samples as used for piR-54265. Methylated *SEPTIN9* was analyzed according to the manufacturer's recommendation. Briefly, plasma sample was extracted for tumor DNA using Nucleic Acid Purification/Magnetic Beads Kit (GeneShine Biotechnology) and the resultant DNA was treated with the GS DNA Methylation Kit (GeneShine). The bisulfite-converted DNA was immediately used as the template for *SEPTIN9* methylation analysis with the previous study-reported sequences of primers, blocker and probe for *SEPTIN9* and the internal control* ACTB* 40. PCR analysis was accomplished in an ABI 7500 System (Life Technologies) and the result was determined by collecting the fluorescent signal to obtain the circulating threshold (CT value) and the amplification curve of *ACTB* and *SEPTIN9*. The presence of *SEPTIN9* methylation was defined when one of the three repeat tests was positive. Sample with a CT value > 45 for *SEPTIN9* but a CT value < 38 for *ACTB* was considered as *SEPTIN9* methylation negative.

### Statistical analysis

For normally distributed data, the Student *t*-test was used to assess the significance of differences otherwise the Mann-Whitney U test was used. Quartiles of serum piR-54265 levels were calculated based on the levels in healthy controls. Receiver operating characteristic (ROC) curve was constructed to quantify the diagnostic performance of serum piR-54265 levels by assessing its sensitivity, specificity and respective areas under curve (AUC) with 95% confidence interval (CI) in a binary classifier system. For analyzing the association between prediagnostic serum piR-54265 levels and future diagnosis of CRC in the prospective case-control cohort, the odds ratios (ORs) and 95% CIs were computed using multivariate logistic regression models, with adjustment for BMI, smoking status and alcohol drinking status. We categorized all participants into quartiles and evaluated the ORs by comparing subjects within the forth quartile to those within the first quartile of serum piR-54265 level. We also calculated the ORs per standard deviation (S.D.) change in serum piR-54265 level and for subgroups dichotomized by a set of piR-54265 cut-off values. Analyses were conducted with GraphPad Prism, MedCalc software, R version 3.4.3 and Stata version 12.1 (Stata Corporation). Statistical significance was set at *P* < 0.05 and all were two-sided.

## Results

### Serum piR-54265 is stable and ready for detection and quantification

**Figure 1:**
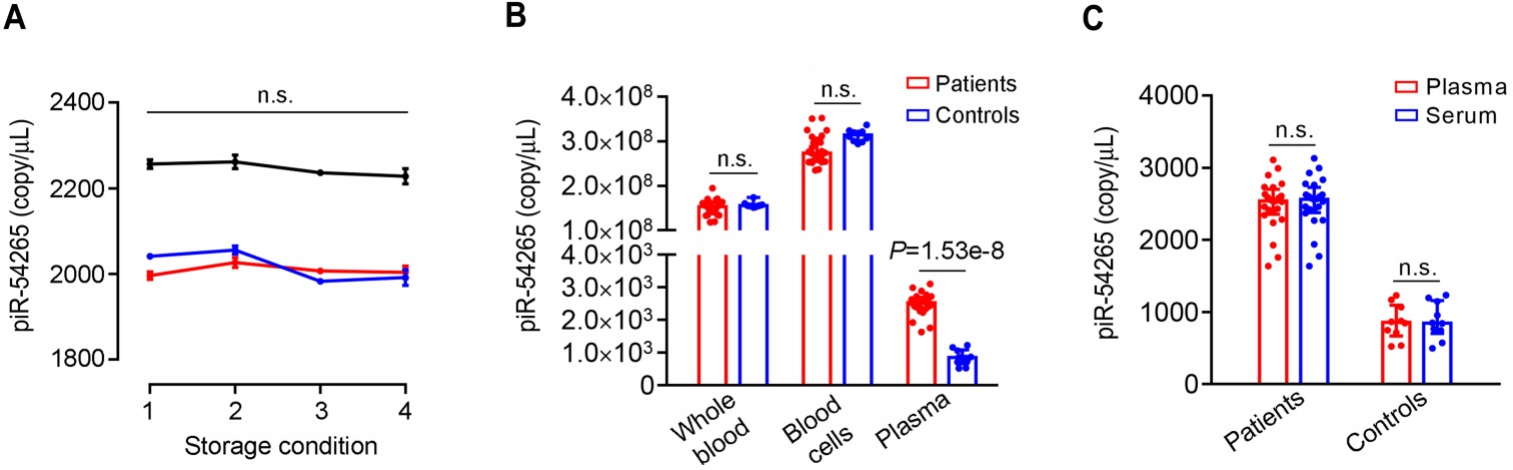
** Serum piR-54265 is stable and feasible in both serum and plasma for detection and quantification. A**, The piR-54265 level in serum of 3 randomly selected individuals with CRC under different storage conditions. Storage conditions were: 1, fresh preparation at room temperature; 2, storage at 37 °C for 24 h; 3, storage at room temperature for 72 h; 4, after 3 times of repeated freeze-and-thaw. Data represent mean ± SEM of 3 measurements of each storage condition; n.s., not significant by one-way ANOVA test.** B**, Different piR-54265 levels in whole blood samples, blood cells and plasma of CRC cases (*N* = 24) and CRC-free controls (*N* = 10). Data represent median with inter-quartile ranges. Mann-Whitney U test shows significant difference only in plasma between CRC patients and controls; n.s., not significant.** C**, Plasma or serum piR-54265 levels (median with inter-quartile range) in CRC patients and controls; n.s., not significant by Mann-Whitney U test.

Our previous study has shown that piR-54265 is fairly stable in the serum and could be ready measured by using real-time quantitative PCR 36. In this study, we established a droplet digital PCR (ddPCR) method to precisely determine the absolute amount of piR-54265 in human serum. By analyzing a large size of serum samples in triplicate, we found that the measurement results were fairly stable and consistent. Repeat measurements of the same serum samples in different analytical batches resulted in an intra-class correlation coefficient (ICC) of 0.99 (**Table S7**). We also assayed serum samples under different storage conditions including fresh extraction for instant detection, incubating at 37 °C for 24 h, staying at room temperature for 72 h and repeated freezing and thawing, and the results showed no significant changes in the piR-54265 copy numbers measured by ddPCR (**Figure 1A**). These results indicate that circulating piR-54265 is stable and can be readily and reliably measured. We then analyzed the piR-54265 levels in different blood components from the same individuals and found that although the piR-54265 levels in plasma of CRC patients were remarkably higher than that in CRC-free controls, the levels in whole blood and blood cells did not differ between patients and controls (**Figure 1B**). Furthermore, we found that the detected piR-54265 levels were identical in either serum or plasma samples (**Figure 1C**), suggesting that both plasma and serum are feasible in the measurement. However, since blood cells contain relatively high level of piR-54265, caution should be taken to avoid hemocytolysis and contamination of blood cells when serum or plasma was prepared.

### Serum piR-54265 is a specific biomarker for CRC and precancerous lesions

**Figure 2:**
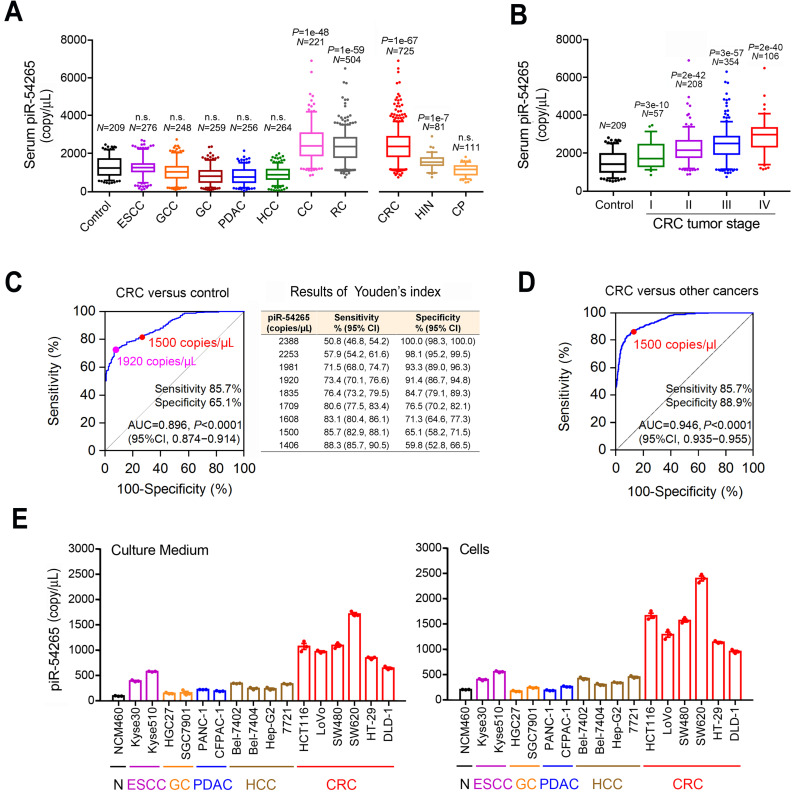
** Serum piR-54265 is an early specific biomarker for CRC. A**, Serum piR-54265 levels (median with inter-quartile range) in cancer-free controls (*N* = 209), patients with colon cancer (CC, *N* = 221), rectum cancer (RC, *N* = 504), high-grade intraepithelial neoplasia (HIN, *N* = 81) and colorectal polyps (CP, *N* = 111), and patients with other types of digestive cancer including ESCC (*N* = 276), GCC (*N* = 248), GC (*N* = 259), PDAC (*N* = 256) and HCC (*N* = 264).* P* for Mann-Whitney U-test; n.s., not significant. **B**, Serum piR-54265 levels (median with inter-quartile range) in cancer-free controls (N = 209), patients with CRC at stage I (*N* = 57), II (*N* = 208), III (*N* = 354) and IV (*N* = 106). *P* value for Mann-Whitney U-test. **C** and** D**, The performance of serum piR-54265 levels in discriminating CRC patients and CRC-free controls and other types of digestive cancer by receiver operating characteristic (ROC) curves analysis. Pink and red dots indicate the example cut-off values estimated by Youden's index analysis. AUC, area under ROC curves. **E**, The piR-54265 levels in both culture media (*left panel*) and cells (*right panel*) of normal intestinal epithelial cell line NCM460 and some other types of digestive cancer cell lines. Results are mean ± SEM from 3 independent cultures and each had 3 replicates. Adherent cells (10^5^) were cultured in 500 µL medium for 2 h before the collection of cells and cell-free culture medium for droplet digital PCR determination of piR-54265 levels. All *P* < 0.0001 of Student's *t*-test for the comparisons of 6 CRC cells with other cell lines.

We have previously shown that patients with CRC had high levels of piR-54265 in their tumors and serum 36. To investigate whether this piRNA is a specific biomarker for CRC, we analyzed serum samples from individuals with different types of digestive cancer including ESCC, GCC, GC, PDAC, HCC and CRC and from cancer-free individuals for comparison. We found that although the median piR-54265 levels (copy/μL serum) in individuals with other types of digestive cancer were similar to that in cancer-free controls (**Figure 2A**), the levels in individuals with CRC was significantly higher than that in controls (2400.3 versus 1213.9; Mann-Whitney U test,* P* = 1.00e-67). Stratifying analysis showed an increasing trend of serum piR-54265 level with advancing CRC stages (Kruskal Wallis test, *P* = 1.70e-15; **Figure 2B**). Serum piR-54265 levels were not associated with sex and age in controls and individuals with different types of digestive cancer (data not shown). These results suggest that piR-54265 is a CRC-specific biomarker. We then evaluated the diagnostic performance of serum piR-54265 in discriminating CRC patients from control subjects. The ROC curve built with 725 CRC patients and 209 controls yielded an area under the curve (AUC) of 0.896 (95% CI, 0.874─0.914, *P* < 0.0001) and Youden's index analysis showed several optimal cut-off values for discriminating CRC patients from healthy controls (**Figure 2C**). For example, 1920 copies/μL had the sensitivity and specificity of 73.4% and 91.4% while 1500 copies/μL had 85.7% and 65.1%, respectively. Further evaluation for the recognition of CRC from other types of digestive cancers also showed a good performance, yielding an AUC of 0.946 (95% CI, 0.935─0.955, *P* < 0.0001; **Figure 2D**). Because serum piR-54265 level at 1500 copies/μL had a sensitivity of 85.7% and a specificity of 88.9%, we considered this level as a reasonable cut-off for the further analyses.

We also compared serum piR-54265 levels among individuals with Stage I CRC, HIN or colorectal polyps (CP) and the results showed that while the median level (copy/mL) in individuals with CP (1183.8) did not significantly differ from that in controls (1213.9), the median levels in individuals with HIN (1572.8, *P* = 1.40e-7) or Stage I CRC (1735.9, *P* = 3.37e-10) was significantly higher than that in controls (**Figure 2A**). The differences in the median levels among individuals with Stage I CRC, HIN and CP were reciprocally significant (Stage I CRC versus HIN, *P* = 0.049; Stage I CRC versus CP, *P* = 4.05e-14 and HIN versus CP, *P* = 5.02e-17). Further evaluation for the identification of HIN, Stage I CRC and Stage II CRC with the cutoff value (1500 copy/μL) also showed good performance (**Figure S1A-D**). All these results indicating that the aberrant overexpression of piR-54265 may be an early event in the development of CRC. Further investigating in cultured normal colon epithelial cells, CRC cells and other types of digestive cancer cells revealed that piR-54265 levels was considerably higher in 6 CRC cell lines and their culture medium than in other types of cancer cell lines and the corresponding culture medium (**Figure 2E**) despite the levels in non-CRC cancer cell lines were slightly higher than that in normal colon epithelial cells. These results were in line with those found in the serum samples from clinical patients and suggest again that piR-54265 is CRC-specific.

### Serum piR-54265 is a biomarker for clinical surveillance of CRC

**Figure 3:**
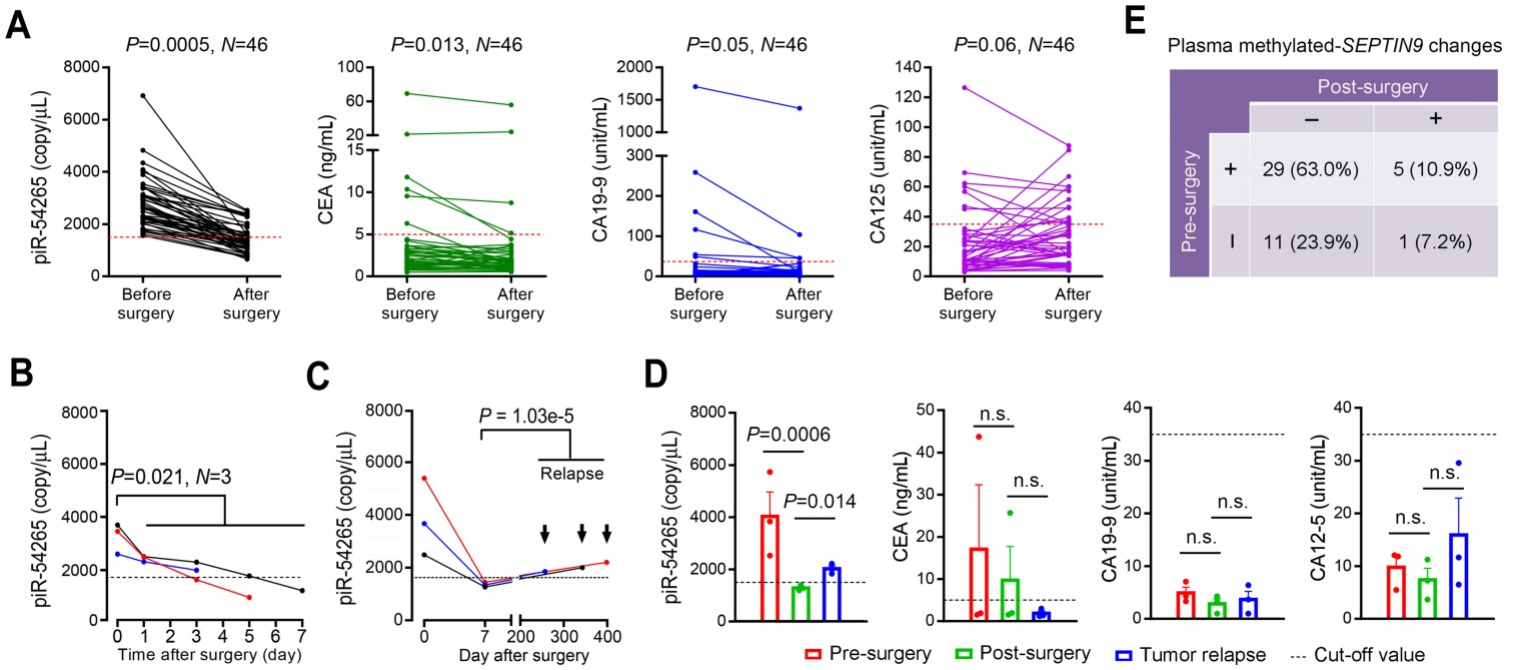
** The effects of serum piR-54265 in CRC clinic surveillance and comparison with other currently used cancer biomarkers. A**, Serum levels of piR-54265, CEA, CA19-9 and CA125 in 46 patients with CRC before and after surgical resection. *P* for Mann-Whitney U-test. **B**, Continual decline of serum piR-54265 levels in 3 patients after different days of surgical removal of CRC. *P* of Student's *t*-test for the average levels before and after surgery. **C**, Serum levels of piR-54265, CEA, CA19-9 and CA125 in 3 CRC patients before and after surgery and the time tumor relapse was diagnosed. *P* for Student's *t*-test; n.s., not significant. **D**, Serum piR-54265 levels in 3 CRC patients before surgery, 7 days after surgery, and at the time when tumor relapses as indicated by the arrows. *P* for Student's *t*-test. **E**, Plasma methylated-*SEPTIN9* changes in 46 patients before and after surgical removal of CRC. The dot lines in **A**-**D** indicate the cut-off values of each marker (piR-54265, 1500 copies/µL; CEA, 5 ng/mL; CA19-9, 35 unit/mL and CA125, 35 unit/mL).

Since piR-54265 is overexpressed in CRC tumor tissues and also presents at a high level in serum of individuals with the disease, we proposed that this circulating piRNA might serve as a specific biomarker for clinical surveillance of CRC. To test this, we analyzed the serum piR-54265 level in CRC patients (*N* = 46) before and 3-7 days after curative tumor resection. The results showed that all patients had their serum piR-54265 levels higher than the cut-off value (1500 copies/μL) before tumor resection; however, the levels in all patients were significantly declined after CRC was removed (*P* = 0.005; **Figure 3A**) and a time-dependent decline after surgery was observed in 3 patients whose blood samples were collected on different days after tumor resection (**Figure 3B**). These results prompted us to examine whether the serum piR-54265 level rises again when CRC relapses. By analyzing 3 CRC patients whose blood samples were collected before surgery, 7 days after surgery and the time when CRC relapse was diagnosed, we found that although the serum piR-54265 level in each patient was dramatically dropped to a level less than the cut-off value, it was significantly elevated again to a diagnostic level (**Figure 3C-D**). All these results suggest that the elevated serum piR-54265 in CRC patients derive from CRC and thus may serve as a new effective indicator for CRC relapse.

We also examined the other clinically used serum or plasma biomarkers including CEA, CA19-9, CA125, and methylated *SEPTIN9* for comparison. The results showed that among this set of 46 patients, only 6 (13.0%), 6 (13.0%) and 7 (15.2%) had their serum CEA, CA19-9 and CA125 levels higher than the cut-off value, respectively. Furthermore, not all of these 3 markers in all patients declined after CRC removed; in contrary, some patients had increased CA125 level after CRC resection (**Figure 3A**). In 3 patients with CRC relapse, we did not see any significant changes of serum CEA, CA19-9 and CA125 levels (**Figure 3C**), contrasting with piR-54265. For methylated *SEPTIN9*, we detected 73.9% (34/46) of patients that were positive before surgery and 85.3% (29/34) of these positive patients became negative after surgery (**Figure 3E**). We also compared these five markers among another set of 101 CRC patients. Consistent with the above results, serum piR-54265 performed well with the best accuracy of 85.1% (86/101) at its cut-off value while methylated *SEPTIN9* was just 61.4% and CEA, CA125 and CA19-9 showed almost no effectiveness (**Table S8**).

### Prediagnostic serum piR-54265 levels are associated with future CRC diagnosis

We next tested in the prospective cohort study whether serum piR-54265 has the ability to predict future CRC. Analysis of a nested case-control panel consisting of 307 individuals with CRC and 614 matched controls derived from the prospective cohort study (**Table S5**) showed that the median serum piR-54265 level (copy/μL) in individuals with CRC diagnosed at entire time period of follow-up (9 years) was significantly higher than that in controls (1453.3 versus 1208.0,* P* = 2.13e-12), which was associated with 2.10-fold (95% CI, 1.66-2.65) increased risk of future diagnosis of CRC when the forth quartile was compared to the first quartile (reference) for the serum piR-54265 level. Stratification analysis revealed that the association was significant in individuals with blood samples collected within 3 years before diagnosis, with the OR being the highest in those whose CRC was diagnosed within 1 year after blood samples collected (OR, 7.23; 95% CI, 4.00-13.08), followed by 2 years (OR, 2.80; 95% CI, 1.60-4.89), 3 years (OR, 2.45; 95% CI, 1.49-4.03) and > 3 years (OR, 1.24; 95% CI, 0.90-1.72) (**Table 1**). We then examined the performance of serum piR-54265 for CRC prediction using different cut-off values and found that the value of ≥ 1500 copies/μL had a preferable sensitivity and specificity in predicting future diagnosis of CRC within 3 years after blood sample collection, with the ORs of 3.97 (95% CI, 2.42-6.50), 2.49 (95% CI, 1.37-4.50), 3.13 (95% CI, 1.82-5.38) and 1.66 (95% CI, 1.07-2.57) for 1, 2, 3 and > 3 years, respectively (**Table 2**).

We also compared the performance of serum piR-54265 with the 3 serum protein biomarkers in predicting CRC in the prospective case-control analysis. As shown in **Table S9**, serum CEA, CA125, and CA19-9 had little ability in discriminating CRC. For example, for patients diagnosed within 1 year after blood taken, the AUCs built with CEA (0.592), CA125 (0.459) and CA19-9 (0.429) were similar to each other, but significantly lower than that built with serum piR-54265 (0.743). Combination of the three protein markers with piR-54265 did not significantly increased the AUCs as compared with piR-54265 alone. We further compared the five markers in 143 CRC cases from the prospective cohort who had enough plasma samples for simultaneous determination of methylated *SEPTIN9* and the results showed that serum piR-54265 performed much better than methylated *SEPTIN9* to distinguish the future CRC (**Table S10**).

## Discussion

We have recently reported that piR-54265 is an oncogenic RNA in the development of human CRC, functioning via PIWIL2/STAT3 signaling pathway 36. In the current study, we have achieved several new results. Firstly, by analyzing serum samples from patients suffering from 6 different types of digestive cancer, we have demonstrated that serum piR-54265 levels are significantly elevated only in CRC patients and the higher levels are already presented in individuals with CRC precancerous lesions (e. g., HIN) as compared with CRC-free controls. Secondly, we have demonstrated that after surgical removal of CRC tumors, the serum piR-54265 levels substantially decline while the levels significantly increase again when the tumor relapses, suggesting that the elevated serum piR-54265 is derived from CRC tumor cells. Thirdly, we have found in a prospective case-control analysis that serum piR-54265 is able to warn CRC 3 years before diagnosis. Together, these findings verify serum piR-54265 a specific CRC-related molecule and a valuable biomarker for population screening, early diagnosis and clinical surveillance of CRC.

We have previously shown that piR-54265 is overexpressed in CRC tissues and can also be readily and reliably measured by using qRT-PCR method in serum as the surrogate of piR-54265 level in tumor tissue 36. In the present study, we have developed a ddPCR method to precisely quantify the absolute copy number of this piRNA in serum. On the basis of analyzing a large size of serum samples in triplicate, we found the measurement results considerably stable and consistent, no matter in different detection batches or under various conditions such as fresh extraction for instant detection, room temperature or 37 °C incubation for days and multigelation, indicating both the stability of this circulating piRNA and the reliability of the ddPCR detection method. Therefore, the ddPCR test of serum piR-54265 has the advantages of accuracy, relative convenience, non-invasion and speediness for CRC screening and surveillance. However, caution should be taken in preparation of serum or plasma samples because of the existence of abundant piR-54265 in blood cells; hemolysis and (or) blood cell contamination must be avoided. In this study, we observed an unprovoked high serum piR-54265 level in a few CRC-free controls, which could be due to an accidental contamination of blood cell piR-54265 when preparation of serum samples. If this could be the case, our results might have underestimated the ability of serum piR-54265 in CRC recognition in some extent.

To comprehensively evaluate the application prospect of serum piR-54265 as a novel and valuable CRC-specific biomarker, we have compared its ability in CRC detection or screening with some other serum tumor markers routinely used in clinic, including CEA, CA19-9 and CA125, and found that serum piR-54265 is much more sensitive than the other 3 markers. Our results also demonstrate that serum piR-54265 performs much better than plasma methylated *SEPTIN9*, a test requires at least 5 mL of blood and complicated procedures to enrich the rare methylation forms and finally gives only a qualitative judgment of negative or positive 9,10. However, serum piR-54265 ddPCR detection is far more easily performed requiring only 0.1 mL of serum and finally provides a quantitative copy number, which is important in monitoring the disease status. Apart from blood-based test, several fecal occult blood tests including gFOBT, iFOBT and FIT, and fecal DNA tests such as Cologuard®, are available for clinical CRC detection; however, sampling for these tests is relatively inconvenient and the specificity or sensitivity has been considered unsatisfactory 6-8. Colonoscopy, the gold standard for CRC diagnosis when combined with pathological examinations, is currently recommended for CRC screening and early detection in high-risk individuals. However, the examination compliance in asymptomatic individuals is poor probably due to a high cost, the inconvenient preparation processes and invasive procedures 12. Furthermore, colonoscopy is unable to define high-risk population and to predict further CRC and thus has limited application values in population screening and clinic CRC surveillance. Serum piR-54265 detection may play a navigation role to discover high-risk individuals for precise early diagnosis and treatment or to monitor CRC relapse status in patients after treatment.

Our study also has some limitations. Although we have compared several digestive cancer types and found serum piR-54265 is CRC-specific, further exclusion of this RNA in other cancer types, especially adenocarcinoma with enteric differentiation in lung, cervix and endometrium, would be significant. Due to the difficulty in recruiting large number of patients who can donate blood samples before surgery, several days after surgery, and at the time when tumor recurrent which usually take several years or even decades, the sample size for analyzing clinical surveillance of CRC is small and further validation is needed. We found that serum piRNA-54265 is increased in patients at the time of relapse, but the possibility that this trend will also be seen in relapse-free patients cannot be excluded. Since currently we have no such samples for analysis, ongoing perspective studies would be helpful to address this issue. Furthermore, because of low compliance, most of control subjects in the nest case-control analysis from the DFTJ prospective cohort were not diagnosed by colonoscopy. Thus, we cannot exclude the possibility that a few controls suffered from early CRC or HIN and had elevated serum piR-54265 levels. Nevertheless, if so, this would just lead to an underestimate of prediction ability of piR-54265. In addition, since this study was conducted in Chinese population with only one prospective cohort, further studies in other ethnic populations and multiple prospective cohorts would be warranted to validate our findings.

In conclusion, by analyzing clinical cancer patients and a prospective cohort, we have demonstrated serum piR-54265 a new CRC-specific molecule. This circulating tumor RNA is readily and reliably determined in serum samples and may serve as a valuable noninvasive biomarker for population screening, early detection and clinical surveillance of CRC. Since serum piR-54265 test exhibits many advantages over the conventional cancer markers and colonoscopy in terms of sensitivity/specificity, expediency, rapidity and invasiveness, it would have broad application prospects.

## Supplementary Material

Supplementary figure and tables.

## Figures and Tables

**Table 1:** The prediagnostic serum piR-54265 levels (copy number per μL) were associated with future CRC diagnosis

	*N*	Median level (IQR)^a^	*P*^b^	OR (95% CI)^c^	OR (95% CI) per S.D.^d^
Controls	614	1208.0 (662.3, 1514.0)	Reference	Reference	Reference
Cases	307	1453.3 (1108.4, 2086.9)	2.13e-12	2.10 (1.66, 2.65)	1.69 (1.43, 1.99)
Diagnosed at 0 year	79	1635.7 (1230.9, 2629.0)	1.77e-12	7.23 (4.00, 13.08)	3.98 (2.63, 6.02)
Diagnosed at 1 year	53	1509.7 (1129.1, 2439.9)	7.66e-05	2.80 (1.60, 4.89)	2.06 (1.39, 3.06)
Diagnosed at 2 years	66	1489.3 (1114.6, 1900.5)	2.24e-04	2.45 (1.49, 4.03)	1.86 (1.32, 2.62)
Diagnosed at 3 years	109	1309.5 (748.2, 1715.6)	4.62e-02	1.24 (0.90, 1.72)	1.16 (0.93, 1.45)

a: IQR, inter-quartile range. b: Mann-Whitney U test. c: Comparison of the forth quartile to the first quartile (Reference) for the serum piR-54265 level. Data were computed with logistic regression models and adjusted for BMI, smoking and alcohol drinking status. d: Per S.D. increased in the serum piR-54265 level.

**Table 2:** The odds ratios (OR), sensitivity and specificity for discrimination of CRC diagnosed in different follow-up time with different cut-off values of serum piR-54265 levels

Diagnosed within follow-up time	piR-54265 (copy/μL)	Cases/controls	OR^a^ (95% CI)	Sensitivity (95% CI)	Specificity (95% CI)
0 year	< 1800	47/552	5.60 (3.27, 9.60)	40.51 (30.37, 51.53)	89.90 (87.27, 92.04)
	≥ 1800	32/62			
	< 1700	42/529	5.21 (3.12, 8.69)	46.84 (36.24, 57.73)	85.99 (83.02, 88.52)
	≥ 1700	37/85			
	< 1600	36/496	4.84 (2.94, 7.96)	54.43 (43.50, 64.95)	80.78 (77.48, 83.70)
	≥ 1600	43/118			
	< 1500	32/453	3.97 (2.42, 6.50)	59.49 (48.47, 69.63)	73.94 (70.33, 77.26)
	≥ 1500	47/161			
	< 1400	25/413	4.25 (2.55, 7.08)	68.35 (57.45, 77.55)	67.26 (63.45, 70.86)
	≥ 1400	54/201			
1 year	< 1800	31/552	5.32 (2.79, 10.17)	41.51 (29.26, 54.91)	89.90 (87.27, 92.04)
	≥ 1800	22/62			
	< 1700	31/529	3.66 (1.95, 6.88)	41.51 (29.26, 54.91)	85.83 (82.85, 88.37)
	≥ 1700	22/85			
	< 1600	30/496	2.71 (1.47, 5.00)	43.40 (30.95, 56.73)	80.78 (77.48, 83.70)
	≥ 1600	23/118			
	< 1500	26/453	2.49 (1.37, 4.50)	50.94 (37.88, 63.88)	73.78 (70.16, 77.10)
	≥ 1500	27/161			
	< 1400	24/413	2.19 (1.21, 3.94)	54.72 (41.45, 67.34)	67.26 (63.45, 70.86)
	≥ 1400	29/201			
2 years	< 1800	47/552	4.06 (2.18, 7.54)	28.79 (19.27, 40.64)	89.90 (87.27, 92.04)
	≥ 1800	19/62			
	< 1700	45/529	3.32 (1.84, 6.00)	31.82 (21.85, 43.79)	85.99 (83.02, 88.52)
	≥ 1700	21/85			
	< 1600	42/496	2.78 (1.59, 4.89)	36.36 (25.81, 48.42)	80.78 (77.48, 83.70)
	≥ 1600	24/118			
	< 1500	34/453	3.13 (1.82, 5.38)	48.48 (36.85, 60.29)	73.94 (70.33, 77.26)
	≥ 1500	32/161			
	< 1400	30/413	2.82 (1.64, 4.82)	54.55 (42.62, 65.98)	67.26 (63.45, 70.86)
	≥ 1400	36/201			
3 years	< 1800	87/552	2.35 (1.35, 4.09)	21.10 (14.49, 29.68)	89.90 (87.27, 92.04)
	≥ 1800	22/62			
	< 1700	79/529	2.33 (1.42, 3.82)	27.52 (20.01, 36.56)	85.99 (83.02, 88.52)
	≥ 1700	30/85			
	< 1600	76/496	1.83 (1.14, 2.92)	30.28 (22.44, 39.45)	80.78 (77.48, 83.70)
	≥ 1600	33/118			
	< 1500	69/453	1.66 (1.07, 2.57)	36.70 (28.25, 46.05)	73.94 (70.33, 77.26)
	≥ 1500	40/161			
	< 1400	61/413	1.63 (1.06, 2.49)	44.04 (35.08, 53.40)	67.26 (63.45, 70.86)
	≥ 1400	48/201			

a: Computed with logistic regression models, adjusted for BMI, smoking and alcohol drinking status and family history of cancer. CI, confidence interval.

## Abbreviations

AUC: area under receiver-operating characteristic curve
BMI: body mass index
CC: colon cancer
CI: confidence intervals
CRC: colorectal cancer
ddPCR: droplet digital polymerase chain reaction
ESCC: esophageal squamous cell carcinoma
GC: gastric carcinoma
GCC: gastric cardia carcinoma
HCC: hepatocellular carcinoma
HIN: high-grade intraepithelial neoplasia
ICC: intra-class correlation coefficient
OR: odds ratio
PDAC: pancreatic ductal adenocarcinoma
piRNA: PIWI-interacting RNA
RC: rectal cancer
ROC curve: receiver-operating characteristic curve
