# Supplementary material for: Serum piRNA-54265 is a New Biomarker for early detection and clinical surveillance of Human Colorectal Cancer
Source: Theranostics. 2020 Jul 9;10(19):8468–78. doi: 10.7150/thno.46241 (PMC7392023; doi:10.7150/thno.46241)
Supplement: Supplementary file 1 — Supplementary figure and tables. [file thnov10p8468s1.pdf]

Mai et al. Fig. S1

A

HIN versus controls

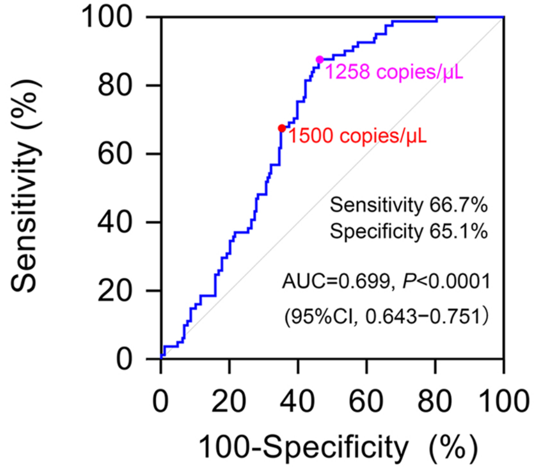

Results of Youden's index

piR-54265 (copies/μl)	Sensitivity % (95%CI)	Specificity % (95%CI)
1800	21.0 (12.7, 31.5)	84.2 (78.5, 88.9)
1698	37.0 (26.6, 48.5)	75.6 (69.2, 81.3)
1601	48.2 (36.9, 59.5)	70.8 (64.1, 76.9)
1501	66.7 (55.3, 76.8)	65.1 (58.2, 71.5)
1401	75.3 (64.5, 84.2)	58.9 (51.9, 65.6)
1301	85.2 (75.6, 92.1)	54.1 (47.1, 61.0)
1258*	87.7 (78.5, 93.9)	54.1 (47.1, 61.0)
1205	88.9 (80.0, 94.8)	49.8 (42.8, 56.7)
1095	92.6 (84.6, 97.2)	42.6 (35.8, 49.6)
983	97.5 (91.4, 99.7)	34.5 (28.0, 41.3)

B

Stage I CRC versus controls

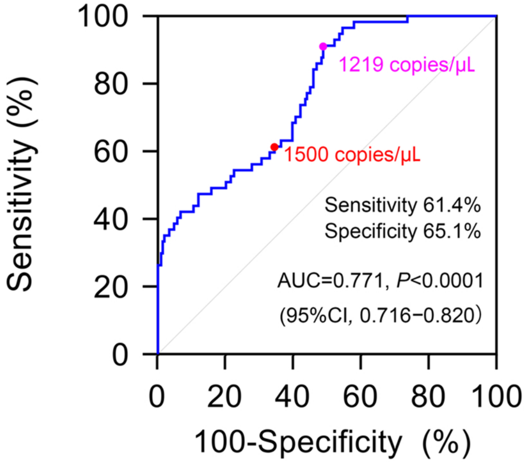

Results of Youden's index

piR-54265 (copies/μl)	Sensitivity % (95%CI)	Specificity % (95%CI)
1981	42.1 (29.1, 55.9)	93.3 (89.0, 96.3)
1901	42.1 (29.1, 55.9)	89.5 (85.1, 93.7)
1787	49.1 (35.6, 62.7)	84.2 (78.5, 88.9)
1709	54.4 (40.7, 67.6)	76.1 (69.7, 81.7)
1601	56.1 (42.4, 69.3)	70.8 (64.1, 76.9)
1501	61.4 (47.6, 74.0)	65.1 (58.2, 71.5)
1401	70.2 (56.6, 81.6)	58.9 (51.9, 65.6)
1304	79.0 (66.1, 88.6)	54.6 (47.5, 61.4)
1219*	91.2 (80.7, 97.1)	51.2 (44.2, 58.2)
1106	96.5 (87.9, 99.6)	43.1 (36.3, 50.1)

C

Stage II CRC versus controls

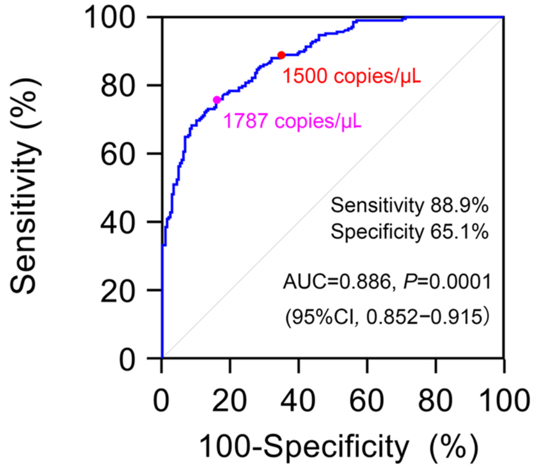

Results of Youden's index

piR-54265 (copies/μl)	Sensitivity % (95%CI)	Specificity % (95%CI)
1988	64.4 (57.5, 70.9)	93.3 (89.0, 96.3)
1901	69.7 (63.0, 75.9)	90.0 (85.1, 93.7)
1787*	76.0 (69.6, 81.6)	84.2 (78.5, 88.9)
1700	80.3 (74.2, 85.5)	75.6 (69.2, 81.3)
1601	85.6 (80.1, 90.1)	70.8 (64.1, 76.9)
1501	88.9 (83.9, 92.9)	65.1 (58.2, 71.5)
1401	89.9 (85.0, 93.6)	58.9 (51.9, 65.6)
1304	93.3 (89.0, 96.3)	54.6 (47.5, 61.4)
1204	95.2 (91.3, 97.7)	49.3 (42.3, 56.3)
1106	99.1 (96.6, 99.9)	43.1 (36.3, 50.1)

D

Stage I/II CRC versus controls

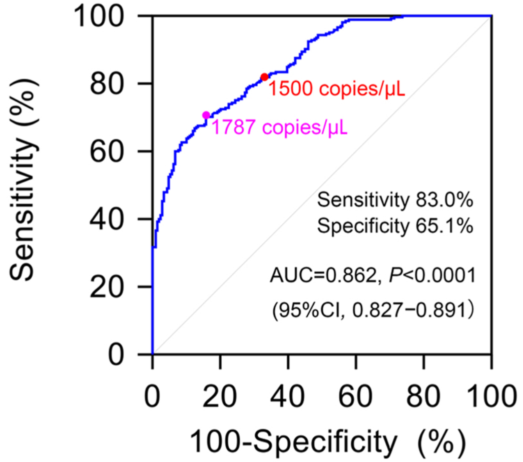

Results of Youden's index

piR-54265 (copies/μl)	Sensitivity % (95%CI)	Specificity % (95%CI)
1981	60.0 (53.8, 65.9)	93.3 (89.0, 96.3)
1900	63.8 (57.7, 69.6)	89.5 (84.5, 93.3)
1787*	70.2 (64.3, 75.6)	84.2 (78.5, 88.9)
1700	74.7 (69.0, 79.8)	75.6 (69.2, 81.3)
1601	79.3 (73.9, 84.0)	70.8 (64.1, 76.9)
1501	83.0 (77.9, 87.3)	65.1 (58.2, 71.5)
1401	85.7 (80.9, 89.6)	58.9 (51.9, 65.6)
1304	90.2 (86.0, 93.5)	54.6 (47.5, 61.4)
1205	94.3 (90.8, 96.8)	49.8 (42.8, 56.7)
1106	98.5 (96.2, 99.6)	43.1 (36.3, 50.1)

Supplementary Figure Legends

Figure S1. Serum piR-54265 is an early specific marker for CRC. A−D, The performance of serum piR-54265 levels in discriminating HIN (**A**) or stage I (**B**) or stage II (**C**) or stage I and II (**D**) of CRC patients and CRC-free controls by receiver operating characteristic (ROC) curves analysis. Pink and red dots indicate the example cut-off values estimated by Youden's index analysis. AUC, area under ROC curves.

Supplementary tables

Table of Contents

Table S1. Clinical characteristics and serum piR-54265 level of CRC-free controls and patients with different cancer in this study..........4

Table S2. Demographic and clinical characteristics and serum piR-54265 level of patients with CRC..........43

Table S3. General characteristics of participants in the nested case-control analysis derived from the Dongfeng-Tongji prospective cohort..........60

Table S4. The accumulated numbers of incident CRC cases diagnosed by years in the prospective Dongfeng-Tongji cohort..........61

Table S5. Demographic characteristics, clinical features and serum piR-54265 level of the participants in prospective case-control analysis from Dongfeng-Tongji cohort..........62

Table S6. Primers and probes used for droplet digital PCR in this study..........96

Table S7. The piR-54265 levels of 30 serum specimens in two detection batches (copy/μL)..........97

Table S8. Comparison of serum piR-54265 with other blood markers for the efficacy of recognizing patients with colorectal cancer..........98

Table S9. The ability of prediagnostic serum piR-54265, CEA, CA125 and CA19-9 alone or combination for predicting future CRC..........99

Table S10. Comparison of prediagnostic serum piR-54265 with other blood markers for the efficacy of recognizing future CRC patients..........100

Table S1. Clinical characteristics and serum piR-54265 level of CRC-free controls and patients with different cancer in this study

Sample_ID	Subject[1]	Center[2]	Sex	Age (year)	Serum piR-54265[3]	
					copy/μL	SEM
Ctrl1	Control	SYSUCC	Male	57	2063.6	10.2
Ctrl2	Control	SYSUCC	Female	56	620.9	20.5
Ctrl3	Control	SYSUCC	Male	54	1513.4	18.1
Ctrl4	Control	SYSUCC	Female	26	1608.3	2.9
Ctrl5	Control	SYSUCC	Female	40	1480.1	29.0
Ctrl6	Control	SYSUCC	Female	39	1835.5	12.0
Ctrl7	Control	SYSUCC	Male	36	1900.9	18.0
Ctrl8	Control	SYSUCC	Male	44	2090.2	20.5
Ctrl9	Control	SYSUCC	Female	50	1218.8	12.3
Ctrl10	Control	SYSUCC	Female	48	1035.1	21.8
Ctrl11	Control	SYSUCC	Female	49	1477.8	17.0
Ctrl12	Control	SYSUCC	Female	39	1765.7	17.6
Ctrl13	Control	SYSUCC	Male	41	771.3	24.4
Ctrl14	Control	SYSUCC	Male	56	2313.1	9.2
Ctrl15	Control	SYSUCC	Female	44	696.1	37.9
Ctrl16	Control	SYSUCC	Female	37	1056	13.6
Ctrl17	Control	SYSUCC	Male	41	1019.9	21.9
Ctrl18	Control	SYSUCC	Female	45	1204.7	15.8
Ctrl19	Control	SYSUCC	Male	53	2385.5	22.0
Ctrl20	Control	SYSUCC	Female	55	1257.3	41.7
Ctrl21	Control	SYSUCC	Male	43	782.5	33.5
Ctrl22	Control	SYSUCC	Female	35	1500.6	19.3
Ctrl23	Control	SYSUCC	Female	54	921.9	13.0
Ctrl24	Control	SYSUCC	Male	39	809.3	22.9
Ctrl25	Control	SYSUCC	Female	53	1734	37.2
Ctrl26	Control	SYSUCC	Male	42	1468	22.3
Ctrl27	Control	SYSUCC	Male	45	875.8	25.6
Ctrl28	Control	SYSUCC	Female	46	782.4	35.7
Ctrl29	Control	SYSUCC	Female	42	691.3	11.3
Ctrl30	Control	SYSUCC	Male	52	1895.1	39.8
Ctrl31	Control	SYSUCC	Female	34	1964.7	21.7
Ctrl32	Control	SYSUCC	Female	65	1910.7	19.8
Ctrl33	Control	SYSUCC	Female	36	904.9	27.0
Ctrl34	Control	SYSUCC	Male	30	982.7	27.0
Ctrl35	Control	SYSUCC	Male	52	1588.5	23.1
Ctrl36	Control	SYSUCC	Male	71	1148.1	26.4
Ctrl37	Control	SYSUCC	Female	43	1844.5	13.4
Ctrl38	Control	SYSUCC	Male	56	1215.6	12.8

Ctrl39	Control	SYSUCC	Female	28	1023.1	26.8
Ctrl40	Control	SYSUCC	Female	52	604.7	36.1
Ctrl41	Control	SYSUCC	Male	43	940.6	3.4
Ctrl42	Control	SYSUCC	Female	53	1945.3	23.8
Ctrl43	Control	SYSUCC	Male	40	2234.2	23.6
Ctrl44	Control	SYSUCC	Male	44	1008.2	17.4
Ctrl45	Control	SYSUCC	Male	59	1710.9	29.4
Ctrl46	Control	SYSUCC	Male	38	783.8	33.6
Ctrl47	Control	SYSUCC	Male	32	784.8	15.4
Ctrl48	Control	SYSUCC	Female	47	1648.2	31.7
Ctrl49	Control	SYSUCC	Male	42	1073.5	11.7
Ctrl50	Control	SYSUCC	Female	53	2132.6	11.4
Ctrl51	Control	SYSUCC	Male	51	1181.6	23.6
Ctrl52	Control	SYSUCC	Male	54	1237.4	20.4
Ctrl53	Control	SYSUCC	Female	48	1166	25.3
Ctrl54	Control	SYSUCC	Female	58	1340.7	9.2
Ctrl55	Control	SYSUCC	Female	30	1662.8	36.2
Ctrl56	Control	SYSUCC	Male	45	764	7.4
Ctrl57	Control	SYSUCC	Female	52	1708.5	21.7
Ctrl58	Control	SYSUCC	Female	37	1565.1	28.1
Ctrl59	Control	SYSUCC	Male	46	1590.1	16.2
Ctrl60	Control	SYSUCC	Male	37	639.5	11.6
Ctrl61	Control	SYSUCC	Female	51	1839.9	0.1
Ctrl62	Control	SYSUCC	Male	59	2043.7	38.3
Ctrl63	Control	SYSUCC	Male	37	1712.4	8.6
Ctrl64	Control	SYSUCC	Female	47	1332.2	29.7
Ctrl65	Control	SYSUCC	Female	52	1839.1	44.4
Ctrl66	Control	SYSUCC	Male	40	779.8	25.2
Ctrl67	Control	SYSUCC	Female	36	898.8	37.4
Ctrl68	Control	SYSUCC	Female	48	1471.9	20.0
Ctrl69	Control	SYSUCC	Female	51	1981.2	21.0
Ctrl70	Control	SYSUCC	Female	50	1709.1	19.9
Ctrl71	Control	SYSUCC	Male	46	1224.3	21.2
Ctrl72	Control	SYSUCC	Male	32	1663.5	20.5
Ctrl73	Control	SYSUCC	Female	39	1616.3	33.9
Ctrl74	Control	SYSUCC	Male	51	1465.1	18.9
Ctrl75	Control	SYSUCC	Male	52	2198.4	28.3
Ctrl76	Control	SYSUCC	Male	67	1179.2	41.6
Ctrl77	Control	SYSUCC	Male	49	1477.1	21.9
Ctrl78	Control	SYSUCC	Female	43	1213.9	13.2
Ctrl79	Control	SYSUCC	Male	63	795.7	24.2
Ctrl80	Control	SYSUCC	Male	51	579.4	41.1
Ctrl81	Control	SYSUCC	Female	47	706.2	5.3
Ctrl82	Control	SYSUCC	Female	48	1051.4	22.1

Ctrl83	Control	SYSUCC	Female	54	1698.1	11.1
Ctrl84	Control	SYSUCC	Male	55	2164.6	32.2
Ctrl85	Control	SYSUCC	Female	66	841.6	2.9
Ctrl86	Control	SYSUCC	Male	36	1095.3	44.3
Ctrl87	Control	SYSUCC	Female	30	1537.6	35.1
Ctrl88	Control	SYSUCC	Male	42	1527.8	36.3
Ctrl89	Control	SYSUCC	Male	54	613.8	21.6
Ctrl90	Control	SYSUCC	Female	60	1695.5	22.1
Ctrl91	Control	SYSUCC	Male	52	2253.6	0.1
Ctrl92	Control	SYSUCC	Male	45	2261.5	27.0
Ctrl93	Control	SYSUCC	Female	51	1406.2	13.0
Ctrl94	Control	SYSUCC	Female	45	1068.1	25.7
Ctrl95	Control	SYSUCC	Female	48	1303.7	2.8
Ctrl96	Control	SYSUCC	Female	53	1017.8	20.5
Ctrl97	Control	SYSUCC	Female	54	1306.9	34.0
Ctrl98	Control	SYSUCC	Female	60	1194.2	20.9
Ctrl99	Control	SYSUCC	Female	55	2154.6	19.1
Ctrl100	Control	SYSUCC	Female	47	1561.6	27.3
Ctrl101	Control	SYSUCC	Female	44	1721.9	32.1
Ctrl102	Control	SYSUCC	Male	45	1763.8	30.2
Ctrl103	Control	SYSUCC	Male	40	1415.8	22.2
Ctrl104	Control	SYSUCC	Male	37	1884.5	12.6
Ctrl105	Control	SYSUCC	Male	34	811.8	46.5
Ctrl106	Control	SYSUCC	Female	50	2387.8	35.6
Ctrl107	Control	SYSUCC	Male	32	2072.5	25.3
Ctrl108	Control	SYSUCC	Male	44	1635.7	32.4
Ctrl109	Control	SYSUCC	Female	49	883.5	11.9
Ctrl110	Control	SYSUCC	Female	36	1785.4	41.4
Ctrl111	Control	SYSUCC	Male	47	1343	8.7
Ctrl112	Control	SYSUCC	Female	50	951.6	18.1
Ctrl113	Control	SYSUCC	Female	50	729.8	8.4
Ctrl114	Control	SYSUCC	Female	51	1781	6.6
Ctrl115	Control	SYSUCC	Female	55	1850.9	8.8
Ctrl116	Control	SYSUCC	Male	50	1113.4	31.8
Ctrl117	Control	SYSUCC	Male	51	1873.6	25.5
Ctrl118	Control	SYSUCC	Male	54	642.4	21.8
Ctrl119	Control	SYSUCC	Female	28	1054.5	37.8
Ctrl120	Control	SYSUCC	Male	56	1837.6	19.4
Ctrl121	Control	SYSUCC	Male	60	1978.3	29.6
Ctrl122	Control	SYSUCC	Female	61	862.6	34.4
Ctrl123	Control	SYSUCC	Male	52	612	38.3
Ctrl124	Control	SYSUCC	Female	42	1725.1	30.2
Ctrl125	Control	SYSUCC	Female	63	1861.3	21.4
Ctrl126	Control	SYSUCC	Female	51	1755.8	18.9

Ctrl127	Control	SYSUCC	Female	41	509.1	10.3
Ctrl128	Control	SYSUCC	Female	29	506.7	28.5
Ctrl129	Control	SYSUCC	Female	28	878.27	79.6
Ctrl130	Control	SYSUCC	Female	26	906.36	47.5
Ctrl131	Control	SYSUCC	Male	30	585.04	38.6
Ctrl132	Control	SYSUCC	Female	35	485.1	16.9
Ctrl133	Control	SYSUCC	Female	26	484.84	46.4
Ctrl134	Control	SYSUCC	Male	26	721.4	105
Ctrl135	Control	SYSUCC	Male	32	458.46	47.3
Ctrl136	Control	SYSUCC	Male	31	665.83	30.8
Ctrl137	Control	SYSUCC	Female	28	1203.5	68.8
Ctrl138	Control	SYSUCC	Female	27	825	18.4
Ctrl139	Control	SYSUCC	Female	26	720.6	30.7
Ctrl140	Control	SYSUCC	Male	26	876.88	55.6
Ctrl141	Control	SYSUCC	Male	26	679.81	12.0
Ctrl142	Control	SYSUCC	Male	28	631.54	77.9
Ctrl143	Control	SYSUCC	Female	28	923.3	36.6
Ctrl144	Control	SYSUCC	Female	26	692.18	55.0
Ctrl145	Control	SYSUCC	Male	28	1601.24	23.8
Ctrl146	Control	SYSUCC	Male	33	1181.8	24.5
Ctrl147	Control	SYSUCC	Male	31	1012.3	15.8
Ctrl148	Control	SYSUCC	Male	31	1534.61	29.7
Ctrl149	Control	SYSUCC	Female	26	562.4	60.7
Ctrl150	Control	SYSUCC	Male	33	688.38	82.2
Ctrl151	Control	SYSUCC	Male	25	1320.8	28.4
Ctrl152	Control	SYSUCC	Female	22	1530.8	6.4
Ctrl153	Control	SYSUCC	Female	32	955.3	32.2
Ctrl154	Control	SYSUCC	Female	23	976.5	13.9
Ctrl155	Control	SYSUCC	Male	41	1063.1	29.7
Ctrl156	Control	SYSUCC	Male	30	663.6	25.5
Ctrl157	Control	SYSUCC	Female	46	1679	21.9
Ctrl158	Control	SYSUCC	Female	46	1105.8	11.2
Ctrl159	Control	SYSUCC	Female	41	735.1	23.8
Ctrl160	Control	SYSUCC	Female	48	1734.9	18.2
Ctrl161	Control	SYSUCC	Male	26	1237.6	18.1
Ctrl162	Control	SYSUCC	Male	41	976.7	17.7
Ctrl163	Control	SYSUCC	Female	24	852.7	26.9
Ctrl164	Control	SYSUCC	Female	50	1920	32.4
Ctrl165	Control	SYSUCC	Male	45	1228.9	2.5
Ctrl166	Control	SYSUCC	Female	45	1758.1	27.3
Ctrl167	Control	SYSUCC	Male	45	1258.2	13.3
Ctrl168	Control	SYSUCC	Female	38	749.3	21.2
Ctrl169	Control	SYSUCC	Male	48	1883	29.8
Ctrl170	Control	SYSUCC	Female	46	1680.9	28.2

Ctrl171	Control	SYSUCC	Female	36	808.8	14.8
Ctrl172	Control	SYSUCC	Male	36	1527.4	21.5
Ctrl173	Control	SYSUCC	Female	26	1191	21.3
Ctrl174	Control	SYSUCC	Male	23	1915.4	31.4
Ctrl175	Control	SYSUCC	Male	29	1386.1	18.7
Ctrl176	Control	SYSUCC	Female	41	720.5	9.7
Ctrl177	Control	SYSUCC	Female	37	1165.7	16.8
Ctrl178	Control	SYSUCC	Male	30	565.5	13.5
Ctrl179	Control	SYSUCC	Female	22	1787.4	9.8
Ctrl180	Control	SYSUCC	Female	41	1779.3	13.5
Ctrl181	Control	SYSUCC	Female	38	817.3	26.1
Ctrl182	Control	SYSUCC	Male	42	1750.4	29.7
Ctrl183	Control	SYSUCC	Male	27	1460.4	42.5
Ctrl184	Control	SYSUCC	Female	39	1345	11.7
Ctrl185	Control	SYSUCC	Female	30	1073.6	36.4
Ctrl186	Control	SYSUCC	Male	31	932.5	72.7
Ctrl187	Control	SYSUCC	Male	46	1315.4	100.8
Ctrl188	Control	SYSUCC	Female	28	1009.8	43.3
Ctrl189	Control	SYSUCC	Female	46	1493.1	29.6
Ctrl190	Control	SYSUCC	Male	32	713.8	72.5
Ctrl191	Control	SYSUCC	Female	38	1404.6	3.7
Ctrl192	Control	SYSUCC	Female	44	1047.8	39.1
Ctrl193	Control	SYSUCC	Female	26	1473.4	93.4
Ctrl194	Control	SYSUCC	Male	25	1552.9	24.9
Ctrl195	Control	SYSUCC	Male	36	1727.2	50.6
Ctrl196	Control	SYSUCC	Female	42	1401	33.3
Ctrl197	Control	SYSUCC	Female	20	1148	15.2
Ctrl198	Control	SYSUCC	Female	44	760.5	29.3
Ctrl199	Control	SYSUCC	Female	35	952.3	53.9
Ctrl200	Control	SYSUCC	Male	44	1109.1	24.2
Ctrl201	Control	SYSUCC	Male	47	1141.2	57.2
Ctrl202	Control	SYSUCC	Male	34	1041.6	57.7
Ctrl203	Control	SYSUCC	Female	34	748.9	22.8
Ctrl204	Control	SYSUCC	Female	48	778.5	32.7
Ctrl205	Control	SYSUCC	Male	31	780.8	61.3
Ctrl206	Control	SYSUCC	Female	26	861.5	20.9
Ctrl207	Control	SYSUCC	Male	27	545.2	87.7
Ctrl208	Control	SYSUCC	Female	21	545.6	44.8
Ctrl209	Control	SYSUCC	Female	44	569.9	30.6
ESCC1	ESCC	CHCAMS	Male	57	2454.0	2.0
ESCC2	ESCC	CHCAMS	Male	47	2516.0	2.3
ESCC3	ESCC	CHCAMS	Male	26	1306.0	1.4
ESCC4	ESCC	CHCAMS	Male	48	1064.0	1.2
ESCC5	ESCC	CHCAMS	Female	64	1462.0	2.1

ESCC6	ESCC	CHCAMS	Female	71	1323.0	2.2
ESCC7	ESCC	CHCAMS	Female	63	1440.0	2.5
ESCC8	ESCC	CHCAMS	Male	38	1496.0	2.7
ESCC9	ESCC	CHCAMS	Male	30	1280.0	2.4
ESCC10	ESCC	CHCAMS	Female	26	1052.0	2.0
ESCC11	ESCC	CHCAMS	Female	62	1340.0	2.6
ESCC12	ESCC	CHCAMS	Male	45	1603.0	3.2
ESCC13	ESCC	CHCAMS	Male	74	1666.0	3.5
ESCC14	ESCC	CHCAMS	Male	56	1444.0	3.1
ESCC15	ESCC	CHCAMS	Female	51	1607.0	3.8
ESCC16	ESCC	CHCAMS	Male	63	1372.0	3.4
ESCC17	ESCC	CHCAMS	Female	44	1036.0	2.5
ESCC18	ESCC	CHCAMS	Female	70	1453.0	3.6
ESCC19	ESCC	CHCAMS	Male	45	2332.0	5.8
ESCC20	ESCC	CHCAMS	Female	42	1607.0	4.0
ESCC21	ESCC	CHCAMS	Female	51	1857.0	4.7
ESCC22	ESCC	CHCAMS	Female	62	953.0	2.4
ESCC23	ESCC	CHCAMS	Male	67	1335.0	3.4
ESCC24	ESCC	CHCAMS	Male	65	1857.0	4.8
ESCC25	ESCC	CHCAMS	Male	74	1367.1	3.8
ESCC26	ESCC	CHCAMS	Male	70	1230.0	3.6
ESCC27	ESCC	CHCAMS	Female	53	1455.0	4.2
ESCC28	ESCC	CHCAMS	Female	65	1765.0	5.4
ESCC29	ESCC	CHCAMS	Female	58	1455.0	4.6
ESCC30	ESCC	CHCAMS	Male	49	2688.0	8.5
ESCC31	ESCC	CHCAMS	Female	61	1425.0	4.6
ESCC32	ESCC	CHCAMS	Male	30	1198.0	4.0
ESCC33	ESCC	CHCAMS	Female	67	1302.9	4.3
ESCC34	ESCC	CHCAMS	Male	27	1201.0	4.0
ESCC35	ESCC	CHCAMS	Male	45	2031.0	6.9
ESCC36	ESCC	CHCAMS	Male	73	575.0	2.0
ESCC37	ESCC	CHCAMS	Male	45	1113.9	3.9
ESCC38	ESCC	CHCAMS	Female	50	1607.0	5.7
ESCC39	ESCC	CHCAMS	Female	37	1160.0	4.2
ESCC40	ESCC	CHCAMS	Male	65	1603.0	5.8
ESCC41	ESCC	CHCAMS	Male	66	2316.0	8.7
ESCC42	ESCC	CHCAMS	Male	46	560.0	2.1
ESCC43	ESCC	CHCAMS	Female	57	1207.0	4.6
ESCC44	ESCC	CHCAMS	Male	56	1215.0	4.7
ESCC45	ESCC	CHCAMS	Male	56	1179.0	4.6
ESCC46	ESCC	CHCAMS	Female	32	1564.0	6.2
ESCC47	ESCC	CHCAMS	Female	66	1556.0	6.2
ESCC48	ESCC	CHCAMS	Female	69	1791.0	7.2
ESCC49	ESCC	CHCAMS	Male	58	2077.1	8.4

ESCC50	ESCC	CHCAMS	Female	67	1455.0	6.1
ESCC51	ESCC	CHCAMS	Male	65	1209.0	5.1
ESCC52	ESCC	CHCAMS	Female	51	2114.0	9.0
ESCC53	ESCC	CHCAMS	Male	52	1527.0	6.6
ESCC54	ESCC	CHCAMS	Female	35	1350.0	5.9
ESCC55	ESCC	CHCAMS	Male	62	1370.1	6.0
ESCC56	ESCC	CHCAMS	Male	54	1578.0	7.0
ESCC57	ESCC	CHCAMS	Female	32	1094.0	4.9
ESCC58	ESCC	CHCAMS	Female	46	1396.0	6.3
ESCC59	ESCC	CHCAMS	Female	54	1501.1	6.9
ESCC60	ESCC	CHCAMS	Female	71	1427.0	6.7
ESCC61	ESCC	CHCAMS	Male	32	1324.0	6.4
ESCC62	ESCC	CHCAMS	Female	50	1547.0	7.5
ESCC63	ESCC	CHCAMS	Male	51	1282.0	6.3
ESCC64	ESCC	CHCAMS	Male	65	1475.0	7.2
ESCC65	ESCC	CHCAMS	Female	46	1570.0	7.7
ESCC66	ESCC	CHCAMS	Male	28	1309.0	6.4
ESCC67	ESCC	CHCAMS	Female	38	1461.0	7.3
ESCC68	ESCC	CHCAMS	Male	41	1112.0	5.6
ESCC69	ESCC	CHCAMS	Female	75	1319.0	6.6
ESCC70	ESCC	CHCAMS	Female	43	1409.0	7.3
ESCC71	ESCC	CHCAMS	Male	46	958.0	5.0
ESCC72	ESCC	CHCAMS	Female	72	1275.0	6.8
ESCC73	ESCC	CHCAMS	Male	42	1472.0	7.8
ESCC74	ESCC	CHCAMS	Female	60	1492.0	8.0
ESCC75	ESCC	CHCAMS	Female	74	1215.9	6.6
ESCC76	ESCC	CHCAMS	Female	75	1278.0	7.0
ESCC77	ESCC	CHCAMS	Female	50	2495.1	13.7
ESCC78	ESCC	CHCAMS	Female	34	1085.0	6.0
ESCC79	ESCC	CHCAMS	Female	73	1285.0	7.1
ESCC80	ESCC	CHCAMS	Male	56	1248.0	7.0
ESCC81	ESCC	CHCAMS	Female	74	1338.0	7.5
ESCC82	ESCC	CHCAMS	Male	66	1294.0	7.3
ESCC83	ESCC	CHCAMS	Male	27	1584.0	8.9
ESCC84	ESCC	CHCAMS	Male	63	1435.0	8.1
ESCC85	ESCC	CHCAMS	Female	49	1121.0	6.4
ESCC86	ESCC	CHCAMS	Male	41	2439.0	14.0
ESCC87	ESCC	CHCAMS	Female	48	2241.0	13.0
ESCC88	ESCC	CHCAMS	Male	75	1737.1	10.1
ESCC89	ESCC	CHCAMS	Female	59	1237.0	7.2
ESCC90	ESCC	CHCAMS	Male	65	2316.0	13.7
ESCC91	ESCC	CHCAMS	Male	53	1694.0	10.1
ESCC92	ESCC	CHCAMS	Male	34	1369.0	8.1
ESCC93	ESCC	CHCAMS	Male	70	936.0	5.6

ESCC94	ESCC	CHCAMS	Male	56	1408.0	8.5
ESCC95	ESCC	CHCAMS	Male	32	1589.0	9.6
ESCC96	ESCC	CHCAMS	Male	42	1475.9	9.0
ESCC97	ESCC	CHCAMS	Female	55	1482.0	9.0
ESCC98	ESCC	CHCAMS	Male	49	1246.0	7.7
ESCC99	ESCC	CHCAMS	Male	27	1489.0	9.3
ESCC100	ESCC	CHCAMS	Male	35	2396.0	15.0
ESCC101	ESCC	CHCAMS	Female	41	1239.0	7.8
ESCC102	ESCC	CHCAMS	Male	27	1322.1	8.4
ESCC103	ESCC	CHCAMS	Female	38	2063.1	13.1
ESCC104	ESCC	CHCAMS	Male	73	1561.0	10.2
ESCC105	ESCC	CHCAMS	Male	73	926.9	6.2
ESCC106	ESCC	CHCAMS	Female	44	1410.0	9.4
ESCC107	ESCC	CHCAMS	Female	66	1451.0	9.7
ESCC108	ESCC	CHCAMS	Female	67	1327.0	8.9
ESCC109	ESCC	CHCAMS	Female	29	2230.0	15.0
ESCC110	ESCC	CHCAMS	Female	25	1123.0	7.6
ESCC111	ESCC	CHCAMS	Female	62	1114.0	7.6
ESCC112	ESCC	CHCAMS	Female	63	1601.0	10.9
ESCC113	ESCC	CHCAMS	Male	55	1181.0	8.0
ESCC114	ESCC	CHCAMS	Male	59	2729.0	18.7
ESCC115	ESCC	CHCAMS	Female	49	1601.0	11.0
ESCC116	ESCC	CHCAMS	Male	59	1313.0	9.0
ESCC117	ESCC	CHCAMS	Male	70	1731.0	11.9
ESCC118	ESCC	CHCAMS	Female	75	1236.0	8.5
ESCC119	ESCC	CHCAMS	Female	32	923.9	6.4
ESCC120	ESCC	CHCAMS	Male	56	1081.0	7.6
ESCC121	ESCC	CHCAMS	Male	31	1264.0	8.8
ESCC122	ESCC	CHCAMS	Female	50	1474.0	10.4
ESCC123	ESCC	CHCAMS	Female	71	1156.0	8.2
ESCC124	ESCC	CHCAMS	Male	29	1459.0	10.4
ESCC125	ESCC	CHCAMS	Female	28	1168.0	8.3
ESCC126	ESCC	CHCAMS	Male	73	1424.0	10.3
ESCC127	ESCC	CHCAMS	Female	47	1312.1	9.5
ESCC128	ESCC	CHCAMS	Female	56	2431.0	17.7
ESCC129	ESCC	CHCAMS	Male	56	451.0	3.3
ESCC130	ESCC	CHCAMS	Female	54	1486.9	11.0
ESCC131	ESCC	CHCAMS	Female	58	914.0	6.9
ESCC132	ESCC	CHCAMS	Male	63	1433.0	11.1
ESCC133	ESCC	CHCAMS	Female	71	1314.0	10.2
ESCC134	ESCC	CHCAMS	Male	61	2188.1	17.0
ESCC135	ESCC	CHCAMS	Male	36	1299.0	10.2
ESCC136	ESCC	CHCAMS	Female	50	1984.9	15.6
ESCC137	ESCC	CHCAMS	Female	49	2071.0	16.6

ESCC138	ESCC	CHCAMS	Female	40	1428.0	11.4
ESCC139	ESCC	CHCAMS	Female	68	1314.1	10.5
ESCC140	ESCC	CHCAMS	Male	38	1128.0	9.1
ESCC141	ESCC	CHCAMS	Female	70	947.0	7.7
ESCC142	ESCC	CHCAMS	Male	28	1177.0	9.7
ESCC143	ESCC	CHCAMS	Female	38	1398.0	11.7
ESCC144	ESCC	CHCAMS	Female	42	1338.0	11.3
ESCC145	ESCC	CHCAMS	Female	74	1181.0	10.1
ESCC146	ESCC	CHCAMS	Female	60	1656.0	14.2
ESCC147	ESCC	CHCAMS	Male	48	1485.0	12.7
ESCC148	ESCC	CHCAMS	Male	54	819.0	7.1
ESCC149	ESCC	CHCAMS	Male	34	1087.0	9.4
ESCC150	ESCC	CHCAMS	Male	35	1353.0	11.7
ESCC151	ESCC	CHCAMS	Female	67	1254.0	10.9
ESCC152	ESCC	CHCAMS	Female	59	1678.9	14.6
ESCC153	ESCC	CHCAMS	Female	55	1188.9	10.4
ESCC154	ESCC	CHCAMS	Male	28	1808.0	15.8
ESCC155	ESCC	CHCAMS	Male	26	917.0	8.0
ESCC156	ESCC	CHCAMS	Female	48	1069.0	9.4
ESCC157	ESCC	CHCAMS	Male	38	1269.0	11.2
ESCC158	ESCC	CHCAMS	Male	39	1300.0	11.5
ESCC159	ESCC	CHCAMS	Female	62	1468.0	13.3
ESCC160	ESCC	CHCAMS	Female	45	1293.0	11.8
ESCC161	ESCC	CHCAMS	Female	37	892.9	8.2
ESCC162	ESCC	CHCAMS	Female	37	1177.1	10.8
ESCC163	ESCC	CHCAMS	Female	48	336.0	3.1
ESCC164	ESCC	CHCAMS	Male	62	1914.9	17.7
ESCC165	ESCC	CHCAMS	Female	74	823.0	7.6
ESCC166	ESCC	CHCAMS	Male	48	1450.1	13.5
ESCC167	ESCC	CHCAMS	Male	62	1457.0	13.7
ESCC168	ESCC	CHCAMS	Male	32	1182.0	11.1
ESCC169	ESCC	CHCAMS	Male	60	883.0	8.3
ESCC170	ESCC	CHCAMS	Male	49	1178.0	11.2
ESCC171	ESCC	CHCAMS	Female	64	2321.0	22.2
ESCC172	ESCC	CHCAMS	Female	42	1610.0	15.4
ESCC173	ESCC	CHCAMS	Male	33	1349.0	12.9
ESCC174	ESCC	CHCAMS	Male	64	1369.1	13.2
ESCC175	ESCC	CHCAMS	Male	35	1097.0	10.8
ESCC176	ESCC	CHCAMS	Male	61	2241.0	22.1
ESCC177	ESCC	CHCAMS	Female	54	1268.0	12.6
ESCC178	ESCC	CHCAMS	Female	39	1261.1	12.6
ESCC179	ESCC	CHCAMS	Female	67	1072.0	10.8
ESCC180	ESCC	CHCAMS	Female	72	1797.0	18.3
ESCC181	ESCC	CHCAMS	Female	30	1055.0	10.8

ESCC182	ESCC	CHCAMS	Male	58	1258.0	12.9
ESCC183	ESCC	CHCAMS	Male	63	1083.9	11.1
ESCC184	ESCC	CHCAMS	Male	43	1200.0	12.3
ESCC185	ESCC	CHCAMS	Male	42	1078.0	11.1
ESCC186	ESCC	CHCAMS	Male	32	1374.0	14.2
ESCC187	ESCC	CHCAMS	Female	43	1097.0	11.4
ESCC188	ESCC	CHCAMS	Female	26	943.0	9.9
ESCC189	ESCC	CHCAMS	Male	25	1071.0	11.3
ESCC190	ESCC	CHCAMS	Female	31	953.0	10.0
ESCC191	ESCC	CHCAMS	Female	59	1311.0	13.9
ESCC192	ESCC	CHCAMS	Female	43	1410.0	15.0
ESCC193	ESCC	CHCAMS	Male	30	1078.0	11.7
ESCC194	ESCC	CHCAMS	Female	40	955.0	10.4
ESCC195	ESCC	CHCAMS	Female	58	1221.0	13.3
ESCC196	ESCC	CHCAMS	Male	53	1256.0	13.8
ESCC197	ESCC	CHCAMS	Female	37	1144.0	12.7
ESCC198	ESCC	CHCAMS	Male	64	2254.0	25.1
ESCC199	ESCC	CHCAMS	Male	34	1277.0	14.5
ESCC200	ESCC	CHCAMS	Male	26	1855.9	21.3
ESCC201	ESCC	CHCAMS	Female	53	1088.0	12.6
ESCC202	ESCC	CHCAMS	Male	62	1105.0	12.9
ESCC203	ESCC	CHCAMS	Male	46	1337.0	15.9
ESCC204	ESCC	CHCAMS	Female	69	1293.9	15.5
ESCC205	ESCC	CHCAMS	Female	32	1216.9	14.7
ESCC206	ESCC	CHCAMS	Male	30	1131.0	13.7
ESCC207	ESCC	CHCAMS	Female	67	1732.0	21.2
ESCC208	ESCC	CHCAMS	Male	27	507.0	6.2
ESCC209	ESCC	CHCAMS	Male	69	602.0	7.5
ESCC210	ESCC	CHCAMS	Female	63	1450.0	18.1
ESCC211	ESCC	CHCAMS	Male	46	1363.0	17.1
ESCC212	ESCC	CHCAMS	Male	68	1391.0	17.5
ESCC213	ESCC	CHCAMS	Male	47	1214.0	15.3
ESCC214	ESCC	CHCAMS	Female	59	2314.0	29.4
ESCC215	ESCC	CHCAMS	Male	26	1593.9	20.3
ESCC216	ESCC	CHCAMS	Female	32	938.9	12.1
ESCC217	ESCC	CHCAMS	Male	61	1206.0	15.7
ESCC218	ESCC	CHCAMS	Male	60	1198.0	15.8
ESCC219	ESCC	CHCAMS	Female	74	1441.0	19.0
ESCC220	ESCC	CHCAMS	Female	27	1687.0	22.4
ESCC221	ESCC	CHCAMS	Male	25	1055.0	14.1
ESCC222	ESCC	CHCAMS	Female	28	1240.0	16.8
ESCC223	ESCC	CHCAMS	Male	52	1352.0	18.3
ESCC224	ESCC	CHCAMS	Male	63	331.0	4.6
ESCC225	ESCC	CHCAMS	Male	55	910.0	13.1

ESCC226	ESCC	CHCAMS	Female	45	650.0	9.4
ESCC227	ESCC	CHCAMS	Male	71	645.9	9.3
ESCC228	ESCC	CHCAMS	Female	50	878.9	12.8
ESCC229	ESCC	CHCAMS	Female	33	935.1	13.7
ESCC230	ESCC	CHCAMS	Male	30	1158.0	17.1
ESCC231	ESCC	CHCAMS	Female	73	1141.0	16.9
ESCC232	ESCC	CHCAMS	Male	72	847.0	12.6
ESCC233	ESCC	CHCAMS	Female	59	1369.0	20.6
ESCC234	ESCC	CHCAMS	Female	72	1426.0	21.5
ESCC235	ESCC	CHCAMS	Female	37	239.0	3.6
ESCC236	ESCC	CHCAMS	Male	47	845.0	13.1
ESCC237	ESCC	CHCAMS	Male	38	826.0	12.9
ESCC238	ESCC	CHCAMS	Female	25	1036.0	16.5
ESCC239	ESCC	CHCAMS	Female	70	922.0	14.8
ESCC240	ESCC	CHCAMS	Male	41	594.0	9.7
ESCC241	ESCC	CHCAMS	Female	50	1392.0	23.3
ESCC242	ESCC	CHCAMS	Male	29	1469.0	24.6
ESCC243	ESCC	CHCAMS	Female	66	1256.0	21.2
ESCC244	ESCC	CHCAMS	Male	69	1241.0	20.9
ESCC245	ESCC	CHCAMS	Male	32	1046.0	17.7
ESCC246	ESCC	CHCAMS	Female	38	631.0	10.7
ESCC247	ESCC	CHCAMS	Female	58	1127.0	19.2
ESCC248	ESCC	CHCAMS	Male	67	1130.0	19.5
ESCC249	ESCC	CHCAMS	Female	46	1173.0	20.5
ESCC250	ESCC	CHCAMS	Female	74	835.0	15.6
ESCC251	ESCC	CHCAMS	Male	70	1090.0	20.5
ESCC252	ESCC	CHCAMS	Male	55	821.0	15.9
ESCC253	ESCC	CHCAMS	Female	35	1077.0	21.1
ESCC254	ESCC	CHCAMS	Female	54	655.0	12.8
ESCC255	ESCC	CHCAMS	Male	46	664.0	13.1
ESCC256	ESCC	CHCAMS	Male	35	530.0	10.4
ESCC257	ESCC	CHCAMS	Female	37	1344.0	26.9
ESCC258	ESCC	CHCAMS	Female	27	361.0	7.4
ESCC259	ESCC	CHCAMS	Male	57	1088.0	23.4
ESCC260	ESCC	CHCAMS	Male	56	1050.0	23.1
ESCC261	ESCC	CHCAMS	Male	44	1061.0	24.1
ESCC262	ESCC	CHCAMS	Female	65	457.0	10.4
ESCC263	ESCC	CHCAMS	Female	65	833.0	19.3
ESCC264	ESCC	CHCAMS	Male	46	647.0	15.7
ESCC265	ESCC	CHCAMS	Female	49	491.9	12.1
ESCC266	ESCC	CHCAMS	Female	26	1557.9	38.4
ESCC267	ESCC	CHCAMS	Female	48	124.6	3.2
ESCC268	ESCC	CHCAMS	Female	55	859.0	22.9
ESCC269	ESCC	CHCAMS	Female	44	285.0	7.8

ESCC270	ESCC	CHCAMS	Female	47	667.0	24.8
ESCC271	ESCC	CHCAMS	Male	45	330.0	14.2
ESCC272	ESCC	CHCAMS	Male	65	545.0	27.6
ESCC273	ESCC	CHCAMS	Female	29	296.0	18.4
ESCC274	ESCC	CHCAMS	Male	70	194.0	13.1
ESCC275	ESCC	CHCAMS	Male	54	307.0	21.7
ESCC276	ESCC	CHCAMS	Male	44	152.9	7.1
GCC1	GCC	CHCAMS	Male	28	120.2	9.2
GCC2	GCC	CHCAMS	Male	27	153.6	3.2
GCC3	GCC	CHCAMS	Female	74	152.5	9.6
GCC4	GCC	CHCAMS	Male	62	166.7	1.9
GCC5	GCC	CHCAMS	Male	27	189	8.6
GCC6	GCC	CHCAMS	Female	72	226.3	13.9
GCC7	GCC	CHCAMS	Female	73	212.9	10.9
GCC8	GCC	CHCAMS	Female	30	212	11.5
GCC9	GCC	CHCAMS	Female	69	215.5	6.8
GCC10	GCC	CHCAMS	Male	65	219.1	12.0
GCC11	GCC	CHCAMS	Female	55	227.3	1.7
GCC12	GCC	CHCAMS	Female	53	232.4	6.0
GCC13	GCC	CHCAMS	Male	59	226	13.2
GCC14	GCC	CHCAMS	Female	62	232.5	3.1
GCC15	GCC	CHCAMS	Male	62	263.4	2.4
GCC16	GCC	CHCAMS	Female	63	271.9	5.5
GCC17	GCC	CHCAMS	Male	68	285.3	8.8
GCC18	GCC	CHCAMS	Male	41	311.9	15.0
GCC19	GCC	CHCAMS	Female	44	297.6	0.9
GCC20	GCC	CHCAMS	Female	74	339.1	12.0
GCC21	GCC	CHCAMS	Male	69	384.9	29.4
GCC22	GCC	CHCAMS	Male	47	372	4.8
GCC23	GCC	CHCAMS	Male	35	392.9	14.7
GCC24	GCC	CHCAMS	Female	55	534.3	7.7
GCC25	GCC	CHCAMS	Male	53	561.9	18.4
GCC26	GCC	CHCAMS	Male	73	547.8	18.1
GCC27	GCC	CHCAMS	Male	36	556.4	1.0
GCC28	GCC	CHCAMS	Female	39	569	5.3
GCC29	GCC	CHCAMS	Female	75	574.7	6.0
GCC30	GCC	CHCAMS	Male	70	571.2	13.7
GCC31	GCC	CHCAMS	Female	69	594.7	6.0
GCC32	GCC	CHCAMS	Male	66	599.5	7.9
GCC33	GCC	CHCAMS	Male	39	302.8	5.6
GCC34	GCC	CHCAMS	Female	44	318.4	3.6
GCC35	GCC	CHCAMS	Female	54	320.1	6.0
GCC36	GCC	CHCAMS	Female	27	319.1	7.3
GCC37	GCC	CHCAMS	Female	72	333.8	8.7

GCC38	GCC	CHCAMS	Male	74	436	9.9
GCC39	GCC	CHCAMS	Female	51	441.5	12.4
GCC40	GCC	CHCAMS	Female	54	462.7	31.2
GCC41	GCC	CHCAMS	Female	30	459	5.5
GCC42	GCC	CHCAMS	Female	53	649.4	28.5
GCC43	GCC	CHCAMS	Male	60	665.6	5.1
GCC44	GCC	CHCAMS	Male	31	667.3	5.9
GCC45	GCC	CHCAMS	Male	54	670.5	9.5
GCC46	GCC	CHCAMS	Male	59	452.8	7.2
GCC47	GCC	CHCAMS	Female	66	455.8	6.4
GCC48	GCC	CHCAMS	Female	42	463.8	1.0
GCC49	GCC	CHCAMS	Female	61	469.3	7.4
GCC50	GCC	CHCAMS	Female	70	701.4	11.3
GCC51	GCC	CHCAMS	Male	72	694.4	11.7
GCC52	GCC	CHCAMS	Male	34	698	7.3
GCC53	GCC	CHCAMS	Female	47	709.6	8.1
GCC54	GCC	CHCAMS	Male	42	502.4	7.7
GCC55	GCC	CHCAMS	Female	27	509.7	2.6
GCC56	GCC	CHCAMS	Male	49	512.8	3.9
GCC57	GCC	CHCAMS	Female	32	509.8	4.8
GCC58	GCC	CHCAMS	Male	55	515	13.6
GCC59	GCC	CHCAMS	Male	35	518.5	20.8
GCC60	GCC	CHCAMS	Female	38	746	5.5
GCC61	GCC	CHCAMS	Female	36	738.8	18.5
GCC62	GCC	CHCAMS	Female	63	751.3	3.3
GCC63	GCC	CHCAMS	Male	65	752	6.3
GCC64	GCC	CHCAMS	Male	29	764.1	7.0
GCC65	GCC	CHCAMS	Female	33	759.5	4.9
GCC66	GCC	CHCAMS	Female	66	766.5	4.1
GCC67	GCC	CHCAMS	Female	28	766.7	0.9
GCC68	GCC	CHCAMS	Female	36	769.2	2.4
GCC69	GCC	CHCAMS	Female	75	772.7	12.7
GCC70	GCC	CHCAMS	Female	58	784	3.9
GCC71	GCC	CHCAMS	Male	50	786.3	19.2
GCC72	GCC	CHCAMS	Male	73	791.2	7.3
GCC73	GCC	CHCAMS	Male	49	790.9	8.1
GCC74	GCC	CHCAMS	Female	53	801.4	8.0
GCC75	GCC	CHCAMS	Female	69	799.5	3.1
GCC76	GCC	CHCAMS	Male	66	806.2	6.3
GCC77	GCC	CHCAMS	Male	41	835	7.1
GCC78	GCC	CHCAMS	Male	73	851.4	5.2
GCC79	GCC	CHCAMS	Female	41	859.8	22.4
GCC80	GCC	CHCAMS	Male	73	852.8	2.3
GCC81	GCC	CHCAMS	Female	51	857.8	7.8

GCC82	GCC	CHCAMS	Male	39	849.1	6.2
GCC83	GCC	CHCAMS	Female	32	860.8	12.3
GCC84	GCC	CHCAMS	Female	49	866.2	9.2
GCC85	GCC	CHCAMS	Female	44	869.3	4.4
GCC86	GCC	CHCAMS	Male	56	871.7	8.4
GCC87	GCC	CHCAMS	Male	66	886	8.9
GCC88	GCC	CHCAMS	Male	29	882.2	9.0
GCC89	GCC	CHCAMS	Male	67	890.1	18.0
GCC90	GCC	CHCAMS	Female	68	893.6	15.7
GCC91	GCC	CHCAMS	Female	56	926.3	15.7
GCC92	GCC	CHCAMS	Male	37	923.2	15.8
GCC93	GCC	CHCAMS	Male	36	913.8	6.1
GCC94	GCC	CHCAMS	Male	54	914.4	14.2
GCC95	GCC	CHCAMS	Female	73	931.1	5.7
GCC96	GCC	CHCAMS	Male	39	932.2	1.2
GCC97	GCC	CHCAMS	Female	28	940.4	11.4
GCC98	GCC	CHCAMS	Male	42	942.2	2.8
GCC99	GCC	CHCAMS	Male	75	952.2	13.7
GCC100	GCC	CHCAMS	Male	43	944.9	6.2
GCC101	GCC	CHCAMS	Male	36	955.4	3.3
GCC102	GCC	CHCAMS	Female	74	953.6	7.7
GCC103	GCC	CHCAMS	Male	74	963.1	5.2
GCC104	GCC	CHCAMS	Male	31	971.5	8.1
GCC105	GCC	CHCAMS	Male	47	974.1	7.4
GCC106	GCC	CHCAMS	Male	36	969.7	5.7
GCC107	GCC	CHCAMS	Male	31	966.8	6.7
GCC108	GCC	CHCAMS	Male	46	989	6.6
GCC109	GCC	CHCAMS	Male	59	994.3	7.6
GCC110	GCC	CHCAMS	Male	73	990	4.4
GCC111	GCC	CHCAMS	Female	50	993	9.7
GCC112	GCC	CHCAMS	Male	60	1024.8	12.3
GCC113	GCC	CHCAMS	Male	68	1029	9.3
GCC114	GCC	CHCAMS	Female	32	1023.4	2.5
GCC115	GCC	CHCAMS	Female	72	1036	9.1
GCC116	GCC	CHCAMS	Male	63	1038.4	2.1
GCC117	GCC	CHCAMS	Male	48	1040.4	4.9
GCC118	GCC	CHCAMS	Male	31	1032.5	15.7
GCC119	GCC	CHCAMS	Male	67	1043	6.9
GCC120	GCC	CHCAMS	Male	60	1060.4	23.5
GCC121	GCC	CHCAMS	Male	53	1036.5	14.4
GCC122	GCC	CHCAMS	Male	41	1053	19.7
GCC123	GCC	CHCAMS	Male	72	1058	6.2
GCC124	GCC	CHCAMS	Male	72	1056.4	7.7
GCC125	GCC	CHCAMS	Male	26	1058.3	8.6

GCC126	GCC	CHCAMS	Male	42	1078.1	19.2
GCC127	GCC	CHCAMS	Female	68	1076.9	7.7
GCC128	GCC	CHCAMS	Female	36	1074.4	5.1
GCC129	GCC	CHCAMS	Female	70	1080.4	5.8
GCC130	GCC	CHCAMS	Male	72	1079.4	11.7
GCC131	GCC	CHCAMS	Male	64	1098.9	29.6
GCC132	GCC	CHCAMS	Male	34	1094.1	9.8
GCC133	GCC	CHCAMS	Female	73	1100.2	4.7
GCC134	GCC	CHCAMS	Male	62	1100.2	14.4
GCC135	GCC	CHCAMS	Female	65	1106.6	6.5
GCC136	GCC	CHCAMS	Female	72	1109.7	18.1
GCC137	GCC	CHCAMS	Male	62	1117.4	8.5
GCC138	GCC	CHCAMS	Male	75	1115.4	4.0
GCC139	GCC	CHCAMS	Male	74	1119.4	11.9
GCC140	GCC	CHCAMS	Male	35	1129.1	0.6
GCC141	GCC	CHCAMS	Male	31	1126.5	19.5
GCC142	GCC	CHCAMS	Male	59	1127.5	9.3
GCC143	GCC	CHCAMS	Male	30	1132.3	6.7
GCC144	GCC	CHCAMS	Female	62	1139.9	11.4
GCC145	GCC	CHCAMS	Female	66	1144.2	7.2
GCC146	GCC	CHCAMS	Female	72	1147	6.7
GCC147	GCC	CHCAMS	Male	47	1168.1	15.7
GCC148	GCC	CHCAMS	Male	62	1158.6	8.4
GCC149	GCC	CHCAMS	Male	56	1160.1	14.2
GCC150	GCC	CHCAMS	Male	45	1155.1	9.8
GCC151	GCC	CHCAMS	Male	53	1172.7	1.5
GCC152	GCC	CHCAMS	Male	74	1175.9	29.4
GCC153	GCC	CHCAMS	Male	42	1182.3	3.7
GCC154	GCC	CHCAMS	Female	30	1187	21.8
GCC155	GCC	CHCAMS	Female	74	1191.7	7.3
GCC156	GCC	CHCAMS	Male	26	1192.1	2.6
GCC157	GCC	CHCAMS	Female	73	1194.6	4.4
GCC158	GCC	CHCAMS	Male	27	1217.7	13.2
GCC159	GCC	CHCAMS	Male	37	1230.5	6.0
GCC160	GCC	CHCAMS	Female	58	1238.6	5.3
GCC161	GCC	CHCAMS	Female	32	1238.4	10.2
GCC162	GCC	CHCAMS	Male	58	1233	22.6
GCC163	GCC	CHCAMS	Female	30	1244	3.1
GCC164	GCC	CHCAMS	Male	72	1263.9	14.0
GCC165	GCC	CHCAMS	Male	33	1252.1	23.9
GCC166	GCC	CHCAMS	Female	33	1260.3	8.0
GCC167	GCC	CHCAMS	Female	53	1257.4	8.0
GCC168	GCC	CHCAMS	Male	40	1258.2	9.3
GCC169	GCC	CHCAMS	Male	66	1263.1	5.8

GCC170	GCC	CHCAMS	Female	74	1263	11.7
GCC171	GCC	CHCAMS	Male	32	1263.2	13.3
GCC172	GCC	CHCAMS	Male	67	1268.7	10.9
GCC173	GCC	CHCAMS	Female	59	1268.6	19.2
GCC174	GCC	CHCAMS	Male	41	1272.9	5.3
GCC175	GCC	CHCAMS	Male	61	1286.5	17.9
GCC176	GCC	CHCAMS	Female	62	1283.8	3.6
GCC177	GCC	CHCAMS	Male	61	1295.8	4.6
GCC178	GCC	CHCAMS	Male	32	1303.8	17.2
GCC179	GCC	CHCAMS	Female	31	1289.9	28.7
GCC180	GCC	CHCAMS	Female	33	1291.4	14.0
GCC181	GCC	CHCAMS	Female	29	1303.6	9.5
GCC182	GCC	CHCAMS	Female	65	1308.8	6.6
GCC183	GCC	CHCAMS	Female	71	1301.4	7.3
GCC184	GCC	CHCAMS	Male	64	1303.6	4.4
GCC185	GCC	CHCAMS	Female	63	1319.5	23.6
GCC186	GCC	CHCAMS	Male	67	1340.8	15.2
GCC187	GCC	CHCAMS	Male	58	1327.9	8.1
GCC188	GCC	CHCAMS	Male	37	1327.5	11.9
GCC189	GCC	CHCAMS	Female	40	1331.8	4.5
GCC190	GCC	CHCAMS	Female	32	1331.5	11.3
GCC191	GCC	CHCAMS	Female	61	1348	16.6
GCC192	GCC	CHCAMS	Male	65	1368.3	10.0
GCC193	GCC	CHCAMS	Female	65	1384.8	8.6
GCC194	GCC	CHCAMS	Male	43	1397.1	16.3
GCC195	GCC	CHCAMS	Female	39	1403.7	15.2
GCC196	GCC	CHCAMS	Male	40	1401	12.0
GCC197	GCC	CHCAMS	Female	57	1401.5	9.0
GCC198	GCC	CHCAMS	Female	59	1407.5	10.2
GCC199	GCC	CHCAMS	Male	69	1409.3	2.2
GCC200	GCC	CHCAMS	Female	45	1416.7	24.5
GCC201	GCC	CHCAMS	Male	27	1411.5	16.3
GCC202	GCC	CHCAMS	Female	63	1420.5	8.5
GCC203	GCC	CHCAMS	Female	65	1422.6	4.2
GCC204	GCC	CHCAMS	Female	36	1426.1	3.4
GCC205	GCC	CHCAMS	Male	51	1429.8	11.4
GCC206	GCC	CHCAMS	Male	56	1434.1	6.6
GCC207	GCC	CHCAMS	Male	72	1425.8	20.4
GCC208	GCC	CHCAMS	Female	29	1449.1	12.8
GCC209	GCC	CHCAMS	Male	69	1446.5	6.0
GCC210	GCC	CHCAMS	Male	73	1450	7.3
GCC211	GCC	CHCAMS	Male	56	1543.8	11.6
GCC212	GCC	CHCAMS	Male	30	1546.3	9.2
GCC213	GCC	CHCAMS	Female	58	1577.6	8.5

GCC214	GCC	CHCAMS	Male	65	1592.2	15.0
GCC215	GCC	CHCAMS	Male	71	1616.8	29.1
GCC216	GCC	CHCAMS	Male	58	1623	13.2
GCC217	GCC	CHCAMS	Female	62	1632.8	8.1
GCC218	GCC	CHCAMS	Male	57	1667.8	4.3
GCC219	GCC	CHCAMS	Male	56	1682.2	6.8
GCC220	GCC	CHCAMS	Female	39	1715.3	16.7
GCC221	GCC	CHCAMS	Female	50	1739.7	13.3
GCC222	GCC	CHCAMS	Female	38	1780.1	13.7
GCC223	GCC	CHCAMS	Female	58	1773.7	14.1
GCC224	GCC	CHCAMS	Male	41	1818.1	6.8
GCC225	GCC	CHCAMS	Male	56	1838.7	4.8
GCC226	GCC	CHCAMS	Male	62	1869.8	10.8
GCC227	GCC	CHCAMS	Male	44	1883.5	22.1
GCC228	GCC	CHCAMS	Female	36	1897.1	10.8
GCC229	GCC	CHCAMS	Male	55	1910.1	10.3
GCC230	GCC	CHCAMS	Female	40	1912.5	3.3
GCC231	GCC	CHCAMS	Male	56	1934.7	7.5
GCC232	GCC	CHCAMS	Female	49	1949.4	7.1
GCC233	GCC	CHCAMS	Female	36	1955.1	11.5
GCC234	GCC	CHCAMS	Female	57	1985.2	16.6
GCC235	GCC	CHCAMS	Female	53	2013.1	1.8
GCC236	GCC	CHCAMS	Male	39	2062.9	11.6
GCC237	GCC	CHCAMS	Male	40	2085.2	16.7
GCC238	GCC	CHCAMS	Male	43	2139.7	12.4
GCC239	GCC	CHCAMS	Female	57	2156.6	29.6
GCC240	GCC	CHCAMS	Female	72	2158	4.1
GCC241	GCC	CHCAMS	Female	56	2171.6	9.1
GCC242	GCC	CHCAMS	Female	53	2166.6	15.1
GCC243	GCC	CHCAMS	Male	61	2266.6	17.6
GCC244	GCC	CHCAMS	Female	48	2335.4	12.8
GCC245	GCC	CHCAMS	Male	52	2335.6	14.6
GCC246	GCC	CHCAMS	Female	53	2425.5	9.5
GCC247	GCC	CHCAMS	Female	44	2433.4	13.5
GCC248	GCC	CHCAMS	Female	60	2771.8	5.9
GC1	GC	CHCAMS	Male	74	941.6	4.0
GC2	GC	CHCAMS	Male	27	1222.7	8.9
GC3	GC	CHCAMS	Male	75	1247	11.1
GC4	GC	CHCAMS	Female	68	1368.5	14.2
GC5	GC	CHCAMS	Female	42	692.8	14.4
GC6	GC	CHCAMS	Male	54	1010.8	3.3
GC7	GC	CHCAMS	Male	26	1055.6	6.0
GC8	GC	CHCAMS	Female	49	1380.4	7.9
GC9	GC	CHCAMS	Female	49	1032.8	6.2

GC10	GC	CHCAMS	Male	29	618.1	3.9
GC11	GC	CHCAMS	Female	31	1306.9	9.1
GC12	GC	CHCAMS	Male	52	1117.7	11.2
GC13	GC	CHCAMS	Male	68	640.8	7.3
GC14	GC	CHCAMS	Female	48	577.5	6.6
GC15	GC	CHCAMS	Female	48	938.7	11.4
GC16	GC	CHCAMS	Female	62	659.9	12.6
GC17	GC	CHCAMS	Female	41	340.3	8.2
GC18	GC	CHCAMS	Female	28	436.3	12.3
GC19	GC	CHCAMS	Male	50	641.6	20.5
GC20	GC	CHCAMS	Female	53	150.1	7.7
GC21	GC	CHCAMS	Male	47	779.7	3.1
GC22	GC	CHCAMS	Female	74	765.8	2.9
GC23	GC	CHCAMS	Male	72	1831.7	9.7
GC24	GC	CHCAMS	Male	28	969.1	10.7
GC25	GC	CHCAMS	Female	45	905.6	20.6
GC26	GC	CHCAMS	Female	50	1378.4	18.4
GC27	GC	CHCAMS	Female	61	726.9	15.9
GC28	GC	CHCAMS	Female	49	1052.1	3.5
GC29	GC	CHCAMS	Female	65	1167.7	5.2
GC30	GC	CHCAMS	Female	38	929.9	5.2
GC31	GC	CHCAMS	Male	34	1448.8	8.8
GC32	GC	CHCAMS	Female	65	1081.2	6.9
GC33	GC	CHCAMS	Female	61	993.1	7.9
GC34	GC	CHCAMS	Female	73	1100.5	8.9
GC35	GC	CHCAMS	Female	69	2372.3	21.5
GC36	GC	CHCAMS	Male	67	1022.1	9.3
GC37	GC	CHCAMS	Male	44	848.7	8.6
GC38	GC	CHCAMS	Male	60	550.9	5.8
GC39	GC	CHCAMS	Male	38	1080.5	13.2
GC40	GC	CHCAMS	Male	49	1146.3	18.8
GC41	GC	CHCAMS	Male	30	413.7	7.9
GC42	GC	CHCAMS	Female	28	828.2	16.3
GC43	GC	CHCAMS	Male	71	718.3	14.3
GC44	GC	CHCAMS	Female	54	773.6	22.9
GC45	GC	CHCAMS	Female	70	227.8	8.7
GC46	GC	CHCAMS	Male	43	229.3	11.1
GC47	GC	CHCAMS	Male	28	197.5	9.7
GC48	GC	CHCAMS	Male	32	503	19.8
GC49	GC	CHCAMS	Male	73	881.3	5.5
GC50	GC	CHCAMS	Male	62	142.9	10.2
GC51	GC	CHCAMS	Male	43	1229.5	11.0
GC52	GC	CHCAMS	Female	70	925.8	6.2
GC53	GC	CHCAMS	Female	56	592.1	4.1

GC54	GC	CHCAMS	Female	35	383.8	5.6
GC55	GC	CHCAMS	Female	60	1758.8	6.3
GC56	GC	CHCAMS	Male	68	1385.4	9.2
GC57	GC	CHCAMS	Female	43	535.8	6.7
GC58	GC	CHCAMS	Female	28	500.3	7.0
GC59	GC	CHCAMS	Female	31	921.4	12.9
GC60	GC	CHCAMS	Male	68	1236.9	3.1
GC61	GC	CHCAMS	Male	53	782.9	2.4
GC62	GC	CHCAMS	Male	64	930.4	3.0
GC63	GC	CHCAMS	Female	69	1273.1	4.6
GC64	GC	CHCAMS	Male	44	1003.8	3.7
GC65	GC	CHCAMS	Female	27	1629.1	6.6
GC66	GC	CHCAMS	Female	58	1063.4	4.6
GC67	GC	CHCAMS	Male	35	1220.2	5.3
GC68	GC	CHCAMS	Male	38	1525.2	7.5
GC69	GC	CHCAMS	Female	45	1264.3	6.5
GC70	GC	CHCAMS	Female	41	1393.5	7.2
GC71	GC	CHCAMS	Male	26	968.5	5.0
GC72	GC	CHCAMS	Female	32	1768.6	9.1
GC73	GC	CHCAMS	Female	34	905.8	4.7
GC74	GC	CHCAMS	Male	31	1255	6.7
GC75	GC	CHCAMS	Female	34	1751.4	10.1
GC76	GC	CHCAMS	Female	70	1317.2	7.8
GC77	GC	CHCAMS	Male	47	467.3	2.8
GC78	GC	CHCAMS	Male	62	1271	7.8
GC79	GC	CHCAMS	Male	26	1530.2	9.6
GC80	GC	CHCAMS	Male	51	1289.2	8.2
GC81	GC	CHCAMS	Female	46	990.9	6.4
GC82	GC	CHCAMS	Male	48	1090.7	7.6
GC83	GC	CHCAMS	Male	37	1389.7	9.7
GC84	GC	CHCAMS	Male	36	975	7.0
GC85	GC	CHCAMS	Female	38	1111.9	8.0
GC86	GC	CHCAMS	Male	47	1820.3	13.1
GC87	GC	CHCAMS	Female	66	579.3	4.1
GC88	GC	CHCAMS	Female	51	567.3	4.1
GC89	GC	CHCAMS	Male	29	1071.3	7.9
GC90	GC	CHCAMS	Female	59	1926.2	14.5
GC91	GC	CHCAMS	Male	43	826.3	6.4
GC92	GC	CHCAMS	Female	35	740.3	5.8
GC93	GC	CHCAMS	Male	37	1043.1	8.3
GC94	GC	CHCAMS	Female	60	740.4	5.9
GC95	GC	CHCAMS	Male	71	907.3	7.3
GC96	GC	CHCAMS	Male	60	441.9	3.5
GC97	GC	CHCAMS	Male	65	883.9	7.2

GC98	GC	CHCAMS	Male	40	871.3	7.1
GC99	GC	CHCAMS	Male	67	834.6	7.0
GC100	GC	CHCAMS	Male	55	1326.6	11.2
GC101	GC	CHCAMS	Female	44	1279.1	10.9
GC102	GC	CHCAMS	Female	36	681.1	5.8
GC103	GC	CHCAMS	Male	58	1921.1	16.8
GC104	GC	CHCAMS	Female	65	1062.2	9.4
GC105	GC	CHCAMS	Female	69	935.8	8.4
GC106	GC	CHCAMS	Male	65	934.9	8.5
GC107	GC	CHCAMS	Female	69	1195.3	10.8
GC108	GC	CHCAMS	Male	36	1020.7	9.3
GC109	GC	CHCAMS	Female	41	996.7	9.3
GC110	GC	CHCAMS	Male	48	1822.3	17.0
GC111	GC	CHCAMS	Female	51	1294.7	12.1
GC112	GC	CHCAMS	Female	38	701.4	6.6
GC113	GC	CHCAMS	Female	55	208.5	2.0
GC114	GC	CHCAMS	Male	39	854.5	8.2
GC115	GC	CHCAMS	Male	38	963.1	9.2
GC116	GC	CHCAMS	Male	34	954.8	9.2
GC117	GC	CHCAMS	Male	50	867.3	8.5
GC118	GC	CHCAMS	Male	27	1363.4	13.3
GC119	GC	CHCAMS	Female	28	864.4	8.6
GC120	GC	CHCAMS	Female	29	663.7	6.7
GC121	GC	CHCAMS	Female	56	1076.6	10.8
GC122	GC	CHCAMS	Male	69	1211.7	12.3
GC123	GC	CHCAMS	Female	48	545.8	5.6
GC124	GC	CHCAMS	Female	48	574.2	6.0
GC125	GC	CHCAMS	Male	43	818.4	8.5
GC126	GC	CHCAMS	Female	55	1111.3	11.6
GC127	GC	CHCAMS	Male	74	1736.1	18.5
GC128	GC	CHCAMS	Male	30	716.8	7.6
GC129	GC	CHCAMS	Male	49	808.1	8.6
GC130	GC	CHCAMS	Female	33	2393.5	25.5
GC131	GC	CHCAMS	Male	35	1226.5	13.1
GC132	GC	CHCAMS	Male	70	1216.1	13.0
GC133	GC	CHCAMS	Female	62	1686.7	18.1
GC134	GC	CHCAMS	Female	50	806.2	8.7
GC135	GC	CHCAMS	Female	56	787.9	8.5
GC136	GC	CHCAMS	Female	59	746.4	8.2
GC137	GC	CHCAMS	Male	61	1018.3	11.3
GC138	GC	CHCAMS	Female	66	1046.6	11.6
GC139	GC	CHCAMS	Male	27	1416.1	15.8
GC140	GC	CHCAMS	Male	66	831.3	9.8
GC141	GC	CHCAMS	Female	57	752.7	8.9

GC142	GC	CHCAMS	Female	71	401.9	4.8
GC143	GC	CHCAMS	Female	32	1506	18.0
GC144	GC	CHCAMS	Female	35	1030.9	12.4
GC145	GC	CHCAMS	Male	39	490.8	6.0
GC146	GC	CHCAMS	Male	29	576.4	7.2
GC147	GC	CHCAMS	Male	26	812.8	10.4
GC148	GC	CHCAMS	Male	64	631.1	8.1
GC149	GC	CHCAMS	Male	43	1138.3	14.6
GC150	GC	CHCAMS	Male	41	508.2	6.6
GC151	GC	CHCAMS	Female	56	1254	16.4
GC152	GC	CHCAMS	Male	47	980.6	12.8
GC153	GC	CHCAMS	Male	42	786.3	10.4
GC154	GC	CHCAMS	Male	47	1532.3	20.4
GC155	GC	CHCAMS	Female	39	608.2	8.2
GC156	GC	CHCAMS	Female	63	887.8	11.9
GC157	GC	CHCAMS	Female	34	1104	15.0
GC158	GC	CHCAMS	Male	65	1642.4	22.5
GC159	GC	CHCAMS	Female	74	724.8	10.1
GC160	GC	CHCAMS	Male	38	636.2	9.1
GC161	GC	CHCAMS	Female	70	1286.3	18.5
GC162	GC	CHCAMS	Female	33	1153.7	16.7
GC163	GC	CHCAMS	Female	57	370.2	5.4
GC164	GC	CHCAMS	Male	58	451.6	6.6
GC165	GC	CHCAMS	Male	61	921	13.7
GC166	GC	CHCAMS	Male	42	1488.9	22.3
GC167	GC	CHCAMS	Male	55	1301.9	20.1
GC168	GC	CHCAMS	Female	56	599.2	9.2
GC169	GC	CHCAMS	Female	71	557.7	8.7
GC170	GC	CHCAMS	Male	54	1204.6	19.1
GC171	GC	CHCAMS	Female	59	514.1	8.2
GC172	GC	CHCAMS	Male	37	858.9	13.7
GC173	GC	CHCAMS	Female	58	1189.9	19.2
GC174	GC	CHCAMS	Female	68	704	11.7
GC175	GC	CHCAMS	Female	26	793.2	13.3
GC176	GC	CHCAMS	Male	33	746.7	12.7
GC177	GC	CHCAMS	Male	44	1019.5	17.9
GC178	GC	CHCAMS	Male	66	559.8	9.9
GC179	GC	CHCAMS	Male	37	1371.5	24.4
GC180	GC	CHCAMS	Male	35	456	8.1
GC181	GC	CHCAMS	Male	38	815.9	14.7
GC182	GC	CHCAMS	Female	58	238.2	4.3
GC183	GC	CHCAMS	Male	59	520.8	9.9
GC184	GC	CHCAMS	Male	55	522.5	10.0
GC185	GC	CHCAMS	Male	36	758.7	14.7

GC186	GC	CHCAMS	Male	25	958.1	18.6
GC187	GC	CHCAMS	Male	71	505.3	9.9
GC188	GC	CHCAMS	Female	46	340	6.7
GC189	GC	CHCAMS	Male	46	518.3	10.2
GC190	GC	CHCAMS	Male	66	482.8	9.6
GC191	GC	CHCAMS	Male	31	469.3	10.0
GC192	GC	CHCAMS	Male	29	873.4	18.7
GC193	GC	CHCAMS	Female	43	245.9	5.3
GC194	GC	CHCAMS	Male	42	376.3	8.1
GC195	GC	CHCAMS	Male	63	393	8.5
GC196	GC	CHCAMS	Female	54	917.2	20.3
GC197	GC	CHCAMS	Male	57	816.3	18.7
GC198	GC	CHCAMS	Male	41	707.2	16.3
GC199	GC	CHCAMS	Male	59	499.3	11.6
GC200	GC	CHCAMS	Female	54	973.7	23.0
GC201	GC	CHCAMS	Male	63	591.6	14.2
GC202	GC	CHCAMS	Male	30	348.4	8.9
GC203	GC	CHCAMS	Female	56	352.8	9.0
GC204	GC	CHCAMS	Female	51	537.1	14.0
GC205	GC	CHCAMS	Female	70	624.2	16.8
GC206	GC	CHCAMS	Male	67	381.3	10.5
GC207	GC	CHCAMS	Male	44	406.2	11.2
GC208	GC	CHCAMS	Female	34	516.5	14.2
GC209	GC	CHCAMS	Male	52	229.2	6.4
GC210	GC	CHCAMS	Male	29	381.5	10.7
GC211	GC	CHCAMS	Male	59	500.2	14.2
GC212	GC	CHCAMS	Female	65	602.6	17.5
GC213	GC	CHCAMS	Female	65	545.3	16.1
GC214	GC	CHCAMS	Male	36	1029	30.6
GC215	GC	CHCAMS	Male	57	469.5	14.1
GC216	GC	CHCAMS	Male	74	543.9	16.4
GC217	GC	CHCAMS	Female	57	461.7	14.3
GC218	GC	CHCAMS	Female	26	342.9	10.8
GC219	GC	CHCAMS	Female	65	455.4	14.5
GC220	GC	CHCAMS	Female	30	341.4	11.6
GC221	GC	CHCAMS	Male	36	415.9	14.2
GC222	GC	CHCAMS	Male	39	345.3	12.3
GC223	GC	CHCAMS	Male	72	475.8	17.4
GC224	GC	CHCAMS	Female	34	352.8	13.3
GC225	GC	CHCAMS	Female	41	389.5	15.7
GC226	GC	CHCAMS	Male	55	588.3	23.9
GC227	GC	CHCAMS	Male	68	237.4	10.3
GC228	GC	CHCAMS	Female	47	527.7	23.3
GC229	GC	CHCAMS	Male	69	179.1	8.6

GC230	GC	CHCAMS	Male	46	231	13.9
GC231	GC	CHCAMS	Male	66	181.6	11.6
GC232	GC	CHCAMS	Male	38	247.2	7.5
GC233	GC	CHCAMS	Female	45	195.5	1.9
GC234	GC	CHCAMS	Male	26	165.8	1.5
GC235	GC	CHCAMS	Female	69	121.9	3.5
GC236	GC	CHCAMS	Male	28	110.2	3.6
GC237	GC	CHCAMS	Male	41	110.1	3.1
GC238	GC	CHCAMS	Male	49	905.3	5.7
GC239	GC	CHCAMS	Male	74	1117.1	8.3
GC240	GC	CHCAMS	Female	67	935.4	8.6
GC241	GC	CHCAMS	Female	34	912.9	9.2
GC242	GC	CHCAMS	Male	68	1024	10.4
GC243	GC	CHCAMS	Male	61	511.8	8.6
GC244	GC	CHCAMS	Male	60	500.4	13.6
GC245	GC	CHCAMS	Female	51	632.8	17.8
GC246	GC	CHCAMS	Male	31	375.9	13.6
GC247	GC	CHCAMS	Male	69	1007.8	5.3
GC248	GC	CHCAMS	Female	47	684.2	11.1
GC249	GC	CHCAMS	Male	61	694.8	5.0
GC250	GC	CHCAMS	Male	72	1254.4	6.8
GC251	GC	CHCAMS	Female	38	1501.4	13.5
GC252	GC	CHCAMS	Male	38	551.6	10.5
GC253	GC	CHCAMS	Male	72	232.3	8.2
GC254	GC	CHCAMS	Female	51	1194	18.6
GC255	GC	CHCAMS	Male	71	2050	5.5
GC256	GC	CHCAMS	Female	28	460.2	20.4
GC257	GC	CHCAMS	Female	70	1145.3	5.9
GC258	GC	CHCAMS	Female	49	1078	6.8
GC259	GC	CHCAMS	Female	58	620.8	12.2
PDAC1	PDAC	CHCAMS	Male	38	63.7	3.5
PDAC2	PDAC	CHCAMS	Male	59	121.6	1.0
PDAC3	PDAC	CHCAMS	Male	67	137.1	3.1
PDAC4	PDAC	CHCAMS	Female	58	160.1	3.3
PDAC5	PDAC	CHCAMS	Female	59	161.4	3.8
PDAC6	PDAC	CHCAMS	Female	35	174.1	2.5
PDAC7	PDAC	CHCAMS	Female	37	179	6.2
PDAC8	PDAC	CHCAMS	Female	60	194.4	2.4
PDAC9	PDAC	CHCAMS	Male	30	197.1	2.1
PDAC10	PDAC	CHCAMS	Male	61	205.5	5.5
PDAC11	PDAC	CHCAMS	Female	31	215.3	7.0
PDAC12	PDAC	CHCAMS	Female	35	218.1	1.3
PDAC13	PDAC	CHCAMS	Female	31	224.8	3.2
PDAC14	PDAC	CHCAMS	Female	39	240.8	2.6

PDAC15	PDAC	CHCAMS	Male	71	258.1	3.1
PDAC16	PDAC	CHCAMS	Female	65	259.5	1.4
PDAC17	PDAC	CHCAMS	Male	74	264.1	2.3
PDAC18	PDAC	CHCAMS	Male	51	326.5	3.0
PDAC19	PDAC	CHCAMS	Female	33	329.1	4.2
PDAC20	PDAC	CHCAMS	Female	44	328.9	2.2
PDAC21	PDAC	CHCAMS	Female	48	341.9	6.4
PDAC22	PDAC	CHCAMS	Female	32	343.1	2.4
PDAC23	PDAC	CHCAMS	Female	36	344	6.7
PDAC24	PDAC	CHCAMS	Male	32	344.6	0.5
PDAC25	PDAC	CHCAMS	Female	38	348	2.0
PDAC26	PDAC	CHCAMS	Male	49	351.7	2.0
PDAC27	PDAC	CHCAMS	Female	39	354	2.3
PDAC28	PDAC	CHCAMS	Male	73	360.8	3.7
PDAC29	PDAC	CHCAMS	Female	29	362.2	3.7
PDAC30	PDAC	CHCAMS	Female	29	364.3	3.3
PDAC31	PDAC	CHCAMS	Female	27	364.6	2.8
PDAC32	PDAC	CHCAMS	Female	69	370.8	2.4
PDAC33	PDAC	CHCAMS	Female	45	372.2	1.7
PDAC34	PDAC	CHCAMS	Male	37	371.1	1.0
PDAC35	PDAC	CHCAMS	Female	66	374.8	0.7
PDAC36	PDAC	CHCAMS	Female	46	377.4	2.3
PDAC37	PDAC	CHCAMS	Female	31	383.8	2.4
PDAC38	PDAC	CHCAMS	Female	28	393.8	1.6
PDAC39	PDAC	CHCAMS	Male	42	396.5	1.9
PDAC40	PDAC	CHCAMS	Female	69	396.4	2.0
PDAC41	PDAC	CHCAMS	Male	72	396.4	1.6
PDAC42	PDAC	CHCAMS	Female	27	399	1.9
PDAC43	PDAC	CHCAMS	Female	74	413.4	3.2
PDAC44	PDAC	CHCAMS	Male	40	415.7	1.2
PDAC45	PDAC	CHCAMS	Female	54	417.1	2.8
PDAC46	PDAC	CHCAMS	Female	30	417.8	2.2
PDAC47	PDAC	CHCAMS	Male	31	428.7	5.0
PDAC48	PDAC	CHCAMS	Male	27	429.3	2.3
PDAC49	PDAC	CHCAMS	Female	60	447.8	5.4
PDAC50	PDAC	CHCAMS	Male	47	456.8	2.3
PDAC51	PDAC	CHCAMS	Male	51	463.3	1.6
PDAC52	PDAC	CHCAMS	Female	27	462.9	2.4
PDAC53	PDAC	CHCAMS	Female	72	466	2.3
PDAC54	PDAC	CHCAMS	Male	44	467.2	1.5
PDAC55	PDAC	CHCAMS	Male	40	473.8	1.2
PDAC56	PDAC	CHCAMS	Female	73	480.7	1.8
PDAC57	PDAC	CHCAMS	Male	42	494.3	3.1
PDAC58	PDAC	CHCAMS	Male	52	497.9	5.1

PDAC59	PDAC	CHCAMS	Female	34	496.4	2.1
PDAC60	PDAC	CHCAMS	Male	54	505.4	4.2
PDAC61	PDAC	CHCAMS	Female	67	502.2	2.7
PDAC62	PDAC	CHCAMS	Male	38	507.5	4.1
PDAC63	PDAC	CHCAMS	Male	61	509.7	1.7
PDAC64	PDAC	CHCAMS	Female	34	512.9	5.3
PDAC65	PDAC	CHCAMS	Male	72	513.4	3.0
PDAC66	PDAC	CHCAMS	Male	70	522.4	2.3
PDAC67	PDAC	CHCAMS	Male	69	529.6	2.9
PDAC68	PDAC	CHCAMS	Male	29	527.9	2.3
PDAC69	PDAC	CHCAMS	Female	55	531.1	1.9
PDAC70	PDAC	CHCAMS	Male	37	532.7	2.6
PDAC71	PDAC	CHCAMS	Female	34	533.8	2.6
PDAC72	PDAC	CHCAMS	Male	67	541.6	0.6
PDAC73	PDAC	CHCAMS	Male	51	542.5	0.8
PDAC74	PDAC	CHCAMS	Male	50	549.7	2.0
PDAC75	PDAC	CHCAMS	Female	52	556.5	2.7
PDAC76	PDAC	CHCAMS	Female	35	557.1	1.9
PDAC77	PDAC	CHCAMS	Male	26	563.3	3.9
PDAC78	PDAC	CHCAMS	Male	33	566.3	2.9
PDAC79	PDAC	CHCAMS	Male	55	573	10.1
PDAC80	PDAC	CHCAMS	Male	38	576.5	1.9
PDAC81	PDAC	CHCAMS	Male	26	579.7	1.8
PDAC82	PDAC	CHCAMS	Male	60	585.2	5.4
PDAC83	PDAC	CHCAMS	Male	43	599.1	2.5
PDAC84	PDAC	CHCAMS	Male	71	603.8	4.4
PDAC85	PDAC	CHCAMS	Male	67	616	1.4
PDAC86	PDAC	CHCAMS	Male	50	617.2	10.2
PDAC87	PDAC	CHCAMS	Female	68	618	3.9
PDAC88	PDAC	CHCAMS	Female	42	621.7	1.4
PDAC89	PDAC	CHCAMS	Male	28	623.5	1.9
PDAC90	PDAC	CHCAMS	Male	70	625.3	4.1
PDAC91	PDAC	CHCAMS	Female	48	631	4.5
PDAC92	PDAC	CHCAMS	Male	67	645	7.1
PDAC93	PDAC	CHCAMS	Female	40	640.9	2.8
PDAC94	PDAC	CHCAMS	Female	73	640.3	1.8
PDAC95	PDAC	CHCAMS	Male	52	643.9	2.0
PDAC96	PDAC	CHCAMS	Male	35	651.8	3.2
PDAC97	PDAC	CHCAMS	Male	70	652.7	3.5
PDAC98	PDAC	CHCAMS	Female	35	655.6	2.3
PDAC99	PDAC	CHCAMS	Female	44	664.4	2.3
PDAC100	PDAC	CHCAMS	Female	27	670.3	4.6
PDAC101	PDAC	CHCAMS	Female	32	671.3	2.2
PDAC102	PDAC	CHCAMS	Male	55	670.3	2.1

PDAC103	PDAC	CHCAMS	Female	49	677.1	5.5
PDAC104	PDAC	CHCAMS	Female	74	687.2	3.0
PDAC105	PDAC	CHCAMS	Female	50	691.3	5.8
PDAC106	PDAC	CHCAMS	Male	56	691.9	1.3
PDAC107	PDAC	CHCAMS	Male	46	695.2	4.0
PDAC108	PDAC	CHCAMS	Male	55	695.6	3.2
PDAC109	PDAC	CHCAMS	Female	35	702	0.4
PDAC110	PDAC	CHCAMS	Male	71	702.5	2.0
PDAC111	PDAC	CHCAMS	Male	46	705.9	3.3
PDAC112	PDAC	CHCAMS	Male	38	705.5	1.6
PDAC113	PDAC	CHCAMS	Female	41	710.6	3.7
PDAC114	PDAC	CHCAMS	Female	54	711.2	2.6
PDAC115	PDAC	CHCAMS	Female	36	718.2	4.1
PDAC116	PDAC	CHCAMS	Female	52	721.2	2.7
PDAC117	PDAC	CHCAMS	Male	53	721.6	3.1
PDAC118	PDAC	CHCAMS	Male	52	730.9	3.2
PDAC119	PDAC	CHCAMS	Male	52	738.8	3.4
PDAC120	PDAC	CHCAMS	Male	60	746.9	3.3
PDAC121	PDAC	CHCAMS	Female	34	746.5	0.5
PDAC122	PDAC	CHCAMS	Male	26	758.7	2.3
PDAC123	PDAC	CHCAMS	Female	30	762.3	2.4
PDAC124	PDAC	CHCAMS	Female	51	768.4	3.3
PDAC125	PDAC	CHCAMS	Male	69	767.3	2.6
PDAC126	PDAC	CHCAMS	Female	53	779.2	2.8
PDAC127	PDAC	CHCAMS	Male	38	782.8	3.7
PDAC128	PDAC	CHCAMS	Male	62	784.7	4.6
PDAC129	PDAC	CHCAMS	Female	59	783.7	2.6
PDAC130	PDAC	CHCAMS	Male	68	797	2.1
PDAC131	PDAC	CHCAMS	Male	58	806.9	5.2
PDAC132	PDAC	CHCAMS	Female	43	814	4.7
PDAC133	PDAC	CHCAMS	Female	49	810.4	1.9
PDAC134	PDAC	CHCAMS	Female	68	812.6	1.2
PDAC135	PDAC	CHCAMS	Female	37	825.1	1.7
PDAC136	PDAC	CHCAMS	Male	58	824	0.6
PDAC137	PDAC	CHCAMS	Female	29	845.3	3.1
PDAC138	PDAC	CHCAMS	Female	53	845.5	5.3
PDAC139	PDAC	CHCAMS	Female	40	847.9	2.6
PDAC140	PDAC	CHCAMS	Male	28	849.1	4.1
PDAC141	PDAC	CHCAMS	Female	27	848.5	1.7
PDAC142	PDAC	CHCAMS	Male	70	849.6	1.6
PDAC143	PDAC	CHCAMS	Male	36	862	3.5
PDAC144	PDAC	CHCAMS	Female	69	874.1	3.0
PDAC145	PDAC	CHCAMS	Female	28	875.4	0.8
PDAC146	PDAC	CHCAMS	Male	62	880.7	5.0

PDAC147	PDAC	CHCAMS	Female	46	889.1	3.1
PDAC148	PDAC	CHCAMS	Male	43	887.8	1.0
PDAC149	PDAC	CHCAMS	Male	59	899.5	9.1
PDAC150	PDAC	CHCAMS	Female	53	908.4	1.4
PDAC151	PDAC	CHCAMS	Male	64	914.1	3.5
PDAC152	PDAC	CHCAMS	Female	68	929.3	7.5
PDAC153	PDAC	CHCAMS	Male	60	924.9	3.6
PDAC154	PDAC	CHCAMS	Female	74	927.1	1.6
PDAC155	PDAC	CHCAMS	Male	41	926.2	1.3
PDAC156	PDAC	CHCAMS	Female	72	935.2	1.8
PDAC157	PDAC	CHCAMS	Female	36	950.3	2.5
PDAC158	PDAC	CHCAMS	Male	64	1003.3	2.5
PDAC159	PDAC	CHCAMS	Female	72	1015.2	1.4
PDAC160	PDAC	CHCAMS	Male	47	1018.8	1.3
PDAC161	PDAC	CHCAMS	Female	54	1021.4	3.0
PDAC162	PDAC	CHCAMS	Female	34	1022.1	1.4
PDAC163	PDAC	CHCAMS	Male	40	1048.7	0.9
PDAC164	PDAC	CHCAMS	Male	60	1059.5	5.0
PDAC165	PDAC	CHCAMS	Female	39	1055.2	1.8
PDAC166	PDAC	CHCAMS	Female	53	1069.4	2.5
PDAC167	PDAC	CHCAMS	Female	51	1070.9	4.1
PDAC168	PDAC	CHCAMS	Female	46	1077.9	4.7
PDAC169	PDAC	CHCAMS	Male	73	1090.2	1.8
PDAC170	PDAC	CHCAMS	Female	44	1094.2	1.9
PDAC171	PDAC	CHCAMS	Male	52	1100	1.7
PDAC172	PDAC	CHCAMS	Male	43	1103.8	2.6
PDAC173	PDAC	CHCAMS	Female	57	1112.8	1.3
PDAC174	PDAC	CHCAMS	Female	36	1112.5	1.0
PDAC175	PDAC	CHCAMS	Female	31	1112.9	2.7
PDAC176	PDAC	CHCAMS	Female	43	1119.4	5.0
PDAC177	PDAC	CHCAMS	Male	28	1121.5	2.5
PDAC178	PDAC	CHCAMS	Female	70	1123.8	2.0
PDAC179	PDAC	CHCAMS	Female	52	1127.9	2.2
PDAC180	PDAC	CHCAMS	Male	45	1129.7	1.4
PDAC181	PDAC	CHCAMS	Female	42	1135.7	1.0
PDAC182	PDAC	CHCAMS	Female	56	1145.2	3.1
PDAC183	PDAC	CHCAMS	Male	26	1153.1	4.5
PDAC184	PDAC	CHCAMS	Female	28	1152.4	2.2
PDAC185	PDAC	CHCAMS	Female	33	1153.4	6.0
PDAC186	PDAC	CHCAMS	Female	44	1157	2.3
PDAC187	PDAC	CHCAMS	Male	35	1158	2.1
PDAC188	PDAC	CHCAMS	Female	46	1167.4	2.3
PDAC189	PDAC	CHCAMS	Female	54	1178.3	4.3
PDAC190	PDAC	CHCAMS	Male	36	1187	1.5

PDAC191	PDAC	CHCAMS	Male	26	1190.3	1.3
PDAC192	PDAC	CHCAMS	Male	71	1200	10.5
PDAC193	PDAC	CHCAMS	Female	73	1204.2	3.3
PDAC194	PDAC	CHCAMS	Male	57	1204.5	3.0
PDAC195	PDAC	CHCAMS	Female	48	1209.1	2.6
PDAC196	PDAC	CHCAMS	Female	70	1208.9	1.8
PDAC197	PDAC	CHCAMS	Male	33	1214.2	3.2
PDAC198	PDAC	CHCAMS	Female	53	1217.1	2.1
PDAC199	PDAC	CHCAMS	Female	35	1223.5	1.5
PDAC200	PDAC	CHCAMS	Female	27	1232.5	5.7
PDAC201	PDAC	CHCAMS	Female	55	1236.7	4.0
PDAC202	PDAC	CHCAMS	Female	30	1235.5	1.9
PDAC203	PDAC	CHCAMS	Male	40	1244.5	3.0
PDAC204	PDAC	CHCAMS	Female	28	1246.2	2.7
PDAC205	PDAC	CHCAMS	Male	50	1249.1	0.7
PDAC206	PDAC	CHCAMS	Female	53	1250.1	2.2
PDAC207	PDAC	CHCAMS	Male	69	1259.9	1.4
PDAC208	PDAC	CHCAMS	Male	35	1266.6	1.8
PDAC209	PDAC	CHCAMS	Male	59	1275.8	1.5
PDAC210	PDAC	CHCAMS	Male	65	1277.1	2.5
PDAC211	PDAC	CHCAMS	Female	72	1291.6	2.3
PDAC212	PDAC	CHCAMS	Female	57	1290.3	1.5
PDAC213	PDAC	CHCAMS	Female	35	1308.7	1.6
PDAC214	PDAC	CHCAMS	Male	52	1321.4	1.5
PDAC215	PDAC	CHCAMS	Male	39	1324.9	4.5
PDAC216	PDAC	CHCAMS	Female	52	1323.5	2.3
PDAC217	PDAC	CHCAMS	Female	55	1327.1	3.5
PDAC218	PDAC	CHCAMS	Female	59	1330.2	1.8
PDAC219	PDAC	CHCAMS	Female	66	1333.8	2.8
PDAC220	PDAC	CHCAMS	Female	53	1349.8	2.6
PDAC221	PDAC	CHCAMS	Male	44	1365	6.6
PDAC222	PDAC	CHCAMS	Male	37	1369.7	1.5
PDAC223	PDAC	CHCAMS	Male	46	1118.1	3.1
PDAC224	PDAC	CHCAMS	Female	46	1147.3	4.8
PDAC225	PDAC	CHCAMS	Female	41	1065.5	1.6
PDAC226	PDAC	CHCAMS	Female	65	1118.6	4.4
PDAC227	PDAC	CHCAMS	Female	71	1071.5	6.4
PDAC228	PDAC	CHCAMS	Female	32	1179.4	1.5
PDAC229	PDAC	CHCAMS	Male	31	1218.9	1.8
PDAC230	PDAC	CHCAMS	Female	59	1046.7	4.5
PDAC231	PDAC	CHCAMS	Female	55	1199.6	10.7
PDAC232	PDAC	CHCAMS	Male	33	1088.1	2.6
PDAC233	PDAC	CHCAMS	Female	65	1168	1.9
PDAC234	PDAC	CHCAMS	Female	69	1404.9	2.2

PDAC235	PDAC	CHCAMS	Male	56	1405.9	4.3
PDAC236	PDAC	CHCAMS	Male	37	1420.7	2.5
PDAC237	PDAC	CHCAMS	Female	47	1426.5	3.7
PDAC238	PDAC	CHCAMS	Female	40	1437.8	6.1
PDAC239	PDAC	CHCAMS	Male	36	1439.4	3.5
PDAC240	PDAC	CHCAMS	Male	46	1440.2	3.6
PDAC241	PDAC	CHCAMS	Female	53	1441.7	2.7
PDAC242	PDAC	CHCAMS	Female	38	1457.2	5.5
PDAC243	PDAC	CHCAMS	Male	63	1506.2	2.3
PDAC244	PDAC	CHCAMS	Female	69	1544.2	7.6
PDAC245	PDAC	CHCAMS	Female	26	1537.3	2.8
PDAC246	PDAC	CHCAMS	Female	32	1546.1	1.1
PDAC247	PDAC	CHCAMS	Male	28	1557	2.4
PDAC248	PDAC	CHCAMS	Male	53	1577.3	1.1
PDAC249	PDAC	CHCAMS	Female	38	1641.2	3.8
PDAC250	PDAC	CHCAMS	Male	54	1679.6	1.0
PDAC251	PDAC	CHCAMS	Female	47	1700.3	3.2
PDAC252	PDAC	CHCAMS	Female	53	1730.2	2.2
PDAC253	PDAC	CHCAMS	Female	63	1742.6	3.5
PDAC254	PDAC	CHCAMS	Female	47	1777	1.6
PDAC255	PDAC	CHCAMS	Male	68	1854.9	2.3
PDAC256	PDAC	CHCAMS	Female	28	2176	1.6
HCC1	HCC	CHCAMS	Male	29	80.4	1.2
HCC2	HCC	CHCAMS	Male	36	80.6	0.9
HCC3	HCC	CHCAMS	Female	57	88.1	3.3
HCC4	HCC	CHCAMS	Female	43	93.9	1.3
HCC5	HCC	CHCAMS	Male	66	122.1	8.1
HCC6	HCC	CHCAMS	Male	65	138.6	5.2
HCC7	HCC	CHCAMS	Male	52	137.3	0.9
HCC8	HCC	CHCAMS	Male	63	213	2.5
HCC9	HCC	CHCAMS	Female	72	221	1.4
HCC10	HCC	CHCAMS	Male	66	226.7	1.1
HCC11	HCC	CHCAMS	Male	74	255.4	1.2
HCC12	HCC	CHCAMS	Male	38	307.6	5.3
HCC13	HCC	CHCAMS	Male	73	317.7	2.5
HCC14	HCC	CHCAMS	Female	45	328.4	6.9
HCC15	HCC	CHCAMS	Male	63	324.4	1.7
HCC16	HCC	CHCAMS	Male	34	328.1	1.1
HCC17	HCC	CHCAMS	Male	25	329.3	4.0
HCC18	HCC	CHCAMS	Male	60	332.5	1.4
HCC19	HCC	CHCAMS	Male	66	351.3	2.2
HCC20	HCC	CHCAMS	Male	62	366	3.9
HCC21	HCC	CHCAMS	Male	25	380	1.2
HCC22	HCC	CHCAMS	Female	48	388	1.7

HCC23	HCC	CHCAMS	Male	47	392.5	2.6
HCC24	HCC	CHCAMS	Male	66	399.8	1.8
HCC25	HCC	CHCAMS	Female	47	426.6	1.2
HCC26	HCC	CHCAMS	Male	26	431.4	5.4
HCC27	HCC	CHCAMS	Female	31	431.3	3.8
HCC28	HCC	CHCAMS	Male	55	452.8	2.1
HCC29	HCC	CHCAMS	Female	70	452.5	2.4
HCC30	HCC	CHCAMS	Female	61	458	3.4
HCC31	HCC	CHCAMS	Female	73	467.8	1.3
HCC32	HCC	CHCAMS	Male	30	482.3	2.3
HCC33	HCC	CHCAMS	Male	52	477.7	0.7
HCC34	HCC	CHCAMS	Male	40	476.5	4.3
HCC35	HCC	CHCAMS	Female	26	480.2	1.4
HCC36	HCC	CHCAMS	Male	61	492	7.9
HCC37	HCC	CHCAMS	Female	44	481.4	2.8
HCC38	HCC	CHCAMS	Male	63	488.2	0.5
HCC39	HCC	CHCAMS	Female	27	491.5	2.7
HCC40	HCC	CHCAMS	Female	52	510.8	5.0
HCC41	HCC	CHCAMS	Female	73	515.6	1.2
HCC42	HCC	CHCAMS	Male	27	521.4	0.6
HCC43	HCC	CHCAMS	Male	64	527.9	1.2
HCC44	HCC	CHCAMS	Male	74	533.9	1.5
HCC45	HCC	CHCAMS	Female	46	551.4	5.6
HCC46	HCC	CHCAMS	Male	39	546.4	1.5
HCC47	HCC	CHCAMS	Female	70	566.6	4.4
HCC48	HCC	CHCAMS	Male	33	565.2	2.1
HCC49	HCC	CHCAMS	Male	35	575.2	1.2
HCC50	HCC	CHCAMS	Male	49	570.7	2.8
HCC51	HCC	CHCAMS	Male	35	580.8	1.4
HCC52	HCC	CHCAMS	Male	30	591.8	2.0
HCC53	HCC	CHCAMS	Female	43	591	1.6
HCC54	HCC	CHCAMS	Female	32	596.4	0.6
HCC55	HCC	CHCAMS	Female	51	618.1	1.3
HCC56	HCC	CHCAMS	Female	57	623.7	2.7
HCC57	HCC	CHCAMS	Female	41	625.3	3.5
HCC58	HCC	CHCAMS	Male	66	632.7	1.4
HCC59	HCC	CHCAMS	Male	73	638.3	1.8
HCC60	HCC	CHCAMS	Male	50	638.6	2.2
HCC61	HCC	CHCAMS	Male	26	644.1	4.5
HCC62	HCC	CHCAMS	Male	57	637.2	2.5
HCC63	HCC	CHCAMS	Male	68	644.5	1.4
HCC64	HCC	CHCAMS	Female	43	642.8	2.9
HCC65	HCC	CHCAMS	Female	28	664.8	1.8
HCC66	HCC	CHCAMS	Male	54	681.4	2.4

HCC67	HCC	CHCAMS	Female	34	679	2.3
HCC68	HCC	CHCAMS	Female	59	688.1	2.7
HCC69	HCC	CHCAMS	Female	71	685.4	2.2
HCC70	HCC	CHCAMS	Female	42	684.5	6.3
HCC71	HCC	CHCAMS	Female	28	697.5	1.4
HCC72	HCC	CHCAMS	Male	49	701.9	1.5
HCC73	HCC	CHCAMS	Female	57	713.6	2.7
HCC74	HCC	CHCAMS	Female	36	708.3	1.6
HCC75	HCC	CHCAMS	Male	62	717.9	1.0
HCC76	HCC	CHCAMS	Female	61	734	6.2
HCC77	HCC	CHCAMS	Female	40	732.2	6.4
HCC78	HCC	CHCAMS	Female	65	735.8	2.0
HCC79	HCC	CHCAMS	Male	35	740.1	2.6
HCC80	HCC	CHCAMS	Female	33	747.6	0.5
HCC81	HCC	CHCAMS	Male	33	744.8	1.3
HCC82	HCC	CHCAMS	Male	31	743.2	2.7
HCC83	HCC	CHCAMS	Female	69	757	3.3
HCC84	HCC	CHCAMS	Female	28	759.1	2.2
HCC85	HCC	CHCAMS	Female	71	763.3	1.6
HCC86	HCC	CHCAMS	Female	71	765.8	2.1
HCC87	HCC	CHCAMS	Male	29	769.3	3.2
HCC88	HCC	CHCAMS	Female	45	765.3	1.8
HCC89	HCC	CHCAMS	Male	32	763.3	1.0
HCC90	HCC	CHCAMS	Female	29	771.8	2.8
HCC91	HCC	CHCAMS	Female	73	784.5	3.8
HCC92	HCC	CHCAMS	Female	48	781.6	2.6
HCC93	HCC	CHCAMS	Male	34	800.4	5.9
HCC94	HCC	CHCAMS	Male	70	802.2	4.0
HCC95	HCC	CHCAMS	Male	56	790	0.6
HCC96	HCC	CHCAMS	Female	27	798.2	3.6
HCC97	HCC	CHCAMS	Female	38	792.6	3.2
HCC98	HCC	CHCAMS	Female	41	811.1	0.9
HCC99	HCC	CHCAMS	Female	65	819.8	1.8
HCC100	HCC	CHCAMS	Female	42	816.2	2.8
HCC101	HCC	CHCAMS	Female	55	827.2	0.5
HCC102	HCC	CHCAMS	Female	28	834.3	1.8
HCC103	HCC	CHCAMS	Female	54	833.2	0.6
HCC104	HCC	CHCAMS	Female	42	839.5	2.8
HCC105	HCC	CHCAMS	Male	68	835.9	0.9
HCC106	HCC	CHCAMS	Female	53	837.4	4.1
HCC107	HCC	CHCAMS	Female	72	845	2.6
HCC108	HCC	CHCAMS	Female	30	851.4	3.0
HCC109	HCC	CHCAMS	Male	29	850.9	2.2
HCC110	HCC	CHCAMS	Male	41	853.2	4.7

HCC111	HCC	CHCAMS	Male	28	849.9	1.0
HCC112	HCC	CHCAMS	Female	58	853.7	1.7
HCC113	HCC	CHCAMS	Male	49	856.1	3.1
HCC114	HCC	CHCAMS	Female	63	862.6	3.4
HCC115	HCC	CHCAMS	Female	25	864.9	2.9
HCC116	HCC	CHCAMS	Male	65	870	0.4
HCC117	HCC	CHCAMS	Male	27	874.2	0.9
HCC118	HCC	CHCAMS	Female	56	865.8	3.5
HCC119	HCC	CHCAMS	Female	62	879.3	5.0
HCC120	HCC	CHCAMS	Female	37	870.3	2.9
HCC121	HCC	CHCAMS	Female	38	875.4	1.3
HCC122	HCC	CHCAMS	Male	29	877.5	1.2
HCC123	HCC	CHCAMS	Female	43	879.2	1.3
HCC124	HCC	CHCAMS	Male	43	879.2	2.5
HCC125	HCC	CHCAMS	Female	37	884.8	1.5
HCC126	HCC	CHCAMS	Female	72	887.6	3.9
HCC127	HCC	CHCAMS	Female	72	896.9	6.6
HCC128	HCC	CHCAMS	Male	48	886.4	3.5
HCC129	HCC	CHCAMS	Male	44	892.3	3.0
HCC130	HCC	CHCAMS	Male	49	894.8	4.3
HCC131	HCC	CHCAMS	Male	42	906.4	1.8
HCC132	HCC	CHCAMS	Female	74	904.7	2.2
HCC133	HCC	CHCAMS	Male	69	904.9	2.6
HCC134	HCC	CHCAMS	Male	37	908.7	2.0
HCC135	HCC	CHCAMS	Male	74	930.1	2.2
HCC136	HCC	CHCAMS	Male	43	923.3	1.5
HCC137	HCC	CHCAMS	Female	26	933.1	2.3
HCC138	HCC	CHCAMS	Male	61	929	4.0
HCC139	HCC	CHCAMS	Male	27	950.9	3.5
HCC140	HCC	CHCAMS	Female	27	943.7	1.8
HCC141	HCC	CHCAMS	Female	55	956.5	2.8
HCC142	HCC	CHCAMS	Female	59	960	1.0
HCC143	HCC	CHCAMS	Male	31	968.2	2.8
HCC144	HCC	CHCAMS	Female	54	963.8	1.0
HCC145	HCC	CHCAMS	Female	51	964.4	1.7
HCC146	HCC	CHCAMS	Female	32	974.9	0.4
HCC147	HCC	CHCAMS	Male	46	986.9	1.7
HCC148	HCC	CHCAMS	Female	65	989.6	2.7
HCC149	HCC	CHCAMS	Female	39	999.1	9.3
HCC150	HCC	CHCAMS	Female	54	984	2.0
HCC151	HCC	CHCAMS	Female	62	994.3	1.3
HCC152	HCC	CHCAMS	Male	29	1003.6	1.6
HCC153	HCC	CHCAMS	Female	53	998.5	1.0
HCC154	HCC	CHCAMS	Male	43	1001.5	1.7

HCC155	HCC	CHCAMS	Male	45	1021.6	1.0
HCC156	HCC	CHCAMS	Male	55	1019	3.2
HCC157	HCC	CHCAMS	Female	30	1018.6	2.0
HCC158	HCC	CHCAMS	Male	60	1026.1	1.7
HCC159	HCC	CHCAMS	Female	38	1026	2.2
HCC160	HCC	CHCAMS	Female	43	1038	1.9
HCC161	HCC	CHCAMS	Female	71	1053.7	1.1
HCC162	HCC	CHCAMS	Female	42	1061.6	2.0
HCC163	HCC	CHCAMS	Male	34	1057.8	2.2
HCC164	HCC	CHCAMS	Male	60	1056.9	1.3
HCC165	HCC	CHCAMS	Male	52	1066.5	1.7
HCC166	HCC	CHCAMS	Male	42	1074.1	1.0
HCC167	HCC	CHCAMS	Female	48	1080.8	2.1
HCC168	HCC	CHCAMS	Male	74	1077.7	2.7
HCC169	HCC	CHCAMS	Male	30	1084.4	0.6
HCC170	HCC	CHCAMS	Male	53	1111.1	1.3
HCC171	HCC	CHCAMS	Female	46	1111.4	6.5
HCC172	HCC	CHCAMS	Female	44	1127.1	2.2
HCC173	HCC	CHCAMS	Male	44	1124.8	3.0
HCC174	HCC	CHCAMS	Male	44	1133.2	2.7
HCC175	HCC	CHCAMS	Male	27	1134.2	2.1
HCC176	HCC	CHCAMS	Male	66	1146.3	4.9
HCC177	HCC	CHCAMS	Male	56	1140.6	2.2
HCC178	HCC	CHCAMS	Female	68	1148.1	1.8
HCC179	HCC	CHCAMS	Male	26	1148.3	0.8
HCC180	HCC	CHCAMS	Female	28	1153.1	2.8
HCC181	HCC	CHCAMS	Male	68	1151.4	1.1
HCC182	HCC	CHCAMS	Male	65	1163.7	2.1
HCC183	HCC	CHCAMS	Female	57	1166.3	0.3
HCC184	HCC	CHCAMS	Female	63	1181.6	3.5
HCC185	HCC	CHCAMS	Male	37	1177.1	2.0
HCC186	HCC	CHCAMS	Female	73	1181.8	4.0
HCC187	HCC	CHCAMS	Female	29	1185.3	2.9
HCC188	HCC	CHCAMS	Male	41	1186.3	1.0
HCC189	HCC	CHCAMS	Male	45	1188.7	3.8
HCC190	HCC	CHCAMS	Female	51	1193.7	2.4
HCC191	HCC	CHCAMS	Female	47	1204	1.3
HCC192	HCC	CHCAMS	Female	47	1197.3	2.0
HCC193	HCC	CHCAMS	Female	59	1199.3	2.8
HCC194	HCC	CHCAMS	Female	66	1220.3	4.4
HCC195	HCC	CHCAMS	Female	38	1226	2.1
HCC196	HCC	CHCAMS	Male	48	1231	3.4
HCC197	HCC	CHCAMS	Female	58	1239	2.2
HCC198	HCC	CHCAMS	Male	58	1248.8	1.5

HCC199	HCC	CHCAMS	Male	37	1257.2	1.7
HCC200	HCC	CHCAMS	Female	34	1258.2	1.2
HCC201	HCC	CHCAMS	Female	40	1273.5	5.9
HCC202	HCC	CHCAMS	Male	55	1258.8	5.1
HCC203	HCC	CHCAMS	Male	52	1106.0	0.2
HCC204	HCC	CHCAMS	Female	60	1007.2	0.4
HCC205	HCC	CHCAMS	Female	33	1186.7	6.4
HCC206	HCC	CHCAMS	Female	37	1269.9	0.8
HCC207	HCC	CHCAMS	Female	38	1226.6	2.4
HCC208	HCC	CHCAMS	Male	32	1161.8	1.7
HCC209	HCC	CHCAMS	Male	59	1155.2	0.5
HCC210	HCC	CHCAMS	Male	44	1164.5	1.2
HCC211	HCC	CHCAMS	Female	37	1265.5	1.2
HCC212	HCC	CHCAMS	Male	37	1288.6	1.0
HCC213	HCC	CHCAMS	Female	60	1297.8	5.8
HCC214	HCC	CHCAMS	Male	59	1140.7	1.8
HCC215	HCC	CHCAMS	Male	42	1163.6	2.5
HCC216	HCC	CHCAMS	Female	54	1267.0	3.8
HCC217	HCC	CHCAMS	Male	56	1102.6	2.2
HCC218	HCC	CHCAMS	Male	69	1077.1	4.6
HCC219	HCC	CHCAMS	Male	56	1198.1	1.8
HCC220	HCC	CHCAMS	Female	34	1133.7	3.6
HCC221	HCC	CHCAMS	Male	51	1224.8	2.0
HCC222	HCC	CHCAMS	Female	68	1334.4	5.8
HCC223	HCC	CHCAMS	Female	45	1341.6	3.1
HCC224	HCC	CHCAMS	Male	32	1339.6	1.9
HCC225	HCC	CHCAMS	Male	55	1343.6	4.3
HCC226	HCC	CHCAMS	Male	55	1364.1	1.6
HCC227	HCC	CHCAMS	Female	57	1363.6	5.2
HCC228	HCC	CHCAMS	Female	46	1368.1	2.9
HCC229	HCC	CHCAMS	Female	33	1367.6	1.3
HCC230	HCC	CHCAMS	Male	59	1378	2.2
HCC231	HCC	CHCAMS	Male	72	1371.7	2.2
HCC232	HCC	CHCAMS	Female	26	1302.4	6.4
HCC233	HCC	CHCAMS	Female	50	1370	2.9
HCC234	HCC	CHCAMS	Female	34	1384.1	3.8
HCC235	HCC	CHCAMS	Male	53	1218.2	1.5
HCC236	HCC	CHCAMS	Male	34	1392	4.4
HCC237	HCC	CHCAMS	Male	33	1472.6	2.6
HCC238	HCC	CHCAMS	Female	37	1255.1	1.1
HCC239	HCC	CHCAMS	Female	57	1255.6	1.1
HCC240	HCC	CHCAMS	Female	34	1325.3	4.5
HCC241	HCC	CHCAMS	Female	59	1172	3.6
HCC242	HCC	CHCAMS	Male	67	1335.1	3.8

HCC243	HCC	CHCAMS	Female	26	1172.5	6.3
HCC244	HCC	CHCAMS	Male	70	1361.9	1.5
HCC245	HCC	CHCAMS	Female	51	1303.5	1.3
HCC246	HCC	CHCAMS	Female	45	1316.8	2.9
HCC247	HCC	CHCAMS	Male	40	1263.3	0.8
HCC248	HCC	CHCAMS	Male	49	1182.3	2.1
HCC249	HCC	CHCAMS	Male	70	1282.9	8.9
HCC250	HCC	CHCAMS	Female	73	1289.3	1.4
HCC251	HCC	CHCAMS	Male	72	1558	2.2
HCC252	HCC	CHCAMS	Female	68	1458.7	1.4
HCC253	HCC	CHCAMS	Male	46	1592.4	4.4
HCC254	HCC	CHCAMS	Female	35	1588.1	3.9
HCC255	HCC	CHCAMS	Female	59	1660.7	2.9
HCC256	HCC	CHCAMS	Female	57	1682.9	0.8
HCC257	HCC	CHCAMS	Male	42	1677	2.1
HCC258	HCC	CHCAMS	Male	41	1831	5.1
HCC259	HCC	CHCAMS	Female	48	1795.8	2.3
HCC260	HCC	CHCAMS	Female	60	1802.3	2.8
HCC261	HCC	CHCAMS	Female	36	1769.6	1.4
HCC262	HCC	CHCAMS	Female	32	1883.6	2.7
HCC263	HCC	CHCAMS	Male	31	1932.1	3.9
HCC264	HCC	CHCAMS	Male	56	2028.1	0.5
HIN1	Colorectal HIN	SYSUCC	Male	64	952.8	6.1
HIN2	Colorectal HIN	SYSUCC	Female	69	781.4	5.3
HIN3	Colorectal HIN	SYSUCC	Female	34	1104.4	11.2
HIN4	Colorectal HIN	SYSUCC	Male	62	1319.3	27.4
HIN5	Colorectal HIN	SYSUCC	Male	69	1433.1	35.5
HIN6	Colorectal HIN	SYSUCC	Male	55	991.3	9.7
HIN7	Colorectal HIN	SYSUCC	Male	54	1003.2	6.9
HIN8	Colorectal HIN	SYSUCC	Male	30	1130	17.7
HIN9	Colorectal HIN	SYSUCC	Male	27	1040.5	9.0
HIN10	Colorectal HIN	SYSUCC	Male	55	1030.4	12.6
HIN11	Colorectal HIN	SYSUCC	Male	36	1212.4	20.4
HIN12	Colorectal HIN	SYSUCC	Male	27	1270.8	17.0
HIN13	Colorectal HIN	SYSUCC	Female	50	1176.2	14.2
HIN14	Colorectal HIN	SYSUCC	Female	67	1384.1	20.5
HIN15	Colorectal HIN	SYSUCC	Male	55	1301.3	13.6
HIN16	Colorectal HIN	SYSUCC	Female	46	1474	25.1
HIN17	Colorectal HIN	SYSUCC	Female	59	1323.7	12.8
HIN18	Colorectal HIN	SYSUCC	Female	63	1505.4	22.0
HIN19	Colorectal HIN	SYSUCC	Female	41	1371.9	11.0
HIN20	Colorectal HIN	SYSUCC	Male	53	1426.4	1.8
HIN21	Colorectal HIN	SYSUCC	Male	59	1504.8	17.9
HIN22	Colorectal HIN	SYSUCC	Female	47	1381.7	10.9

HIN23	Colorectal HIN	SYSUCC	Male	36	1337.6	13.3
HIN24	Colorectal HIN	SYSUCC	Male	42	1519.6	21.4
HIN25	Colorectal HIN	SYSUCC	Male	31	1542.2	5.9
HIN26	Colorectal HIN	SYSUCC	Female	48	1725.6	24.6
HIN27	Colorectal HIN	SYSUCC	Female	28	2022.9	35.5
HIN28	Colorectal HIN	SYSUCC	Male	31	1649.3	28.2
HIN29	Colorectal HIN	SYSUCC	Male	60	1397.2	10.4
HIN30	Colorectal HIN	SYSUCC	Female	49	1775.2	35.2
HIN31	Colorectal HIN	SYSUCC	Female	66	1496.8	7.1
HIN32	Colorectal HIN	SYSUCC	Female	30	1940.4	3.0
HIN33	Colorectal HIN	SYSUCC	Female	35	1738.1	26.4
HIN34	Colorectal HIN	SYSUCC	Male	58	1465.2	9.6
HIN35	Colorectal HIN	SYSUCC	Female	44	1509.7	6.4
HIN36	Colorectal HIN	SYSUCC	Female	67	1457.3	9.5
HIN37	Colorectal HIN	SYSUCC	Male	33	1544.6	12.7
HIN38	Colorectal HIN	SYSUCC	Male	52	1729.7	15.4
HIN39	Colorectal HIN	SYSUCC	Male	68	1577.5	19.1
HIN40	Colorectal HIN	SYSUCC	Male	45	1520.9	6.3
HIN41	Colorectal HIN	SYSUCC	Female	73	1676.6	8.3
HIN42	Colorectal HIN	SYSUCC	Male	34	1833.2	23.3
HIN43	Colorectal HIN	SYSUCC	Male	70	1935.2	27.5
HIN44	Colorectal HIN	SYSUCC	Male	33	1561.8	5.7
HIN45	Colorectal HIN	SYSUCC	Male	39	1504.1	13.3
HIN46	Colorectal HIN	SYSUCC	Female	69	1520.6	5.5
HIN47	Colorectal HIN	SYSUCC	Male	33	1523.7	4.9
HIN48	Colorectal HIN	SYSUCC	Female	56	1568.2	6.0
HIN49	Colorectal HIN	SYSUCC	Male	34	1737.2	17.1
HIN50	Colorectal HIN	SYSUCC	Male	64	1884.4	24.8
HIN51	Colorectal HIN	SYSUCC	Male	59	1642.1	8.6
HIN52	Colorectal HIN	SYSUCC	Male	47	1768.1	14.8
HIN53	Colorectal HIN	SYSUCC	Male	40	1560.6	6.8
HIN54	Colorectal HIN	SYSUCC	Female	36	1687.4	12.9
HIN55	Colorectal HIN	SYSUCC	Female	33	1642.2	17.9
HIN56	Colorectal HIN	SYSUCC	Female	62	1665.3	16.8
HIN57	Colorectal HIN	SYSUCC	Male	75	1756	6.4
HIN58	Colorectal HIN	SYSUCC	Female	70	1772.3	7.6
HIN59	Colorectal HIN	SYSUCC	Female	26	1781.4	16.0
HIN60	Colorectal HIN	SYSUCC	Male	43	1653.4	11.4
HIN61	Colorectal HIN	SYSUCC	Male	31	1908.8	34.9
HIN62	Colorectal HIN	SYSUCC	Female	48	1796.2	13.9
HIN63	Colorectal HIN	SYSUCC	Male	71	1801.7	9.2
HIN64	Colorectal HIN	SYSUCC	Male	68	1812.6	6.9
HIN65	Colorectal HIN	SYSUCC	Female	60	1923.8	6.2
HIN66	Colorectal HIN	SYSUCC	Male	69	2036.9	14.0

HIN67	Colorectal HIN	SYSUCC	Female	67	2098.9	26.5
HIN68	Colorectal HIN	SYSUCC	Female	57	2374.9	10.1
HIN69	Colorectal HIN	SYSUCC	Male	51	2933.3	7.6
HIN70	Colorectal HIN	SYSUCC	Female	73	2040.6	8.4
HIN71	Colorectal HIN	SYSUCC	Male	44	2061.5	11.1
HIN72	Colorectal HIN	SYSUCC	Male	73	1362.5	4.2
HIN73	Colorectal HIN	SYSUCC	Female	63	1883.9	14.6
HIN74	Colorectal HIN	SYSUCC	Male	53	1639.1	21.1
HIN75	Colorectal HIN	SYSUCC	Male	53	1422.6	1.7
HIN76	Colorectal HIN	SYSUCC	Male	70	2338.4	30.7
HIN77	Colorectal HIN	SYSUCC	Female	61	1789.6	16.7
HIN78	Colorectal HIN	SYSUCC	Female	69	1748.9	13.4
HIN79	Colorectal HIN	SYSUCC	Male	39	1970.7	25.6
HIN80	Colorectal HIN	SYSUCC	Male	45	1627.2	9.0
HIN81	Colorectal HIN	SYSUCC	Female	63	1572.8	19.0
CP1	CP	SYSUCC	Male	75	1114.7	4.0
CP2	CP	SYSUCC	Female	43	1363.5	1.0
CP3	CP	SYSUCC	Male	49	978.6	8.0
CP4	CP	SYSUCC	Female	72	1456.7	9.0
CP5	CP	SYSUCC	Male	34	1081	4.2
CP6	CP	SYSUCC	Male	25	1134	4.8
CP7	CP	SYSUCC	Male	29	994.3	4.4
CP8	CP	SYSUCC	Male	68	1553.9	3.8
CP9	CP	SYSUCC	Male	59	1132.4	14.0
CP10	CP	SYSUCC	Male	33	1276.9	14.7
CP11	CP	SYSUCC	Female	38	1129.1	3.8
CP12	CP	SYSUCC	Female	50	1224.2	9.4
CP13	CP	SYSUCC	Female	25	1334.9	7.6
CP14	CP	SYSUCC	Male	37	1206	5.0
CP15	CP	SYSUCC	Male	39	1484.8	24.4
CP16	CP	SYSUCC	Female	40	1527	11.0
CP17	CP	SYSUCC	Female	41	1625.4	12.5
CP18	CP	SYSUCC	Female	66	1412.5	1.1
CP19	CP	SYSUCC	Female	69	1462.5	3.1
CP20	CP	SYSUCC	Male	53	1467.2	1.9
CP21	CP	SYSUCC	Female	61	1655	4.8
CP22	CP	SYSUCC	Male	47	1852.7	17.9
CP23	CP	SYSUCC	Female	28	912.3	10.6
CP24	CP	SYSUCC	Female	26	951.1	8.7
CP25	CP	SYSUCC	Female	51	1192.5	13.4
CP26	CP	SYSUCC	Female	49	1183.8	7.6
CP27	CP	SYSUCC	Male	70	1063.7	2.3
CP28	CP	SYSUCC	Female	75	1318.1	4.1
CP29	CP	SYSUCC	Male	54	1175.1	8.5

CP30	CP	SYSUCC	Female	26	1339.3	12.6
CP31	CP	SYSUCC	Male	42	1352.5	14.2
CP32	CP	SYSUCC	Female	62	1370.8	11.0
CP33	CP	SYSUCC	Male	52	1375.8	2.1
CP34	CP	SYSUCC	Male	71	1515	10.3
CP35	CP	SYSUCC	Female	60	1227.7	9.3
CP36	CP	SYSUCC	Female	29	1306.8	9.4
CP37	CP	SYSUCC	Female	50	1038.9	3.9
CP38	CP	SYSUCC	Male	25	630.7	5.8
CP39	CP	SYSUCC	Female	26	1127.3	7.2
CP40	CP	SYSUCC	Male	59	1193.8	11.4
CP41	CP	SYSUCC	Female	53	818.3	16.2
CP42	CP	SYSUCC	Female	27	1526.6	14.2
CP43	CP	SYSUCC	Female	40	1261	9.4
CP44	CP	SYSUCC	Male	25	796	11.5
CP45	CP	SYSUCC	Male	34	1193.6	18.8
CP46	CP	SYSUCC	Female	27	891.8	22.0
CP47	CP	SYSUCC	Male	48	747.8	11.0
CP48	CP	SYSUCC	Male	63	906	5.4
CP49	CP	SYSUCC	Male	73	1470.2	10.4
CP50	CP	SYSUCC	Male	38	924.2	5.6
CP51	CP	SYSUCC	Male	31	968.9	2.4
CP52	CP	SYSUCC	Male	64	768.1	9.8
CP53	CP	SYSUCC	Male	72	1017.2	11.4
CP54	CP	SYSUCC	Female	37	918.6	8.7
CP55	CP	SYSUCC	Male	51	1077.3	6.7
CP56	CP	SYSUCC	Female	46	1264.3	8.9
CP57	CP	SYSUCC	Female	49	522.6	8.2
CP58	CP	SYSUCC	Male	35	1148.7	18.4
CP59	CP	SYSUCC	Male	69	1546.1	14.3
CP60	CP	SYSUCC	Male	52	748.2	15.1
CP61	CP	SYSUCC	Female	29	1362	3.7
CP62	CP	SYSUCC	Female	39	1466.6	14.9
CP63	CP	SYSUCC	Female	39	1263	10.6
CP64	CP	SYSUCC	Male	35	1315.2	9.6
CP65	CP	SYSUCC	Female	39	1051.7	7.3
CP66	CP	SYSUCC	Female	31	829.5	12.1
CP67	CP	SYSUCC	Male	60	789.6	0.7
CP68	CP	SYSUCC	Female	26	980.5	21.0
CP69	CP	SYSUCC	Female	70	1093.5	14.0
CP70	CP	SYSUCC	Female	49	811.4	25.7
CP71	CP	SYSUCC	Male	59	633	8.3
CP72	CP	SYSUCC	Female	43	792.1	13.8
CP73	CP	SYSUCC	Female	44	1495.7	7.9

CP74	CP	SYSUCC	Female	31	1440.5	5.2
CP75	CP	SYSUCC	Male	69	1236.6	11.1
CP76	CP	SYSUCC	Male	27	1420.4	6.5
CP77	CP	SYSUCC	Male	69	1395.5	7.7
CP78	CP	SYSUCC	Male	60	1559.1	4.8
CP79	CP	SYSUCC	Female	26	1212.4	11.6
CP80	CP	SYSUCC	Male	67	640.3	9.3
CP81	CP	SYSUCC	Female	28	1184	14.2
CP82	CP	SYSUCC	Female	52	1162.7	13.7
CP83	CP	SYSUCC	Female	57	1143.5	11.8
CP84	CP	SYSUCC	Male	61	1373.2	7.1
CP85	CP	SYSUCC	Female	49	1541.8	15.2
CP86	CP	SYSUCC	Male	60	1654.6	14.2
CP87	CP	SYSUCC	Female	30	751.9	21.8
CP88	CP	SYSUCC	Male	28	1067.6	4.6
CP89	CP	SYSUCC	Male	45	745.4	4.5
CP90	CP	SYSUCC	Male	75	1301.2	4.5
CP91	CP	SYSUCC	Female	54	497.3	5.5
CP92	CP	SYSUCC	Female	62	955.8	14.8
CP93	CP	SYSUCC	Male	49	1207.7	18.4
CP94	CP	SYSUCC	Male	26	1082.8	7.9
CP95	CP	SYSUCC	Female	52	1284.5	5.2
CP96	CP	SYSUCC	Male	42	1280.9	5.6
CP97	CP	SYSUCC	Female	72	801.6	5.6
CP98	CP	SYSUCC	Female	34	1333.1	10.9
CP99	CP	SYSUCC	Male	54	893.3	8.6
CP100	CP	SYSUCC	Female	45	1391.6	1.9
CP101	CP	SYSUCC	Male	73	1362.6	8.7
CP102	CP	SYSUCC	Male	62	863	0.5
CP103	CP	SYSUCC	Male	57	894.5	8.4
CP104	CP	SYSUCC	Female	37	914.5	5.5
CP105	CP	SYSUCC	Male	65	831.3	16.8
CP106	CP	SYSUCC	Male	65	705.3	6.7
CP107	CP	SYSUCC	Female	55	1005.8	9.2
CP108	CP	SYSUCC	Female	52	1276.4	5.7
CP109	CP	SYSUCC	Male	72	1606.6	24.1
CP110	CP	SYSUCC	Male	63	1088.2	2.5
CP111	CP	SYSUCC	Female	27	795.5	7.6

[1]ESCC, esophageal squamous-cell carcinoma; GCC, gastric cardia carcinoma; GC, gastric carcinoma; PDAC, pancreatic ductal adenocarcinoma; HCC, hepatocellular carcinoma; Colorectal HIN, high-grade intraepithelial neoplasia of colorectum; CP, colorectal polyps.
[2]SYSUCC, Sun Yat-sen University Cancer Center (Guangzhou, China); CHCAMS, Cancer Hospital of Chinese Academy of Medical Sciences (Beijing, China).
[3]Mean of triplicate measurements and the standard error of mean (SEM). Serum piR-54265 level was determined from blood of individuals at diagnosis prior to any treatments.

Table S2. Demographic and clinical characteristics and serum piR-54265 level of patients with CRC

Sample ID	Patient[1]	Sex	Age (year)	CRC location	TNM stage	Serum piR-54265[2]			
						Before surgery		After surgery	
						Mean copy/μL	SEM	Mean copy/μL	SEM
Pre-1	SYSUCC	Male	39	Colon	IV	4342.2	3.9	2490.3	6.3
Pre-2	SYSUCC	Female	42	Rectum	III	3360.3	6.5	2283.4	6.3
Pre-3	SYSUCC	Male	61	Colon	I	3134.2	10.4	1465.6	5.1
Pre-4	SYSUCC	Male	59	Rectum	II	2252.6	13.2	927.4	4.1
Pre-5	SYSUCC	Female	58	Rectum	II	1757.8	10.2	1051.0	7.5
Pre-6	SYSUCC	Male	49	Colon	II	6922.2	19.3	1599.5	6.0
Pre-7	SYSUCC	Female	72	Colon	II	3431.9	15.5	780.6	7.0
Pre-8	SYSUCC	Male	72	Rectum	II	2960.2	1.6	1489.0	5.6
Pre-9	SYSUCC	Female	60	Rectum	II	2607.8	13.8	1225.6	4.4
Pre-10	SYSUCC	Female	73	Colon	II	2657.6	3.4	1532.1	5.0
Pre-11	SYSUCC	Female	45	Rectum	IV	4010.4	6.0	2288.9	5.6
Pre-12	SYSUCC	Male	69	Rectum	III	3013.7	13.7	2420.8	7.0
Pre-13	SYSUCC	Male	47	Colon	II	2272.9	10.5	1088.1	4.4
Pre-14	SYSUCC	Female	78	Rectum	II	2742.9	10.9	1055.3	3.9
Pre-15	SYSUCC	Male	60	Rectum	II	2056.3	6.4	662.7	6.9
Pre-16	SYSUCC	Male	59	Rectum	II	3070.1	19.9	1205.2	9.5
Pre-17	SYSUCC	Male	72	Colon	III	3890.9	11.5	1221.0	7.4
Pre-18	SYSUCC	Male	75	Colon	II	4085.0	7.4	1341.0	9.6
Pre-19	SYSUCC	Female	56	Rectum	III	1834.3	17.2	1147.8	6.3
Pre-20	SYSUCC	Male	73	Colon	I	1794.5	3.9	1404.8	6.7
Pre-21	SYSUCC	Male	74	Colon	I	2581.8	4.6	1647.4	4.5
Pre-22	SYSUCC	Female	72	Rectum	II	1638.3	3.9	764.5	10.5
Pre-23	SYSUCC	Male	72	Rectum	III	2014.4	7.7	1076.5	11.4
Pre-24	SYSUCC	Female	60	Rectum	I	1726.3	6.9	844.4	4.0
Pre-25	SYSUCC	Female	73	Colon	II	2480.7	5.1	1480.8	5.1
Pre-26	SYSUCC	Female	45	Colon	II	3536.6	6.3	2370.9	1.1
Pre-27	SYSUCC	Male	69	Rectum	II	1565.1	5.3	733.0	7.9
Pre-28	SYSUCC	Male	47	Colon	I	3089.1	6.1	1571.2	5.2
Pre-29	SYSUCC	Female	78	Rectum	III	2326.5	5.7	1385.5	6.5
Pre-30	SYSUCC	Male	60	Rectum	II	2273.7	1.4	1186.4	6.1
Pre-31	SYSUCC	Male	59	Colon	II	2204.4	7.5	1237.3	10.1
Pre-32	SYSUCC	Male	72	Colon	II	3123.4	4.7	2040.1	3.2
Pre-33	SYSUCC	Male	75	Colon	I	2105.3	5.5	1685.8	38.5
Pre-34	SYSUCC	Male	72	Rectum	II	2326.1	5.6	1968.0	11.6
Pre-35	SYSUCC	Male	58	Rectum	II	2087.1	5.1	1310.1	3.7
Pre-36	SYSUCC	Male	72	Colon	III	3069.1	7.2	2377.4	1.4
Pre-37	SYSUCC	Male	66	Colon	IV	3418.1	7.3	1382.8	6.2
Pre-38	SYSUCC	Female	65	Rectum	I	2633.0	5.8	795.7	1.2

Pre-39	SYSUCC	Female	52	Rectum	I	2257.1	3.3	1405.9	6.8
Pre-40	SYSUCC	Male	33	Colon	II	2915.8	5.2	1876.5	9.3
Pre-41	SYSUCC	Female	34	Colon	II	2628.8	2.8	1342.2	0.7
Pre-42	SYSUCC	Female	74	Rectum	I	3433.2	3.4	2400.2	19.0
Pre-43	SYSUCC	Female	58	Colon	II	1701.6	17.5	1362.7	7.8
Pre-44	SYSUCC	Male	49	Colon	I	2270.7	5.2	1682.7	5.1
Pre-45	SYSUCC	Male	44	Colon	III	4826.1	6.4	2528.8	7.3
Pre-46	SYSUCC	Male	77	Rectum	II	2836.5	2.7	870.0	4.6
SYSUCC-562	SYSUCC	Male	78	Rectum	I	1993.8	15.5	--	--
SYSUCC-563	SYSUCC	Female	74	Rectum	I	1568.2	1.6	--	--
SYSUCC-564	SYSUCC	Female	64	Colon	I	1531.4	13.8	--	--
SYSUCC-565	SYSUCC	Female	61	Colon	I	1641.7	3.4	--	--
SYSUCC-566	SYSUCC	Female	53	Rectum	I	2282.6	6.0	--	--
SYSUCC-567	SYSUCC	Female	72	Colon	I	1221.2	13.7	--	--
SYSUCC-568	SYSUCC	Male	61	Colon	I	2313.6	10.5	--	--
SYSUCC-569	SYSUCC	Male	54	Colon	I	2631.4	10.9	--	--
SYSUCC-570	SYSUCC	Female	64	Rectum	I	1900.3	6.4	--	--
SYSUCC-571	SYSUCC	Male	65	Rectum	I	2066.6	19.9	--	--
SYSUCC-572	SYSUCC	Male	60	Colon	I	2915.4	11.5	--	--
SYSUCC-573	SYSUCC	Female	51	Rectum	I	2495.8	7.4	--	--
SYSUCC-574	SYSUCC	Female	52	Colon	I	1735.9	17.2	--	--
SYSUCC-575	SYSUCC	Female	61	Rectum	I	1522.4	3.9	--	--
SYSUCC-576	SYSUCC	Male	61	Colon	I	2652.4	4.6	--	--
SYSUCC-577	SYSUCC	Male	58	Colon	I	2825.0	3.9	--	--
SYSUCC-578	SYSUCC	Male	47	Rectum	I	2691.0	7.7	--	--
CHCAMS-001	CHCAMS	Male	33	Rectum	II	915.3	21.6	--	--
CHCAMS-002	CHCAMS	Male	70	Rectum	III	1212.5	5.0	--	--
CHCAMS-003	CHCAMS	Male	33	Rectum	III	1271.9	3.1	--	--
CHCAMS-004	CHCAMS	Male	39	Rectum	III	1199.8	21.6	--	--
CHCAMS-005	CHCAMS	Male	59	Rectum	III	2030.1	9.6	--	--
CHCAMS-006	CHCAMS	Female	51	Rectum	III	1155.9	17.4	--	--
CHCAMS-007	CHCAMS	Male	43	Rectum	I	1431.5	4.9	--	--
CHCAMS-008	CHCAMS	Male	70	Rectum	II	1141.1	10.6	--	--
CHCAMS-009	CHCAMS	Male	56	Rectum	II	2113.6	12.2	--	--
CHCAMS-010	CHCAMS	Male	73	Rectum	II	2180.6	13.3	--	--
CHCAMS-011	CHCAMS	Male	59	Rectum	II	2361.9	6.9	--	--
CHCAMS-012	CHCAMS	Male	58	Rectum	II	2572.5	1.8	--	--
CHCAMS-013	CHCAMS	Male	37	Rectum	III	1493.2	8.6	--	--
CHCAMS-014	CHCAMS	Male	62	Rectum	III	1636.3	13.0	--	--
CHCAMS-015	CHCAMS	Male	45	Rectum	III	1318.0	22.5	--	--
CHCAMS-016	CHCAMS	Male	51	Rectum	III	2041.6	10.1	--	--
CHCAMS-017	CHCAMS	Male	42	Rectum	III	2158.3	9.4	--	--
CHCAMS-018	CHCAMS	Male	60	Rectum	III	1806.8	13.4	--	--
CHCAMS-019	CHCAMS	Male	44	Rectum	III	1640.5	7.5	--	--

CHCAMS-020	CHCAMS	Male	38	Rectum	III	2652.5	20.5	--	--
CHCAMS-021	CHCAMS	Female	37	Rectum	III	1410.9	12.2	--	--
CHCAMS-022	CHCAMS	Female	66	Rectum	III	3027.3	17.8	--	--
CHCAMS-023	CHCAMS	Female	54	Rectum	III	2307.9	6.0	--	--
CHCAMS-024	CHCAMS	Female	26	Rectum	III	3032.8	10.4	--	--
CHCAMS-025	CHCAMS	Female	51	Rectum	III	2601.4	6.8	--	--
CHCAMS-026	CHCAMS	Male	41	Rectum	II	1387.8	9.4	--	--
CHCAMS-027	CHCAMS	Male	60	Rectum	II	1195.0	24.0	--	--
CHCAMS-028	CHCAMS	Male	74	Rectum	II	2039.6	4.8	--	--
CHCAMS-029	CHCAMS	Male	38	Rectum	II	2572.9	13.4	--	--
CHCAMS-030	CHCAMS	Female	50	Rectum	II	2123.5	33.0	--	--
CHCAMS-031	CHCAMS	Male	57	Rectum	III	1163.5	5.3	--	--
CHCAMS-032	CHCAMS	Male	82	Rectum	III	1119.6	12.0	--	--
CHCAMS-033	CHCAMS	Male	54	Rectum	III	1597.7	18.9	--	--
CHCAMS-034	CHCAMS	Male	50	Rectum	III	1487.0	18.9	--	--
CHCAMS-035	CHCAMS	Male	54	Rectum	III	1115.5	10.4	--	--
CHCAMS-036	CHCAMS	Male	38	Rectum	III	1536.4	9.8	--	--
CHCAMS-037	CHCAMS	Male	67	Rectum	III	2079.8	13.9	--	--
CHCAMS-038	CHCAMS	Male	69	Rectum	III	1109.7	24.6	--	--
CHCAMS-039	CHCAMS	Male	58	Rectum	III	2099.1	12.3	--	--
CHCAMS-040	CHCAMS	Male	65	Rectum	III	1733.7	6.2	--	--
CHCAMS-041	CHCAMS	Male	70	Rectum	III	2361.3	12.4	--	--
CHCAMS-042	CHCAMS	Male	36	Rectum	III	2653.9	11.8	--	--
CHCAMS-043	CHCAMS	Male	57	Rectum	III	2692.2	21.3	--	--
CHCAMS-044	CHCAMS	Female	49	Rectum	III	1207.4	10.2	--	--
CHCAMS-045	CHCAMS	Female	41	Rectum	III	1181.0	12.6	--	--
CHCAMS-046	CHCAMS	Female	43	Rectum	III	1351.5	20.0	--	--
CHCAMS-047	CHCAMS	Female	57	Rectum	III	2194.2	1.0	--	--
CHCAMS-048	CHCAMS	Female	34	Rectum	III	2203.5	5.7	--	--
CHCAMS-049	CHCAMS	Male	48	Rectum	III	1777.3	4.5	--	--
CHCAMS-050	CHCAMS	Male	29	Rectum	III	1820.2	15.2	--	--
CHCAMS-051	CHCAMS	Female	41	Rectum	III	2361.1	7.5	--	--
CHCAMS-052	CHCAMS	Male	67	Rectum	II	2607.9	1.8	--	--
CHCAMS-053	CHCAMS	Male	71	Rectum	II	2253.9	14.5	--	--
CHCAMS-054	CHCAMS	Male	66	Rectum	III	2659.4	33.5	--	--
CHCAMS-055	CHCAMS	Male	47	Rectum	III	2815.2	6.7	--	--
CHCAMS-056	CHCAMS	Male	51	Rectum	II	3401.0	6.8	--	--
CHCAMS-057	CHCAMS	Female	54	Rectum	II	3240.9	11.4	--	--
CHCAMS-058	CHCAMS	Male	64	Rectum	III	2790.1	18.1	--	--
CHCAMS-059	CHCAMS	Male	75	Rectum	III	1910.8	3.1	--	--
CHCAMS-060	CHCAMS	Male	47	Rectum	III	2126.3	13.4	--	--
CHCAMS-061	CHCAMS	Male	47	Rectum	III	2624.4	9.1	--	--
CHCAMS-062	CHCAMS	Female	67	Rectum	III	2196.9	5.1	--	--
CHCAMS-063	CHCAMS	Female	39	Rectum	III	2094.5	6.7	--	--

CHCAMS-064	CHCAMS	Female	48	Rectum	III	2375.2	8.2	--	--
CHCAMS-065	CHCAMS	Female	72	Rectum	III	2239.7	1.8	--	--
CHCAMS-066	CHCAMS	Female	67	Rectum	III	2500.1	8.1	--	--
CHCAMS-067	CHCAMS	Male	44	Rectum	II	2479.4	2.2	--	--
CHCAMS-068	CHCAMS	Male	72	Rectum	II	3092.2	11.0	--	--
CHCAMS-069	CHCAMS	Male	29	Rectum	II	3092.8	8.8	--	--
CHCAMS-070	CHCAMS	Female	44	Rectum	II	2360.8	10.0	--	--
CHCAMS-071	CHCAMS	Female	51	Rectum	II	2566.4	25.9	--	--
CHCAMS-072	CHCAMS	Female	54	Rectum	II	2767.2	14.1	--	--
CHCAMS-073	CHCAMS	Female	62	Rectum	II	2875.5	14.5	--	--
CHCAMS-074	CHCAMS	Male	50	Rectum	III	2768.9	19.2	--	--
CHCAMS-075	CHCAMS	Male	55	Rectum	III	2333.9	28.3	--	--
CHCAMS-076	CHCAMS	Male	62	Rectum	III	3024.2	12.3	--	--
CHCAMS-077	CHCAMS	Male	34	Rectum	III	3178.9	10.0	--	--
CHCAMS-078	CHCAMS	Male	71	Rectum	III	2532.0	20.4	--	--
CHCAMS-079	CHCAMS	Male	34	Rectum	III	2748.1	20.4	--	--
CHCAMS-080	CHCAMS	Male	55	Rectum	III	3288.4	9.2	--	--
CHCAMS-081	CHCAMS	Male	58	Rectum	III	3107.3	18.9	--	--
CHCAMS-082	CHCAMS	Male	68	Rectum	III	3548.8	16.4	--	--
CHCAMS-083	CHCAMS	Male	59	Rectum	III	3039.4	16.4	--	--
CHCAMS-084	CHCAMS	Male	42	Rectum	III	3342.3	9.1	--	--
CHCAMS-085	CHCAMS	Male	53	Rectum	III	2905.4	12.8	--	--
CHCAMS-086	CHCAMS	Male	36	Rectum	III	3095.6	6.9	--	--
CHCAMS-087	CHCAMS	Female	72	Rectum	III	2426.3	7.9	--	--
CHCAMS-088	CHCAMS	Female	47	Rectum	III	2415.1	12.2	--	--
CHCAMS-089	CHCAMS	Female	56	Rectum	III	3332.6	15.2	--	--
CHCAMS-090	CHCAMS	Female	56	Rectum	III	2808.7	5.6	--	--
CHCAMS-091	CHCAMS	Male	73	Rectum	II	2888.3	12.5	--	--
CHCAMS-092	CHCAMS	Male	74	Rectum	II	3059.0	3.5	--	--
CHCAMS-093	CHCAMS	Male	58	Rectum	III	2400.3	6.2	--	--
CHCAMS-094	CHCAMS	Male	30	Rectum	III	3303.7	11.2	--	--
CHCAMS-095	CHCAMS	Male	60	Rectum	III	3290.0	1.2	--	--
CHCAMS-096	CHCAMS	Male	45	Rectum	III	3659.4	12.4	--	--
CHCAMS-097	CHCAMS	Male	70	Rectum	III	4865.8	19.2	--	--
CHCAMS-098	CHCAMS	Female	58	Rectum	III	2317.2	17.8	--	--
CHCAMS-099	CHCAMS	Female	56	Rectum	III	2638.4	16.2	--	--
CHCAMS-100	CHCAMS	Female	50	Rectum	III	3404.0	9.6	--	--
CHCAMS-101	CHCAMS	Male	56	Rectum	II	3548.5	17.9	--	--
CHCAMS-102	CHCAMS	Female	62	Rectum	III	4870.8	2.4	--	--
SYSUCC-001	SYSUCC	Female	70	Colon	IV	3105.8	7.5	--	--
SYSUCC-002	SYSUCC	Female	43	Colon	I	2166.7	2.9	--	--
SYSUCC-003	SYSUCC	Female	61	Colon	IV	1622.8	12.2	--	--
SYSUCC-004	SYSUCC	Male	37	Rectum	II	1292.7	21.5	--	--
SYSUCC-005	SYSUCC	Female	41	Rectum	II	2148.1	8.0	--	--

SYSUCC-006	SYSUCC	Male	78	Rectum	II	1931.8	14.5	--	--
SYSUCC-007	SYSUCC	Female	69	Rectum	II	4983.3	18.7	--	--
SYSUCC-008	SYSUCC	Female	39	Rectum	II	2146.8	8.4	--	--
SYSUCC-009	SYSUCC	Male	57	Colon	II	4646.3	3.0	--	--
SYSUCC-010	SYSUCC	Male	73	Colon	II	2194.2	9.2	--	--
SYSUCC-011	SYSUCC	Female	54	Rectum	I	2821.1	14.0	--	--
SYSUCC-012	SYSUCC	Male	60	Colon	IV	2019.1	9.5	--	--
SYSUCC-013	SYSUCC	Male	68	Colon	IV	2384.3	7.7	--	--
SYSUCC-014	SYSUCC	Male	65	Rectum	I	1715.1	14.9	--	--
SYSUCC-015	SYSUCC	Female	80	Rectum	I	1246.4	5.9	--	--
SYSUCC-016	SYSUCC	Male	40	Colon	IV	4120.5	8.4	--	--
SYSUCC-017	SYSUCC	Male	38	Colon	IV	1206.7	14.3	--	--
SYSUCC-018	SYSUCC	Female	72	Colon	III	3195.3	8.5	--	--
SYSUCC-019	SYSUCC	Male	72	Colon	IV	1233.4	22.6	--	--
SYSUCC-020	SYSUCC	Female	60	Rectum	IV	3142.9	10.2	--	--
SYSUCC-021	SYSUCC	Female	73	Colon	I	3496.1	9.8	--	--
SYSUCC-022	SYSUCC	Female	45	Colon	I	2358.8	10.4	--	--
SYSUCC-023	SYSUCC	Male	69	Rectum	IV	1842.2	4.2	--	--
SYSUCC-024	SYSUCC	Male	47	Colon	IV	4082.7	9.8	--	--
SYSUCC-025	SYSUCC	Female	78	Colon	I	1314.1	1.9	--	--
SYSUCC-026	SYSUCC	Male	60	Rectum	I	2452.9	6.4	--	--
SYSUCC-027	SYSUCC	Male	59	Rectum	I	1878.9	13.9	--	--
SYSUCC-028	SYSUCC	Male	72	Rectum	II	3277.8	14.8	--	--
SYSUCC-029	SYSUCC	Male	75	Rectum	IV	3725.6	19.1	--	--
SYSUCC-030	SYSUCC	Male	72	Rectum	II	2178.9	11.2	--	--
SYSUCC-031	SYSUCC	Male	58	Colon	IV	2540.5	4.2	--	--
SYSUCC-032	SYSUCC	Male	72	Colon	IV	2342.2	8.1	--	--
SYSUCC-033	SYSUCC	Female	57	Rectum	II	1370.9	10.0	--	--
SYSUCC-034	SYSUCC	Female	52	Rectum	IV	2797.0	15.9	--	--
SYSUCC-035	SYSUCC	Female	36	Rectum	III	3041.3	9.9	--	--
SYSUCC-036	SYSUCC	Male	54	Rectum	IV	1253.2	6.0	--	--
SYSUCC-037	SYSUCC	Male	46	Rectum	III	1999.5	14.1	--	--
SYSUCC-038	SYSUCC	Female	61	Rectum	III	1468.8	14.3	--	--
SYSUCC-039	SYSUCC	Male	62	Rectum	III	1534.1	15.0	--	--
SYSUCC-040	SYSUCC	Male	54	Rectum	III	3390.4	11.8	--	--
SYSUCC-041	SYSUCC	Male	60	Rectum	IV	2000.0	17.1	--	--
SYSUCC-042	SYSUCC	Female	71	Rectum	III	1280.1	14.8	--	--
SYSUCC-043	SYSUCC	Male	49	Colon	III	2642.7	11.2	--	--
SYSUCC-044	SYSUCC	Male	62	Colon	III	3340.2	8.0	--	--
SYSUCC-045	SYSUCC	Male	38	Rectum	IV	2868.4	11.0	--	--
SYSUCC-046	SYSUCC	Male	76	Colon	III	2479.4	8.2	--	--
SYSUCC-047	SYSUCC	Female	60	Rectum	IV	3127.9	11.3	--	--
SYSUCC-048	SYSUCC	Male	66	Colon	III	2940.5	10.4	--	--
SYSUCC-049	SYSUCC	Male	71	Colon	IV	2619.3	8.1	--	--

SYSUCC-050	SYSUCC	Female	45	Colon	II	3032.7	20.6	--	--
SYSUCC-051	SYSUCC	Female	59	Colon	IV	1382.0	6.7	--	--
SYSUCC-052	SYSUCC	Male	62	Rectum	IV	2762.3	6.0	--	--
SYSUCC-053	SYSUCC	Female	54	Rectum	IV	2375.0	13.5	--	--
SYSUCC-054	SYSUCC	Male	72	Colon	III	2280.4	11.0	--	--
SYSUCC-055	SYSUCC	Male	40	Rectum	IV	3110.2	16.2	--	--
SYSUCC-056	SYSUCC	Female	71	Colon	II	1307.2	19.9	--	--
SYSUCC-057	SYSUCC	Female	56	Colon	III	2594.2	8.1	--	--
SYSUCC-058	SYSUCC	Male	65	Rectum	III	2167.1	26.6	--	--
SYSUCC-059	SYSUCC	Male	46	Colon	II	1849.9	25.5	--	--
SYSUCC-060	SYSUCC	Male	36	Colon	IV	2166.4	2.2	--	--
SYSUCC-061	SYSUCC	Female	67	Rectum	III	2489.5	10.4	--	--
SYSUCC-062	SYSUCC	Male	39	Colon	III	3055.8	0.3	--	--
SYSUCC-063	SYSUCC	Female	30	Colon	II	2015.9	15.0	--	--
SYSUCC-064	SYSUCC	Female	77	Colon	IV	3205.4	8.6	--	--
SYSUCC-065	SYSUCC	Male	63	Rectum	III	2627.8	19.4	--	--
SYSUCC-066	SYSUCC	Male	46	Rectum	I	1879.3	17.5	--	--
SYSUCC-067	SYSUCC	Male	66	Colon	IV	3350.1	17.5	--	--
SYSUCC-068	SYSUCC	Male	44	Colon	II	1137.8	12.3	--	--
SYSUCC-069	SYSUCC	Male	69	Rectum	IV	2560.9	16.3	--	--
SYSUCC-070	SYSUCC	Male	43	Colon	IV	2081.8	7.1	--	--
SYSUCC-071	SYSUCC	Male	57	Colon	IV	2030.8	23.5	--	--
SYSUCC-072	SYSUCC	Female	62	Colon	III	2843.8	18.1	--	--
SYSUCC-073	SYSUCC	Male	65	Colon	III	6327.6	18.4	--	--
SYSUCC-074	SYSUCC	Female	63	Rectum	II	2318.3	22.8	--	--
SYSUCC-075	SYSUCC	Female	44	Colon	III	2764.3	21.4	--	--
SYSUCC-076	SYSUCC	Female	68	Rectum	II	2969.4	7.5	--	--
SYSUCC-077	SYSUCC	Female	46	Rectum	III	5735.3	19.6	--	--
SYSUCC-078	SYSUCC	Female	78	Rectum	III	5779.9	7.1	--	--
SYSUCC-079	SYSUCC	Female	61	Colon	IV	1818.6	7.6	--	--
SYSUCC-080	SYSUCC	Male	51	Rectum	III	2202.2	15.9	--	--
SYSUCC-081	SYSUCC	Female	59	Rectum	I	2663.3	3.5	--	--
SYSUCC-082	SYSUCC	Male	70	Colon	III	1406.9	7.2	--	--
SYSUCC-083	SYSUCC	Male	39	Colon	II	3900.8	9.2	--	--
SYSUCC-084	SYSUCC	Male	66	Rectum	III	1777.9	16.8	--	--
SYSUCC-085	SYSUCC	Female	65	Rectum	II	2414.9	11.0	--	--
SYSUCC-086	SYSUCC	Female	52	Rectum	IV	2539.9	15.7	--	--
SYSUCC-087	SYSUCC	Male	33	Colon	II	1337.4	4.3	--	--
SYSUCC-088	SYSUCC	Female	34	Colon	II	4030.4	10.7	--	--
SYSUCC-089	SYSUCC	Female	74	Colon	IV	3449.7	7.1	--	--
SYSUCC-090	SYSUCC	Female	58	Colon	IV	1881.2	3.3	--	--
SYSUCC-091	SYSUCC	Male	49	Colon	II	1880.3	20.2	--	--
SYSUCC-092	SYSUCC	Male	44	Rectum	III	2201.3	8.6	--	--
SYSUCC-093	SYSUCC	Male	77	Rectum	IV	1487.3	12.1	--	--

SYSUCC-094	SYSUCC	Female	42	Rectum	II	3132.5	12.4	--	--
SYSUCC-095	SYSUCC	Male	61	Rectum	IV	3147.1	17.9	--	--
SYSUCC-096	SYSUCC	Male	59	Colon	II	1954.1	10.8	--	--
SYSUCC-097	SYSUCC	Male	54	Colon	IV	2122.7	4.1	--	--
SYSUCC-098	SYSUCC	Male	60	Colon	IV	2469.6	21.8	--	--
SYSUCC-099	SYSUCC	Female	62	Colon	III	2363.4	7.1	--	--
SYSUCC-100	SYSUCC	Male	76	Rectum	III	2043.0	8.9	--	--
SYSUCC-101	SYSUCC	Male	45	Rectum	IV	2783.4	8.8	--	--
SYSUCC-102	SYSUCC	Male	62	Colon	III	2416.1	5.9	--	--
SYSUCC-103	SYSUCC	Female	56	Rectum	III	2388.6	29.5	--	--
SYSUCC-104	SYSUCC	Female	40	Colon	IV	1986.1	4.9	--	--
SYSUCC-105	SYSUCC	Male	59	Colon	II	2233.0	16.9	--	--
SYSUCC-106	SYSUCC	Male	52	Rectum	II	2724.2	20.5	--	--
SYSUCC-107	SYSUCC	Male	74	Colon	IV	5061.4	9.7	--	--
SYSUCC-108	SYSUCC	Male	48	Colon	IV	3372.1	7.1	--	--
SYSUCC-109	SYSUCC	Female	36	Colon	III	3279.5	2.5	--	--
SYSUCC-110	SYSUCC	Female	53	Colon	II	1948.8	11.0	--	--
SYSUCC-111	SYSUCC	Male	38	Colon	IV	3346.0	5.7	--	--
SYSUCC-112	SYSUCC	Female	53	Colon	III	2490.4	6.2	--	--
SYSUCC-113	SYSUCC	Female	55	Colon	IV	2722.0	7.3	--	--
SYSUCC-114	SYSUCC	Female	45	Colon	IV	3699.5	12.9	--	--
SYSUCC-115	SYSUCC	Male	50	Colon	IV	3277.6	11.0	--	--
SYSUCC-116	SYSUCC	Male	51	Colon	IV	3234.5	16.8	--	--
SYSUCC-117	SYSUCC	Female	55	Colon	II	2860.2	7.1	--	--
SYSUCC-118	SYSUCC	Female	58	Colon	IV	2532.4	8.9	--	--
SYSUCC-119	SYSUCC	Female	43	Colon	IV	3412.0	11.4	--	--
SYSUCC-120	SYSUCC	Female	62	Colon	IV	4587.6	7.5	--	--
SYSUCC-121	SYSUCC	Male	52	Rectum	II	1699.7	23.4	--	--
SYSUCC-122	SYSUCC	Male	48	Rectum	II	1416.7	21.1	--	--
SYSUCC-123	SYSUCC	Male	72	Rectum	II	1142.1	7.0	--	--
SYSUCC-124	SYSUCC	Male	58	Rectum	II	1559.9	2.5	--	--
SYSUCC-125	SYSUCC	Male	40	Rectum	II	1179.2	17.9	--	--
SYSUCC-126	SYSUCC	Male	75	Rectum	II	1281.2	4.9	--	--
SYSUCC-127	SYSUCC	Male	61	Rectum	II	1410.0	7.6	--	--
SYSUCC-128	SYSUCC	Male	72	Rectum	II	1327.5	14.7	--	--
SYSUCC-129	SYSUCC	Male	54	Rectum	II	1818.3	4.4	--	--
SYSUCC-130	SYSUCC	Male	69	Rectum	II	2210.9	9.1	--	--
SYSUCC-131	SYSUCC	Male	48	Rectum	II	2187.9	8.7	--	--
SYSUCC-132	SYSUCC	Male	65	Rectum	II	2847.9	8.7	--	--
SYSUCC-133	SYSUCC	Male	59	Rectum	II	2631.4	9.0	--	--
SYSUCC-134	SYSUCC	Male	61	Rectum	II	2350.7	27.8	--	--
SYSUCC-135	SYSUCC	Male	52	Rectum	II	2120.5	7.6	--	--
SYSUCC-136	SYSUCC	Male	65	Rectum	II	1373.8	14.5	--	--
SYSUCC-137	SYSUCC	Male	63	Rectum	II	1259.7	3.1	--	--

SYSUCC-138	SYSUCC	Male	58	Rectum	II	1235.9	17.7	--	--
SYSUCC-139	SYSUCC	Male	50	Rectum	II	1538.1	6.9	--	--
SYSUCC-140	SYSUCC	Male	60	Rectum	II	2101.4	22.6	--	--
SYSUCC-141	SYSUCC	Male	54	Rectum	II	1949.0	28.5	--	--
SYSUCC-142	SYSUCC	Male	64	Rectum	II	2638.3	12.7	--	--
SYSUCC-143	SYSUCC	Male	34	Rectum	II	2310.7	1.2	--	--
SYSUCC-144	SYSUCC	Male	36	Rectum	III	773.8	13.5	--	--
SYSUCC-145	SYSUCC	Male	43	Rectum	III	1149.2	3.4	--	--
SYSUCC-146	SYSUCC	Male	55	Rectum	III	1380.8	3.0	--	--
SYSUCC-147	SYSUCC	Male	56	Rectum	III	1466.2	22.1	--	--
SYSUCC-148	SYSUCC	Male	47	Rectum	III	1541.8	6.0	--	--
SYSUCC-149	SYSUCC	Male	62	Rectum	III	1495.4	11.1	--	--
SYSUCC-150	SYSUCC	Male	61	Rectum	III	1283.6	16.2	--	--
SYSUCC-151	SYSUCC	Male	60	Rectum	III	1155.3	10.0	--	--
SYSUCC-152	SYSUCC	Male	41	Rectum	III	1238.8	7.8	--	--
SYSUCC-153	SYSUCC	Male	68	Rectum	III	1251.7	20.7	--	--
SYSUCC-154	SYSUCC	Male	43	Rectum	III	1323.7	4.2	--	--
SYSUCC-155	SYSUCC	Male	58	Rectum	III	1811.7	15.7	--	--
SYSUCC-156	SYSUCC	Male	74	Rectum	III	1479.8	8.4	--	--
SYSUCC-157	SYSUCC	Male	70	Rectum	III	1149.9	7.8	--	--
SYSUCC-158	SYSUCC	Male	60	Rectum	III	1708.4	12.0	--	--
SYSUCC-159	SYSUCC	Male	70	Rectum	III	1899.5	4.1	--	--
SYSUCC-160	SYSUCC	Male	53	Rectum	III	2052.6	14.1	--	--
SYSUCC-161	SYSUCC	Male	65	Rectum	III	2090.3	12.1	--	--
SYSUCC-162	SYSUCC	Male	57	Rectum	III	2563.5	8.2	--	--
SYSUCC-163	SYSUCC	Male	30	Rectum	III	2965.2	8.2	--	--
SYSUCC-164	SYSUCC	Male	73	Rectum	III	2577.3	20.4	--	--
SYSUCC-165	SYSUCC	Male	41	Rectum	III	2016.6	19.2	--	--
SYSUCC-166	SYSUCC	Male	62	Rectum	III	2417.4	21.4	--	--
SYSUCC-167	SYSUCC	Male	49	Rectum	III	2636.3	4.3	--	--
SYSUCC-168	SYSUCC	Male	62	Rectum	III	2001.9	11.4	--	--
SYSUCC-169	SYSUCC	Male	41	Rectum	III	2316.4	8.2	--	--
SYSUCC-170	SYSUCC	Male	56	Rectum	III	2405.0	12.8	--	--
SYSUCC-171	SYSUCC	Male	66	Rectum	III	2254.5	4.5	--	--
SYSUCC-172	SYSUCC	Male	30	Rectum	III	1196.4	5.6	--	--
SYSUCC-173	SYSUCC	Male	66	Rectum	III	1180.6	11.9	--	--
SYSUCC-174	SYSUCC	Male	41	Rectum	III	1542.3	17.5	--	--
SYSUCC-175	SYSUCC	Male	67	Rectum	III	1721.5	13.0	--	--
SYSUCC-176	SYSUCC	Male	34	Rectum	III	1324.7	11.8	--	--
SYSUCC-177	SYSUCC	Male	52	Rectum	III	1153.2	17.2	--	--
SYSUCC-178	SYSUCC	Male	54	Rectum	III	1329.2	13.9	--	--
SYSUCC-179	SYSUCC	Male	56	Rectum	III	2347.0	16.1	--	--
SYSUCC-180	SYSUCC	Male	65	Rectum	III	1584.4	11.3	--	--
SYSUCC-181	SYSUCC	Male	70	Rectum	III	1480.0	12.9	--	--

SYSUCC-182	SYSUCC	Male	72	Rectum	III	1101.3	5.7	--	--
SYSUCC-183	SYSUCC	Male	55	Rectum	III	1122.2	14.2	--	--
SYSUCC-184	SYSUCC	Male	71	Rectum	III	1371.3	11.6	--	--
SYSUCC-185	SYSUCC	Male	67	Rectum	III	1841.7	9.4	--	--
SYSUCC-186	SYSUCC	Male	38	Rectum	III	2776.5	19.8	--	--
SYSUCC-187	SYSUCC	Male	77	Rectum	III	2126.1	13.7	--	--
SYSUCC-188	SYSUCC	Male	34	Rectum	III	2493.8	10.3	--	--
SYSUCC-189	SYSUCC	Male	69	Rectum	III	2002.7	31.6	--	--
SYSUCC-190	SYSUCC	Male	23	Rectum	III	2752.5	9.5	--	--
SYSUCC-191	SYSUCC	Male	67	Rectum	III	2329.6	17.7	--	--
SYSUCC-192	SYSUCC	Male	43	Rectum	III	2885.7	7.0	--	--
SYSUCC-193	SYSUCC	Male	67	Rectum	IV	1167.7	31.5	--	--
SYSUCC-194	SYSUCC	Male	60	Rectum	IV	1720.2	14.0	--	--
SYSUCC-195	SYSUCC	Male	53	Rectum	IV	2428.4	28.2	--	--
SYSUCC-196	SYSUCC	Male	60	Rectum	IV	2594.2	10.5	--	--
SYSUCC-197	SYSUCC	Male	60	Rectum	IV	2070.2	15.8	--	--
SYSUCC-198	SYSUCC	Male	48	Rectum	IV	2024.0	22.4	--	--
SYSUCC-199	SYSUCC	Male	75	Rectum	IV	2810.7	3.6	--	--
SYSUCC-200	SYSUCC	Male	56	Rectum	II	1956.4	12.9	--	--
SYSUCC-201	SYSUCC	Male	61	Rectum	II	2246.5	5.9	--	--
SYSUCC-202	SYSUCC	Male	66	Rectum	II	2198.8	9.2	--	--
SYSUCC-203	SYSUCC	Male	67	Rectum	II	2660.1	12.1	--	--
SYSUCC-204	SYSUCC	Male	65	Rectum	II	2728.1	24.6	--	--
SYSUCC-205	SYSUCC	Male	48	Rectum	II	3055.0	7.1	--	--
SYSUCC-206	SYSUCC	Male	65	Rectum	II	2678.8	14.8	--	--
SYSUCC-207	SYSUCC	Male	47	Rectum	II	3237.0	8.0	--	--
SYSUCC-208	SYSUCC	Male	48	Rectum	II	2590.5	7.4	--	--
SYSUCC-209	SYSUCC	Male	66	Rectum	II	2554.5	14.6	--	--
SYSUCC-210	SYSUCC	Male	52	Rectum	II	3200.1	5.0	--	--
SYSUCC-211	SYSUCC	Male	60	Rectum	II	2666.7	16.5	--	--
SYSUCC-212	SYSUCC	Male	59	Rectum	II	2877.9	13.7	--	--
SYSUCC-213	SYSUCC	Male	77	Rectum	II	2918.0	12.4	--	--
SYSUCC-214	SYSUCC	Male	62	Rectum	II	2771.9	16.6	--	--
SYSUCC-215	SYSUCC	Male	50	Rectum	II	3192.8	2.5	--	--
SYSUCC-216	SYSUCC	Male	60	Rectum	II	4523.1	6.6	--	--
SYSUCC-217	SYSUCC	Male	70	Rectum	II	3103.9	3.9	--	--
SYSUCC-218	SYSUCC	Male	32	Rectum	II	2267.0	9.7	--	--
SYSUCC-219	SYSUCC	Male	62	Rectum	II	2767.2	12.4	--	--
SYSUCC-220	SYSUCC	Male	68	Rectum	II	3394.0	17.7	--	--
SYSUCC-221	SYSUCC	Male	61	Rectum	III	2069.1	11.9	--	--
SYSUCC-222	SYSUCC	Male	62	Rectum	III	2333.8	2.4	--	--
SYSUCC-223	SYSUCC	Male	67	Rectum	III	2124.8	19.4	--	--
SYSUCC-224	SYSUCC	Male	71	Rectum	III	2073.5	7.8	--	--
SYSUCC-225	SYSUCC	Male	74	Rectum	III	2169.0	5.1	--	--

SYSUCC-226	SYSUCC	Male	45	Rectum	III	2687.2	12.7	--	--
SYSUCC-227	SYSUCC	Male	56	Rectum	III	2341.6	14.5	--	--
SYSUCC-228	SYSUCC	Male	66	Rectum	III	2304.1	3.1	--	--
SYSUCC-229	SYSUCC	Male	76	Rectum	III	3196.2	10.2	--	--
SYSUCC-230	SYSUCC	Male	62	Rectum	III	2784.9	5.4	--	--
SYSUCC-231	SYSUCC	Male	82	Rectum	III	3096.5	15.9	--	--
SYSUCC-232	SYSUCC	Male	35	Rectum	III	3025.2	8.7	--	--
SYSUCC-233	SYSUCC	Male	69	Rectum	III	2798.1	9.9	--	--
SYSUCC-234	SYSUCC	Male	32	Rectum	III	3248.8	3.3	--	--
SYSUCC-235	SYSUCC	Male	56	Rectum	III	3381.4	3.3	--	--
SYSUCC-236	SYSUCC	Male	60	Rectum	III	3442.6	29.3	--	--
SYSUCC-237	SYSUCC	Male	56	Rectum	III	3315.1	5.9	--	--
SYSUCC-238	SYSUCC	Male	73	Rectum	III	3278.8	14.0	--	--
SYSUCC-239	SYSUCC	Male	66	Rectum	III	3433.6	9.5	--	--
SYSUCC-240	SYSUCC	Male	52	Rectum	III	2787.4	9.4	--	--
SYSUCC-241	SYSUCC	Male	40	Rectum	III	2322.9	15.7	--	--
SYSUCC-242	SYSUCC	Male	60	Rectum	III	2785.8	11.3	--	--
SYSUCC-243	SYSUCC	Male	76	Rectum	III	3167.0	2.1	--	--
SYSUCC-244	SYSUCC	Male	50	Rectum	III	2544.4	5.8	--	--
SYSUCC-245	SYSUCC	Male	67	Rectum	III	2714.4	6.4	--	--
SYSUCC-246	SYSUCC	Male	60	Rectum	III	2221.6	10.8	--	--
SYSUCC-247	SYSUCC	Male	52	Rectum	III	3150.8	1.0	--	--
SYSUCC-248	SYSUCC	Male	27	Rectum	III	3217.1	8.4	--	--
SYSUCC-249	SYSUCC	Male	60	Rectum	IV	2567.8	4.7	--	--
SYSUCC-250	SYSUCC	Male	58	Rectum	IV	2282.3	9.2	--	--
SYSUCC-251	SYSUCC	Male	54	Rectum	IV	2332.2	8.0	--	--
SYSUCC-252	SYSUCC	Male	49	Rectum	IV	2557.5	13.8	--	--
SYSUCC-253	SYSUCC	Male	68	Rectum	IV	3280.9	10.7	--	--
SYSUCC-254	SYSUCC	Male	60	Rectum	IV	2364.8	4.2	--	--
SYSUCC-255	SYSUCC	Male	58	Rectum	IV	2509.9	9.8	--	--
SYSUCC-256	SYSUCC	Male	54	Rectum	IV	2276.2	14.3	--	--
SYSUCC-257	SYSUCC	Male	67	Rectum	IV	2410.5	3.6	--	--
SYSUCC-258	SYSUCC	Male	68	Rectum	IV	2660.5	9.5	--	--
SYSUCC-259	SYSUCC	Male	41	Rectum	IV	3559.6	9.5	--	--
SYSUCC-260	SYSUCC	Male	49	Rectum	IV	3645.3	5.0	--	--
SYSUCC-261	SYSUCC	Male	54	Rectum	IV	3627.9	19.2	--	--
SYSUCC-262	SYSUCC	Male	67	Rectum	IV	3085.9	20.2	--	--
SYSUCC-263	SYSUCC	Female	58	Rectum	II	1658.8	13.4	--	--
SYSUCC-264	SYSUCC	Female	58	Rectum	II	2068.9	11.1	--	--
SYSUCC-265	SYSUCC	Female	68	Rectum	II	2216.6	22.4	--	--
SYSUCC-266	SYSUCC	Female	62	Rectum	II	1334.9	11.6	--	--
SYSUCC-267	SYSUCC	Female	59	Rectum	II	2346.6	7.7	--	--
SYSUCC-268	SYSUCC	Female	77	Rectum	II	1901.0	11.7	--	--
SYSUCC-269	SYSUCC	Female	70	Rectum	II	2350.7	14.6	--	--

SYSUCC-270	SYSUCC	Female	43	Rectum	II	2726.1	10.5	--	--
SYSUCC-271	SYSUCC	Female	41	Rectum	III	2569.7	12.4	--	--
SYSUCC-272	SYSUCC	Female	50	Rectum	III	1829.5	19.0	--	--
SYSUCC-273	SYSUCC	Female	32	Rectum	III	1706.2	25.0	--	--
SYSUCC-274	SYSUCC	Female	34	Rectum	III	1444.4	7.6	--	--
SYSUCC-275	SYSUCC	Female	54	Rectum	III	1346.1	5.8	--	--
SYSUCC-276	SYSUCC	Female	63	Rectum	III	2723.4	4.5	--	--
SYSUCC-277	SYSUCC	Female	60	Rectum	III	3195.2	10.6	--	--
SYSUCC-278	SYSUCC	Female	42	Rectum	III	979.2	11.8	--	--
SYSUCC-279	SYSUCC	Female	62	Rectum	III	2589.2	6.7	--	--
SYSUCC-280	SYSUCC	Female	25	Rectum	III	999.5	11.1	--	--
SYSUCC-281	SYSUCC	Female	65	Rectum	III	1102.6	6.2	--	--
SYSUCC-282	SYSUCC	Female	51	Rectum	III	1313.6	3.7	--	--
SYSUCC-283	SYSUCC	Female	45	Rectum	III	1510.7	11.0	--	--
SYSUCC-284	SYSUCC	Female	45	Rectum	III	1155.7	14.8	--	--
SYSUCC-285	SYSUCC	Female	51	Rectum	III	1254.5	4.6	--	--
SYSUCC-286	SYSUCC	Female	55	Rectum	III	2441.2	12.7	--	--
SYSUCC-287	SYSUCC	Female	41	Rectum	III	991.4	7.2	--	--
SYSUCC-288	SYSUCC	Female	70	Rectum	III	2815.0	11.7	--	--
SYSUCC-289	SYSUCC	Female	64	Rectum	IV	2403.3	6.5	--	--
SYSUCC-290	SYSUCC	Female	36	Rectum	IV	2075.8	3.7	--	--
SYSUCC-291	SYSUCC	Female	59	Rectum	II	2346.8	18.3	--	--
SYSUCC-292	SYSUCC	Female	35	Rectum	II	2341.6	10.8	--	--
SYSUCC-293	SYSUCC	Female	68	Rectum	II	3201.9	14.4	--	--
SYSUCC-294	SYSUCC	Female	42	Rectum	II	3239.0	9.1	--	--
SYSUCC-295	SYSUCC	Female	69	Rectum	II	2859.6	8.5	--	--
SYSUCC-296	SYSUCC	Female	35	Rectum	II	3271.9	16.7	--	--
SYSUCC-297	SYSUCC	Female	74	Rectum	II	3131.9	8.0	--	--
SYSUCC-298	SYSUCC	Female	65	Rectum	II	3152.2	13.1	--	--
SYSUCC-299	SYSUCC	Female	65	Rectum	II	2580.8	9.1	--	--
SYSUCC-300	SYSUCC	Female	46	Rectum	II	3047.6	12.4	--	--
SYSUCC-301	SYSUCC	Female	41	Rectum	III	2739.3	18.9	--	--
SYSUCC-302	SYSUCC	Female	32	Rectum	III	2499.5	4.3	--	--
SYSUCC-303	SYSUCC	Female	42	Rectum	III	2419.4	14.1	--	--
SYSUCC-304	SYSUCC	Female	48	Rectum	III	2403.8	14.4	--	--
SYSUCC-305	SYSUCC	Female	64	Rectum	III	2589.6	15.6	--	--
SYSUCC-306	SYSUCC	Female	45	Rectum	III	2355.3	14.1	--	--
SYSUCC-307	SYSUCC	Female	30	Rectum	III	2871.6	2.8	--	--
SYSUCC-308	SYSUCC	Female	69	Rectum	III	2614.7	15.5	--	--
SYSUCC-309	SYSUCC	Female	49	Rectum	III	3171.7	6.8	--	--
SYSUCC-310	SYSUCC	Female	67	Rectum	III	2614.9	12.8	--	--
SYSUCC-312	SYSUCC	Female	41	Rectum	III	2568.0	8.4	--	--
SYSUCC-313	SYSUCC	Female	63	Rectum	III	3080.5	14.9	--	--
SYSUCC-314	SYSUCC	Female	71	Rectum	III	2750.2	11.8	--	--

SYSUCC-315	SYSUCC	Female	72	Rectum	III	2871.0	6.3	--	--
SYSUCC-316	SYSUCC	Female	55	Rectum	III	2876.8	10.8	--	--
SYSUCC-317	SYSUCC	Female	42	Rectum	III	2925.2	11.8	--	--
SYSUCC-318	SYSUCC	Female	67	Rectum	III	2897.8	4.4	--	--
SYSUCC-319	SYSUCC	Female	66	Rectum	III	3291.5	4.9	--	--
SYSUCC-320	SYSUCC	Female	58	Rectum	III	3285.7	11.7	--	--
SYSUCC-321	SYSUCC	Female	66	Rectum	III	2526.6	1.5	--	--
SYSUCC-322	SYSUCC	Female	37	Rectum	III	2786.3	3.5	--	--
SYSUCC-323	SYSUCC	Female	53	Rectum	III	2914.0	13.6	--	--
SYSUCC-324	SYSUCC	Female	19	Rectum	IV	2605.7	17.1	--	--
SYSUCC-325	SYSUCC	Female	67	Rectum	IV	3111.1	10.1	--	--
SYSUCC-326	SYSUCC	Female	60	Rectum	IV	2754.9	11.1	--	--
SYSUCC-327	SYSUCC	Male	58	Rectum	III	2628.5	8.8	--	--
SYSUCC-328	SYSUCC	Male	49	Colon	III	2357.7	4.1	--	--
SYSUCC-329	SYSUCC	Male	61	Colon	III	2678.4	3.2	--	--
SYSUCC-330	SYSUCC	Male	49	Colon	IV	3301.8	8.3	--	--
SYSUCC-331	SYSUCC	Female	45	Colon	IV	3169.4	2.3	--	--
SYSUCC-332	SYSUCC	Male	73	Rectum	IV	3031.2	5.7	--	--
SYSUCC-333	SYSUCC	Male	65	Rectum	III	2609.7	2.0	--	--
SYSUCC-334	SYSUCC	Female	39	Rectum	III	2661.3	6.9	--	--
SYSUCC-335	SYSUCC	Male	61	Colon	IV	2865.0	2.9	--	--
SYSUCC-336	SYSUCC	Male	62	Rectum	IV	3805.7	1.8	--	--
SYSUCC-337	SYSUCC	Female	55	Colon	III	2568.8	14.5	--	--
SYSUCC-338	SYSUCC	Male	49	Rectum	IV	3444.6	5.8	--	--
SYSUCC-339	SYSUCC	Male	69	Rectum	I	1438.6	12.0	--	--
SYSUCC-340	SYSUCC	Female	69	Colon	III	2448.8	5.3	--	--
SYSUCC-341	SYSUCC	Male	64	Colon	I	1188.3	9.2	--	--
SYSUCC-342	SYSUCC	Female	45	Colon	III	2830.4	29.7	--	--
SYSUCC-343	SYSUCC	Male	35	Colon	III	3089.4	3.4	--	--
SYSUCC-344	SYSUCC	Male	59	Colon	IV	3121.6	1.1	--	--
SYSUCC-345	SYSUCC	Female	59	Rectum	I	1152.4	13.0	--	--
SYSUCC-346	SYSUCC	Male	56	Colon	III	1251.6	5.1	--	--
SYSUCC-347	SYSUCC	Male	73	Colon	III	3193.8	2.9	--	--
SYSUCC-348	SYSUCC	Male	63	Rectum	III	2769.2	13.7	--	--
SYSUCC-349	SYSUCC	Male	64	Rectum	III	2749.3	11.7	--	--
SYSUCC-350	SYSUCC	Male	75	Rectum	IV	2914.6	1.4	--	--
SYSUCC-351	SYSUCC	Male	57	Rectum	III	1217.3	15.0	--	--
SYSUCC-352	SYSUCC	Male	48	Rectum	IV	6514.3	8.1	--	--
SYSUCC-353	SYSUCC	Male	37	Rectum	III	3920.1	5.2	--	--
SYSUCC-354	SYSUCC	Male	64	Rectum	III	3528.7	6.8	--	--
SYSUCC-355	SYSUCC	Female	68	Colon	I	1377.2	2.0	--	--
SYSUCC-356	SYSUCC	Male	62	Rectum	III	3126.0	2.5	--	--
SYSUCC-357	SYSUCC	Male	77	Rectum	III	3197.1	3.4	--	--
SYSUCC-358	SYSUCC	Female	45	Rectum	I	1282.4	5.7	--	--

SYSUCC-359	SYSUCC	Female	74	Colon	I	1172.9	5.6	--	--
SYSUCC-360	SYSUCC	Female	68	Colon	III	2760.1	5.1	--	--
SYSUCC-361	SYSUCC	Female	46	Rectum	III	1723.7	3.3	--	--
SYSUCC-362	SYSUCC	Female	57	Colon	II	2053.9	19.8	--	--
SYSUCC-363	SYSUCC	Female	42	Colon	III	4245.7	18.4	--	--
SYSUCC-364	SYSUCC	Male	49	Colon	I	1292.8	12.1	--	--
SYSUCC-365	SYSUCC	Female	34	Rectum	III	1703.6	3.3	--	--
SYSUCC-366	SYSUCC	Male	62	Rectum	II	2196.3	8.7	--	--
SYSUCC-367	SYSUCC	Male	43	Rectum	III	2943.7	10.4	--	--
SYSUCC-368	SYSUCC	Male	54	Colon	II	2038.1	3.9	--	--
SYSUCC-369	SYSUCC	Male	57	Rectum	III	2628.8	9.7	--	--
SYSUCC-370	SYSUCC	Female	49	Colon	III	4263.9	1.0	--	--
SYSUCC-371	SYSUCC	Male	31	Colon	III	5164.2	22.9	--	--
SYSUCC-372	SYSUCC	Male	58	Rectum	I	1339.4	6.8	--	--
SYSUCC-373	SYSUCC	Female	44	Rectum	I	1220.8	23.4	--	--
SYSUCC-374	SYSUCC	Female	50	Rectum	III	2861.7	23.6	--	--
SYSUCC-375	SYSUCC	Male	71	Rectum	II	2262.4	13.3	--	--
SYSUCC-376	SYSUCC	Male	44	Rectum	III	2414.5	12.5	--	--
SYSUCC-377	SYSUCC	Female	65	Rectum	I	1406.0	4.1	--	--
SYSUCC-378	SYSUCC	Female	51	Colon	III	3001.0	3.1	--	--
SYSUCC-379	SYSUCC	Male	57	Rectum	II	1597.6	7.4	--	--
SYSUCC-380	SYSUCC	Female	70	Colon	III	1942.9	5.6	--	--
SYSUCC-381	SYSUCC	Male	27	Colon	III	2831.3	5.7	--	--
SYSUCC-382	SYSUCC	Female	53	Colon	III	2333.2	0.9	--	--
SYSUCC-383	SYSUCC	Male	72	Rectum	III	3902.8	6.8	--	--
SYSUCC-384	SYSUCC	Male	67	Colon	III	2557.6	7.5	--	--
SYSUCC-385	SYSUCC	Male	43	Colon	I	870.1	15.0	--	--
SYSUCC-386	SYSUCC	Male	66	Rectum	II	2047.3	5.1	--	--
SYSUCC-387	SYSUCC	Female	60	Colon	III	2866.7	5.0	--	--
SYSUCC-388	SYSUCC	Male	56	Colon	IV	3378.8	7.4	--	--
SYSUCC-389	SYSUCC	Male	66	Rectum	III	2473.1	4.6	--	--
SYSUCC-390	SYSUCC	Female	46	Colon	II	1765.7	3.5	--	--
SYSUCC-391	SYSUCC	Male	39	Rectum	III	3602.6	4.1	--	--
SYSUCC-392	SYSUCC	Male	72	Rectum	III	2753.9	7.3	--	--
SYSUCC-393	SYSUCC	Female	74	Colon	III	2497.4	3.4	--	--
SYSUCC-394	SYSUCC	Female	63	Rectum	II	2023.6	9.1	--	--
SYSUCC-395	SYSUCC	Female	72	Colon	III	1221.4	9.1	--	--
SYSUCC-396	SYSUCC	Male	62	Colon	II	1905.1	5.7	--	--
SYSUCC-397	SYSUCC	Male	67	Rectum	III	1684.6	21.0	--	--
SYSUCC-398	SYSUCC	Female	35	Rectum	III	3045.1	6.7	--	--
SYSUCC-399	SYSUCC	Male	59	Colon	III	1949.2	7.7	--	--
SYSUCC-400	SYSUCC	Female	64	Rectum	III	2536.9	2.5	--	--
SYSUCC-401	SYSUCC	Male	56	Colon	II	1646.6	1.2	--	--
SYSUCC-402	SYSUCC	Female	39	Colon	II	1650.9	3.5	--	--

SYSUCC-403	SYSUCC	Female	60	Rectum	III	3671.6	3.4	--	--
SYSUCC-404	SYSUCC	Male	65	Rectum	III	2599.6	6.0	--	--
SYSUCC-405	SYSUCC	Male	39	Colon	III	4512.3	9.5	--	--
SYSUCC-406	SYSUCC	Male	55	Colon	III	2652.0	9.2	--	--
SYSUCC-407	SYSUCC	Male	59	Rectum	III	2393.9	2.3	--	--
SYSUCC-408	SYSUCC	Male	59	Rectum	II	1685.8	9.8	--	--
SYSUCC-409	SYSUCC	Male	53	Rectum	III	2530.8	1.3	--	--
SYSUCC-410	SYSUCC	Male	79	Rectum	III	1491.0	6.3	--	--
SYSUCC-411	SYSUCC	Male	57	Colon	II	1164.9	5.2	--	--
SYSUCC-412	SYSUCC	Male	68	Rectum	II	1987.7	6.1	--	--
SYSUCC-413	SYSUCC	Female	49	Colon	III	2818.6	3.4	--	--
SYSUCC-414	SYSUCC	Male	53	Rectum	III	1475.3	3.0	--	--
SYSUCC-415	SYSUCC	Female	48	Rectum	III	2798.2	4.7	--	--
SYSUCC-416	SYSUCC	Female	61	Colon	II	1525.7	23.6	--	--
SYSUCC-417	SYSUCC	Female	74	Rectum	I	1087.0	7.0	--	--
SYSUCC-418	SYSUCC	Male	62	Rectum	II	2205.9	5.0	--	--
SYSUCC-419	SYSUCC	Female	59	Rectum	II	2106.0	17.6	--	--
SYSUCC-420	SYSUCC	Male	66	Rectum	II	2205.7	8.3	--	--
SYSUCC-421	SYSUCC	Female	47	Rectum	III	1863.4	6.8	--	--
SYSUCC-422	SYSUCC	Female	57	Colon	III	3133.6	1.0	--	--
SYSUCC-423	SYSUCC	Female	56	Colon	III	2673.9	29.9	--	--
SYSUCC-424	SYSUCC	Male	54	Colon	II	2226.9	5.9	--	--
SYSUCC-425	SYSUCC	Female	70	Colon	II	1767.9	3.0	--	--
SYSUCC-426	SYSUCC	Male	62	Rectum	III	2898.5	4.9	--	--
SYSUCC-427	SYSUCC	Male	52	Rectum	II	2069.2	2.4	--	--
SYSUCC-428	SYSUCC	Male	57	Colon	II	901.0	4.9	--	--
SYSUCC-429	SYSUCC	Female	44	Rectum	III	2540.6	2.1	--	--
SYSUCC-430	SYSUCC	Male	33	Rectum	III	2640.2	7.9	--	--
SYSUCC-431	SYSUCC	Male	34	Colon	II	2036.7	6.6	--	--
SYSUCC-432	SYSUCC	Male	63	Colon	III	3086.0	9.7	--	--
SYSUCC-433	SYSUCC	Male	71	Colon	III	2289.5	16.6	--	--
SYSUCC-434	SYSUCC	Female	57	Rectum	III	1718.4	7.3	--	--
SYSUCC-435	SYSUCC	Male	60	Rectum	III	2815.2	4.4	--	--
SYSUCC-436	SYSUCC	Female	54	Colon	III	2774.4	12.4	--	--
SYSUCC-437	SYSUCC	Female	70	Colon	III	2868.8	7.8	--	--
SYSUCC-438	SYSUCC	Female	54	Colon	II	2326.6	12.8	--	--
SYSUCC-439	SYSUCC	Female	70	Colon	III	3727.2	0.8	--	--
SYSUCC-440	SYSUCC	Male	33	Colon	II	1710.0	12.0	--	--
SYSUCC-441	SYSUCC	Female	55	Colon	II	2094.6	7.5	--	--
SYSUCC-442	SYSUCC	Male	47	Colon	II	1931.6	1.6	--	--
SYSUCC-443	SYSUCC	Female	38	Colon	III	2183.6	13.1	--	--
SYSUCC-444	SYSUCC	Female	62	Colon	II	2225.0	5.0	--	--
SYSUCC-445	SYSUCC	Male	51	Rectum	III	2820.7	0.9	--	--
SYSUCC-446	SYSUCC	Female	59	Colon	II	2047.8	4.9	--	--

SYSUCC-447	SYSUCC	Female	56	Colon	III	3419.9	9.4	--	--
SYSUCC-448	SYSUCC	Male	63	Rectum	III	2494.0	4.2	--	--
SYSUCC-449	SYSUCC	Male	48	Rectum	III	2824.3	9.2	--	--
SYSUCC-450	SYSUCC	Male	29	Rectum	III	2604.2	3.5	--	--
SYSUCC-451	SYSUCC	Male	61	Colon	III	2223.0	4.3	--	--
SYSUCC-452	SYSUCC	Male	46	Colon	II	2239.5	15.5	--	--
SYSUCC-453	SYSUCC	Female	38	Colon	IV	3073.4	9.8	--	--
SYSUCC-454	SYSUCC	Female	54	Colon	III	1994.5	6.9	--	--
SYSUCC-455	SYSUCC	Male	75	Colon	III	1895.9	16.2	--	--
SYSUCC-456	SYSUCC	Male	40	Rectum	II	1793.1	8.8	--	--
SYSUCC-457	SYSUCC	Female	47	Rectum	II	1884.7	3.9	--	--
SYSUCC-458	SYSUCC	Male	78	Colon	II	1837.2	4.0	--	--
SYSUCC-459	SYSUCC	Male	60	Rectum	II	2176.5	4.6	--	--
SYSUCC-460	SYSUCC	Female	54	Colon	III	1887.8	8.0	--	--
SYSUCC-461	SYSUCC	Female	54	Colon	III	4104.2	7.3	--	--
SYSUCC-462	SYSUCC	Male	65	Colon	II	2750.7	6.2	--	--
SYSUCC-463	SYSUCC	Male	53	Colon	II	2228.9	7.1	--	--
SYSUCC-464	SYSUCC	Female	55	Colon	III	2937.2	3.7	--	--
SYSUCC-465	SYSUCC	Male	69	Rectum	II	2095.7	6.2	--	--
SYSUCC-466	SYSUCC	Female	62	Rectum	III	2265.1	1.6	--	--
SYSUCC-467	SYSUCC	Female	52	Rectum	I	1224.9	5.5	--	--
SYSUCC-468	SYSUCC	Female	63	Rectum	II	1904.6	5.6	--	--
SYSUCC-469	SYSUCC	Male	52	Colon	II	1835.4	3.5	--	--
SYSUCC-470	SYSUCC	Male	49	Rectum	II	1750.9	14.9	--	--
SYSUCC-471	SYSUCC	Female	57	Colon	II	1673.2	4.4	--	--
SYSUCC-472	SYSUCC	Male	56	Colon	II	2083.0	10.7	--	--
SYSUCC-473	SYSUCC	Female	31	Colon	III	2718.9	14.1	--	--
SYSUCC-474	SYSUCC	Male	60	Colon	III	2654.6	20.9	--	--
SYSUCC-475	SYSUCC	Female	59	Colon	II	1851.1	7.8	--	--
SYSUCC-476	SYSUCC	Female	61	Rectum	II	1622.2	1.3	--	--
SYSUCC-477	SYSUCC	Female	65	Colon	III	1922.5	15.1	--	--
SYSUCC-478	SYSUCC	Male	48	Colon	II	1649.2	8.8	--	--
SYSUCC-479	SYSUCC	Male	41	Colon	II	2013.4	8.2	--	--
SYSUCC-480	SYSUCC	Female	47	Colon	III	3153.6	12.3	--	--
SYSUCC-481	SYSUCC	Male	57	Colon	II	1542.2	8.7	--	--
SYSUCC-482	SYSUCC	Female	57	Rectum	II	1969.3	8.8	--	--
SYSUCC-483	SYSUCC	Female	60	Rectum	III	3054.8	4.9	--	--
SYSUCC-484	SYSUCC	Female	55	Rectum	III	2393.7	19.6	--	--
SYSUCC-485	SYSUCC	Male	42	Rectum	III	2798.8	11.8	--	--
SYSUCC-486	SYSUCC	Female	29	Rectum	II	2018.9	17.7	--	--
SYSUCC-487	SYSUCC	Female	42	Rectum	II	1507.5	0.1	--	--
SYSUCC-488	SYSUCC	Male	56	Colon	III	2001.4	8.5	--	--
SYSUCC-489	SYSUCC	Female	50	Rectum	II	2091.4	5.6	--	--
SYSUCC-490	SYSUCC	Male	37	Colon	III	2965.0	5.7	--	--

SYSUCC-491	SYSUCC	Male	48	Rectum	II	2044.6	9.1	--	--
SYSUCC-492	SYSUCC	Female	59	Rectum	III	2856.7	2.1	--	--
SYSUCC-493	SYSUCC	Female	71	Colon	II	2331.4	7.3	--	--
SYSUCC-494	SYSUCC	Male	58	Rectum	IV	3074.1	4.7	--	--
SYSUCC-495	SYSUCC	Male	76	Colon	IV	3133.8	6.9	--	--
SYSUCC-496	SYSUCC	Female	53	Rectum	III	2638.6	1.5	--	--
SYSUCC-497	SYSUCC	Male	52	Colon	III	2344.7	4.8	--	--
SYSUCC-498	SYSUCC	Male	63	Colon	II	1675.5	8.6	--	--
SYSUCC-499	SYSUCC	Male	43	Colon	III	2728.9	2.8	--	--
SYSUCC-500	SYSUCC	Male	62	Rectum	II	1864.7	1.1	--	--
SYSUCC-501	SYSUCC	Female	70	Colon	III	2144.8	7.3	--	--
SYSUCC-502	SYSUCC	Male	63	Colon	II	2030.6	5.6	--	--
SYSUCC-503	SYSUCC	Male	51	Colon	III	2547.3	6.1	--	--
SYSUCC-504	SYSUCC	Male	66	Rectum	III	2825.9	7.7	--	--
SYSUCC-505	SYSUCC	Male	46	Colon	II	1863.9	9.1	--	--
SYSUCC-506	SYSUCC	Male	35	Rectum	II	2224.5	2.9	--	--
SYSUCC-507	SYSUCC	Male	33	Rectum	II	1611.1	2.6	--	--
SYSUCC-508	SYSUCC	Male	83	Rectum	III	3346.4	11.5	--	--
SYSUCC-509	SYSUCC	Male	73	Colon	II	1719.4	7.9	--	--
SYSUCC-510	SYSUCC	Female	54	Colon	I	1325.3	1.6	--	--
SYSUCC-511	SYSUCC	Male	54	Colon	II	2137.8	7.8	--	--
SYSUCC-512	SYSUCC	Female	60	Rectum	III	3325.7	14.6	--	--
SYSUCC-513	SYSUCC	Male	63	Rectum	I	1424.1	6.8	--	--
SYSUCC-514	SYSUCC	Female	56	Rectum	III	3277.5	7.4	--	--
SYSUCC-515	SYSUCC	Male	58	Rectum	III	1966.9	3.6	--	--
SYSUCC-516	SYSUCC	Male	63	Colon	II	1884.0	1.5	--	--
SYSUCC-517	SYSUCC	Male	50	Colon	III	1952.2	5.1	--	--
SYSUCC-518	SYSUCC	Female	54	Rectum	III	2518.8	4.5	--	--
SYSUCC-519	SYSUCC	Male	60	Colon	III	2212.0	6.6	--	--
SYSUCC-520	SYSUCC	Female	70	Colon	II	1133.2	3.7	--	--
SYSUCC-521	SYSUCC	Male	67	Colon	II	1811.6	1.8	--	--
SYSUCC-522	SYSUCC	Male	42	Colon	I	1479.4	1.7	--	--
SYSUCC-523	SYSUCC	Male	41	Rectum	III	2786.4	22.8	--	--
SYSUCC-524	SYSUCC	Male	55	Colon	II	1714.3	2.4	--	--
SYSUCC-525	SYSUCC	Female	29	Colon	II	1834.9	28.3	--	--
SYSUCC-526	SYSUCC	Male	59	Rectum	I	1381.3	10.3	--	--
SYSUCC-527	SYSUCC	Male	74	Rectum	II	1108.1	7.9	--	--
SYSUCC-528	SYSUCC	Female	64	Rectum	III	2611.1	9.5	--	--
SYSUCC-529	SYSUCC	Female	51	Rectum	II	1769.1	14.1	--	--
SYSUCC-530	SYSUCC	Male	34	Colon	I	1283.5	3.3	--	--
SYSUCC-531	SYSUCC	Male	66	Rectum	III	4035.1	10.4	--	--
SYSUCC-532	SYSUCC	Male	54	Rectum	III	4673.6	5.9	--	--
SYSUCC-533	SYSUCC	Male	57	Colon	III	2799.5	4.1	--	--
SYSUCC-534	SYSUCC	Male	56	Colon	IV	3290.9	5.1	--	--

SYSUCC-535	SYSUCC	Female	38	Rectum	IV	3358.9	6.2	--	--
SYSUCC-536	SYSUCC	Male	58	Colon	III	1977.8	8.5	--	--
SYSUCC-537	SYSUCC	Female	40	Colon	III	3148.1	1.5	--	--
SYSUCC-538	SYSUCC	Male	47	Colon	IV	3465.3	15.0	--	--
SYSUCC-539	SYSUCC	Male	53	Rectum	III	2234.5	7.3	--	--
SYSUCC-540	SYSUCC	Male	51	Rectum	III	2367.1	4.9	--	--
SYSUCC-541	SYSUCC	Female	41	Colon	III	1979.9	4.1	--	--
SYSUCC-542	SYSUCC	Male	65	Rectum	III	3005.0	6.0	--	--
SYSUCC-543	SYSUCC	Female	56	Colon	III	2430.0	13.8	--	--
SYSUCC-544	SYSUCC	Male	66	Rectum	III	3415.3	2.7	--	--
SYSUCC-545	SYSUCC	Male	59	Colon	III	2649.7	16.5	--	--
SYSUCC-546	SYSUCC	Male	70	Colon	III	2344.2	2.6	--	--
SYSUCC-547	SYSUCC	Female	70	Rectum	III	3256.4	7.6	--	--
SYSUCC-548	SYSUCC	Male	45	Colon	III	2635.2	7.9	--	--
SYSUCC-549	SYSUCC	Female	77	Rectum	III	2739.8	5.9	--	--
SYSUCC-550	SYSUCC	Male	64	Rectum	IV	3641.2	11.0	--	--
SYSUCC-551	SYSUCC	Female	46	Colon	IV	3615.5	3.1	--	--
SYSUCC-552	SYSUCC	Female	67	Rectum	IV	3235.4	26.4	--	--
SYSUCC-553	SYSUCC	Female	31	Rectum	IV	2975.7	15.5	--	--
SYSUCC-554	SYSUCC	Male	65	Colon	IV	3228.8	2.0	--	--
SYSUCC-555	SYSUCC	Female	46	Rectum	IV	3025.6	10.7	--	--
SYSUCC-556	SYSUCC	Male	56	Rectum	IV	3163.0	5.4	--	--
SYSUCC-557	SYSUCC	Female	62	Colon	IV	3103.7	7.8	--	--
SYSUCC-558	SYSUCC	Female	53	Colon	IV	3988.6	8.6	--	--
SYSUCC-559	SYSUCC	Male	46	Rectum	IV	3787.7	4.2	--	--
SYSUCC-560	SYSUCC	Male	59	Colon	IV	3165.5	7.6	--	--
SYSUCC-561	SYSUCC	Male	55	Rectum	IV	3958.9	5.2	--	--

[1]SYSUCC, Sun Yat-sen University Cancer Center (Guangzhou, China); CHCAMS, Cancer Hospital of Chinese Academy of Medical Sciences (Beijing, China)

[2]Mean of triplicate measurements and the standard error of mean (SEM). Serum piR-54265 level was determined from blood of individuals at diagnosis prior to any treatments or after tumor resection in 7 days.

Table S3. General characteristics of participants in the nested case-control analysis derived from the Dongfeng-Tongji prospective cohort

Variable[a]	Cases (*N*=307)	Controls (*N*=614)[c]
Age (year, mean ± SD)	66.6 ± 7.2	66.5 ± 7.0
Sex		
Male, *N* (%)	170 (55.4)	340 (55.4)
Female, *N* (%)	137 (44.6)	274 (44.6)
Smoking status		
Smoker, *N* (%)	110 (35.8)	232 (37.9)
Non-smoker, *N* (%)	195 (63.5)	380 (62.1)
Missing	2 (0.7)	0 (0)
Drinking status		
Drinker, *N* (%)	88 (28.8)	187 (30.6)
Non-drinker, *N* (%)	217 (70.9)	425 (69.4)
Missing	1 (0.3)	0 (0)
Regular physical activity		
Yes, *N* (%)	238 (77.5)	473 (77.0)
No, *N* (%)	25 (8.2)	68 (11.1)
Missing, *N* (%)	44 (14.3)	73 (11.9)
Family history of cancer		
Yes, *N* (%)	6 (1.9)	24 (3.9)
No, *N* (%)	259 (84.4)	512 (83.4)
Missing, *N* (%)	42 (13.7)	78 (12.7)
BMI (kg/m^2, mean ± SD)[b]	25.0 ± 3.5	23.8 ± 3.3
CEA (ng/mL, IQR)	1.73 (1.09, 2.81)	1.65 (0.94, 2.46)
CA125 (Unit/mL, IQR)	3.89 (2.35, 7.10)	5.10 (2.01, 9.30)
CA19-9 (Unit/mL, IQR)	7.05 (3.85, 14.78)	7.39 (4.16, 14.05)

Abbreviations: BMI, body mass index; IQR, inter-quartile range; SD, standard derivation.
[a]Normally and non-normally distributed variables were presented as mean ± SD and median (IQR), respectively. For categorical variables, values were presented as number (percentage).
[b]Eleven cases and 8 controls had missing BMI data.
[c]One control had missing data of CEA, CA125 and CA19-9.

Table S4. The accumulated numbers of incident CRC cases diagnosed by years in the prospective Dongfeng-Tongji cohort

Diagnosed before the date	Cases enrolled between 2008 to 2010 (*N*=27009)	Cases enrolled in 2013 (*N*=14120)	Subtotal
2009.12.31	16	--	16
2010.12.31	36	--	36
2011.12.31	66	--	66
2012.12.31	106	--	106
2013.12.31	154	14	168
2014.12.31	196	28	224
2015.12.31	237	50	287
2016.12.31	277	50	327
2017.12.31	277	54	331
2018.12.31	280 (17)[a]	57 (13)[a]	337 (30)[a]

[a]Number in the parenthesis indicates missing serum specimens for some reasons.

Table S5. Clinical features and serum piR-54265 levels of the participants in prospective case-control analysis from Dongfeng-Tongji cohort

Sample ID	Subjuct[1]	Diagnosis	Diagnosed date	Last follow-up date	Time between enrollment and diagnosis (year)	Age (year)	Sex	CEA (ng/mL)	CA125 (U/mL)	CA19-9 (U/mL)	BMI	Smoking Status[2]	Drinking status[3]	Regular physical activity[4]	Family cancer history	piR-54265[5]	
																Mean copy/μL	SEM
1	Case	CC	2014-07-20	2014-07-20	1.0	75	Female	0.81	2.57	12.89	27.87	Yes	Yes	Missing	Missing	2479.6	6.6
2	Case	CC	2015-01-13	2015-01-13	1.1	57	Female	2.11	2.75	31.36	21.93	No	No	Yes	No	1556.6	2.1
3	Case	CC	2011-09-15	2011-09-15	2.9	62	Male	2.24	0.21	4.77	24.62	Yes	No	Yes	No	2227.9	9.7
4	Case	CC	2012-10-10	2012-10-10	3.7	75	Male	1.29	26.50	7.30	24.50	Yes	No	Yes	No	400.0	5.5
5	Case	CC	2013-11-10	2013-11-10	4.8	72	Male	3.57	15.10	2.00	22.73	No	No	Yes	No	475.6	7.3
6	Case	CC	2016-11-16	2016-11-16	3.5	77	Female	2.01	3.07	17.21	20.50	No	No	Missing	No	1469.8	5.2
7	Case	CC	2013-11-10	2013-11-10	5.1	64	Male	0.50	4.27	11.66	23.29	No	No	Yes	No	1536.9	17.0
8	Case	CC	2016-03-27	2016-03-27	2.8	66	Male	12.27	4.16	20.02	23.52	No	No	Missing	Missing	2769.1	11.7
9	Case	CC	2012-08-24	2012-08-24	3.5	70	Female	3.64	11.40	7.75	30.00	No	No	Yes	No	187.6	8.4
10	Case	CC	2016-05-03	2016-05-03	2.6	82	Male	1.27	2.68	3.9	27.42	Yes	No	Yes	No	1305.1	7.5
11	Case	CC	2016-08-02	2016-08-02	2.8	58	Male	1.33	2.59	4.89	29.46	Yes	No	Yes	No	1103.0	1.8
12	Case	CC	2013-07-06	2013-07-06	0.6	61	Male	1.00	3.25	29.7	24.23	No	Yes	Yes	No	1206.7	4.4
13	Case	CC	2016-05-30	2016-05-30	2.5	68	Male	0.85	2.47	2.39	28.06	Yes	Yes	Yes	No	668.8	8.3
14	Case	CC	2016-12-03	2016-12-03	3.1	71	Female	1.27	3.21	4.41	27.49	No	No	Yes	No	1377.9	1.7
15	Case	CC	2013-11-10	2013-11-10	0.0	64	Male	2.10	12.62	13.88	21.01	Yes	Yes	Yes	Yes	1627.3	3.1
16	Case	CC	2011-01-07	2011-01-07	2.2	74	Male	3.82	5.30	46.71	32.32	No	No	Yes	No	1138.6	10.5
17	Case	CC	2010-03-04	2010-03-04	1.0	65	Male	2.34	0.72	26.38	18.96	Yes	Yes	Yes	No	1750.6	5.5
18	Case	CC	2013-12-12	2013-12-12	0.0	63	Male	1.99	5.37	5.55	24.84	Yes	No	Yes	No	1453.3	5.3
19	Case	CC	2009-09-13	2009-09-13	0.9	55	Male	6.91	10.90	2.00	25.90	Yes	No	Yes	No	1311.0	10.3
20	Case	CC	2012-01-06	2012-01-06	3.2	70	Male	0.50	0.72	17.55	28.52	No	No	Yes	No	1092.8	8.3

21	Case	CC	2013-11-10	2013-11-10	5.0	71	Female	4.01	3.26	12.80	19.04	No	No	No	No	1165.7	1.9
22	Case	CC	2015-08-18	2015-08-18	6.4	77	Female	6.14	0.00	19.91	Missing	No	Yes	Yes	No	1435.7	13.0
23	Case	CC	2015-12-19	2015-12-19	2.1	71	Male	1.28	7.89	2.32	27.61	Yes	Yes	Yes	No	1576.5	2.6
24	Case	CC	2012-10-20	2012-10-20	3.9	53	Female	0.74	0.36	19.76	34.63	No	No	Yes	No	2409.7	18.0
25	Case	CC	2011-05-04	2011-05-04	2.4	61	Male	1.02	10.40	8.76	31.23	Yes	Yes	Yes	No	370.0	6.6
26	Case	CC	2009-11-13	2009-11-13	0.7	71	Male	2.15	12.70	4.13	34.16	Yes	No	Yes	No	1175.5	8.6
27	Case	CC	2012-12-26	2012-12-26	3.8	67	Male	1.23	6.70	2.40	23.81	No	No	Yes	No	1066.5	6.3
28	Case	CC	2011-03-24	2011-03-24	2.4	66	Male	1.97	10.50	8.34	26.77	No	Yes	Yes	No	626.2	8.4
29	Case	CC	2012-11-05	2012-11-05	4.1	79	Female	2.00	3.84	0.01	21.49	No	No	Yes	No	215.8	9.8
30	Case	CC	2011-04-02	2011-04-02	2.4	77	Female	1.31	12.80	7.13	28.40	No	No	No	No	1575.7	6.8
31	Case	CC	2013-06-13	2013-06-13	0.6	72	Male	5.50	13.59	30.04	26.47	Yes	No	Yes	No	1638.3	3.5
32	Case	CC	2013-11-08	2013-11-08	0.9	67	Male	1.58	2.93	10.19	22.41	Yes	Yes	No	No	1404.5	8.5
33	Case	CC	2015-04-20	2015-04-20	1.9	63	Male	2.05	4.16	7.8	25.39	No	No	Missing	Missing	5393.8	7.7
34	Case	CC	2013-11-10	2013-11-10	5.0	78	Male	2.73	6.50	7.18	24.24	No	No	Yes	No	1404.9	17.2
35	Case	CC	2009-07-28	2009-07-28	0.7	61	Female	3.75	29.90	26.68	21.70	No	No	Yes	No	1169.9	10.7
36	Case	CC	2009-12-16	2009-12-16	1.2	61	Female	0.33	2.98	10.53	30.41	Missing	No	Yes	No	2025.0	12.4
37	Case	CC	2011-01-13	2011-01-13	2.3	61	Male	1.35	3.40	13.76	23.34	No	No	Yes	No	1533.8	4.1
38	Case	CC	2010-01-19	2010-01-19	1.3	62	Female	2.03	3.32	14.41	26.71	No	No	Yes	No	236.9	4.8
39	Case	CC	2015-12-25	2015-12-25	2.4	54	Female	0.90	3.22	3.12	27.41	No	Yes	Missing	Missing	2571.7	7.2
40	Case	CC	2012-04-25	2012-04-25	3.3	72	Female	1.71	8.80	6.39	25.85	No	No	Yes	No	934.1	2.4
41	Case	CC	2011-08-01	2011-08-01	2.8	63	Male	0.47	2.47	2.13	26.22	Yes	No	Yes	No	1240.5	3.8
42	Case	CC	2016-09-21	2016-09-21	3.0	51	Female	0.47	2.59	5.64	23.64	No	No	Yes	No	542.4	4.6
43	Case	CC	2013-10-13	2013-10-13	5.0	63	Male	3.07	13.90	8.78	22.53	No	No	Yes	No	1587.8	10.5
44	Case	CC	2012-05-05	2012-05-05	3.4	59	Female	2.78	4.90	23.77	29.32	No	No	Yes	No	1252.9	10.5
45	Case	CC	2011-03-21	2011-03-21	2.1	57	Female	0.00	0.00	0.00	24.69	No	No	Yes	No	1705.4	14.2
46	Case	CC	2010-07-13	2010-07-13	1.6	59	Female	0.21	0.66	7.05	23.83	No	No	Yes	No	352.5	4.6
47	Case	CC	2016-09-06	2016-09-06	3.0	81	Female	0.73	1.46	0.00	30.73	No	No	Yes	No	2274.3	3.6
48	Case	CC	2010-03-31	2010-03-31	1.3	77	Female	7.48	7.90	6.68	21.57	No	No	Yes	No	1149.8	5.9

49	Case	CC	2013-10-18	2013-10-18	0.8	61	Female	3.21	2.96	5.59	33.30	No	No	Yes	No	718.0	4.3
50	Case	CC	2013-03-19	2013-03-19	4.2	61	Male	4.21	2.70	16.28	25.71	No	No	Yes	No	435.6	16.6
51	Case	CC	2015-11-07	2015-11-07	6.8	63	Male	8.27	8.30	5.69	25.97	Yes	Yes	Yes	No	2285.1	18.3
52	Case	CC	2013-10-04	2013-10-04	4.9	57	Female	1.18	0.59	31.91	19.95	No	No	Yes	No	1154.5	5.6
53	Case	CC	2013-02-18	2013-02-18	4.4	59	Male	0.80	0.20	5.20	23.15	Yes	No	Yes	No	1246.7	13.8
54	Case	CC	2012-02-28	2012-02-28	3.3	78	Male	2.34	8.60	13.02	24.98	Yes	Yes	Yes	No	1584.3	5.4
55	Case	CC	2016-01-11	2016-01-11	1.9	83	Female	0.87	15.09	3.63	30.64	No	No	Yes	No	2136.3	2.7
56	Case	CC	2016-08-03	2016-08-03	2.4	80	Male	2.13	6.35	10.23	29.33	Yes	Yes	Yes	No	1494.2	7.2
57	Case	CC	2016-06-18	2016-06-18	2.5	74	Male	1.21	2.19	4.52	25.15	Yes	Yes	Yes	No	1119.9	11.3
58	Case	CC	2009-07-31	2009-07-31	0.6	70	Male	4.11	8.60	4.48	21.09	No	No	Yes	No	3369.3	6.8
59	Case	CC	2012-07-30	2012-07-30	3.6	61	Female	3.00	2.90	3.99	28.72	No	No	Yes	No	376.3	6.6
60	Case	CC	2015-11-30	2015-11-30	2.1	61	Female	0.61	1.64	4.97	28.95	No	No	Yes	No	1118.4	11.1
61	Case	CC	2009-11-17	2009-11-17	0.9	62	Male	8.17	5.50	11.29	28.88	Yes	Yes	Yes	No	5398.0	8.3
62	Case	CC	2013-11-13	2013-11-13	0.7	75	Male	1089.27	6.20	2120.69	23.25	Yes	No	Yes	No	1492.2	6.1
63	Case	CC	2012-11-03	2012-11-03	3.8	70	Female	1.07	5.20	4.96	23.07	No	No	Yes	No	1705.7	9.6
64	Case	CC	2013-07-31	2013-07-31	4.7	63	Female	2.73	2.18	20.15	28.67	No	Yes	Yes	No	2076.7	5.8
65	Case	CC	2011-07-10	2011-07-10	2.6	80	Male	2.33	9.60	20.98	22.03	No	Yes	Yes	No	309.5	3.1
66	Case	CC	2011-02-15	2011-02-15	2.3	74	Male	1.31	0.91	22.43	24.57	No	Yes	Yes	No	1861.1	8.5
67	Case	CC	2013-09-18	2013-09-18	4.9	64	Male	0.12	0.01	5.51	25.07	No	Yes	Yes	No	1322.9	6.2
68	Case	CC	2016-11-07	2016-11-07	2.9	54	Female	1.30	1.44	25.15	Missing	No	No	No	No	603.3	3.7
69	Case	CC	2013-07-23	2013-07-23	4.6	66	Male	3.56	6.60	2.00	24.98	No	Yes	Yes	No	835.9	6.5
70	Case	CC	2012-05-22	2012-05-22	3.2	65	Female	1.71	9.70	2.17	23.07	No	No	Yes	No	1408.6	7.9
71	Case	CC	2016-03-03	2016-03-03	2.7	80	Male	10.15	2.38	3.11	18.42	Yes	Yes	Missing	Missing	1303.5	5.3
72	Case	CC	2010-09-28	2010-09-28	1.9	76	Male	2.38	2.76	0.02	27.72	No	No	Yes	No	838.4	17.5
73	Case	CC	2011-04-09	2011-04-09	2.4	59	Female	0.00	0.00	0.00	26.84	No	No	Yes	No	1360.3	6.6
74	Case	CC	2010-09-16	2010-09-16	1.8	68	Male	2.48	6.74	12.81	Missing	No	No	Yes	No	2042.8	7.8
75	Case	CC	2015-08-25	2015-08-25	1.5	76	Female	1.31	4.76	10.06	19.40	No	No	Yes	No	1199.5	12.3
76	Case	CC	2012-11-01	2012-11-01	3.7	71	Female	2.10	8.50	8.68	28.03	No	No	Yes	No	1375.4	11.9

77	Case	CC	2014-02-15	2014-02-15	0.8	78	Male	1.32	3.26	7.45	24.81	Yes	Yes	Missing	Missing	1230.9	2.9
78	Case	CC	2014-02-07	2014-02-07	0.2	59	Female	1.16	5.16	2.02	26.64	No	No	No	No	1406.8	3.7
79	Case	CC	2013-11-19	2013-11-19	0.2	71	Male	6.30	5.60	22.42	19.25	No	Yes	Missing	Missing	1706.7	7.8
80	Case	CC	2010-05-28	2010-05-28	1.5	65	Female	2.15	1.16	9.48	21.58	No	No	Yes	No	3650.6	7.0
81	Case	CC	2011-09-26	2011-09-26	3.0	67	Female	0.70	7.17	7.17	26.50	No	No	Yes	No	1484.3	1.6
82	Case	CC	2014-01-13	2014-01-13	0.3	59	Female	2.27	20.24	110.06	25.39	No	No	Yes	No	3273.7	10.4
83	Case	CC	2013-10-10	2013-10-10	5.0	60	Female	0.92	1.95	33.28	33.87	No	No	Yes	No	1849.9	16.8
84	Case	CC	2011-10-06	2011-10-06	2.9	66	Male	1.63	2.41	2.78	22.86	No	No	Yes	No	3044.7	9.0
85	Case	CC	2010-11-21	2010-11-21	2.1	68	Male	8.00	2.50	30.40	25.53	Yes	Yes	Yes	No	4362.5	5.5
86	Case	CC	2013-02-13	2013-02-13	4.3	71	Female	0.26	3.66	21.98	30.36	No	No	Yes	No	1439.4	4.5
87	Case	CC	2012-05-16	2012-05-16	3.6	73	Female	0.90	5.40	6.60	18.38	No	No	Yes	No	2517.2	2.8
88	Case	CC	2016-07-21	2016-07-21	2.6	62	Male	2.38	3.65	8.01	25.80	Yes	No	Yes	No	1663.3	17.5
89	Case	CC	2009-05-14	2009-05-14	0.4	57	Female	1.36	9.00	9.57	23.31	No	No	Yes	No	2152.3	10.0
90	Case	CC	2015-10-23	2015-10-23	6.9	62	Male	3.87	2.35	26.26	29.12	No	No	Yes	No	1625.2	10.3
91	Case	CC	2016-07-21	2016-07-21	7.8	66	Male	0.62	2.24	6.85	24.05	Yes	No	Yes	No	2250.7	16.6
92	Case	CC	2015-03-23	2015-03-23	1.4	69	Female	0.77	2.51	14.59	30.86	No	No	Yes	Yes	2363.1	6.9
93	Case	CC	2014-07-21	2014-07-21	5.7	62	Male	2.13	4.70	6.38	23.38	Yes	Yes	No	No	1392.2	5.6
94	Case	CC	2014-09-28	2014-09-28	0.9	65	Male	1.48	4.08	12.51	26.15	Yes	Yes	Missing	Missing	1386.6	2.2
95	Case	CC	2014-01-09	2014-01-09	5.3	70	Female	0.47	1.19	4.96	22.29	No	No	Yes	No	1981.2	7.4
96	Case	CC	2014-02-08	2014-02-08	0.3	63	Female	3.40	2.88	3.73	25.82	No	No	Yes	No	576.6	9.8
97	Case	CC	2015-05-04	2015-05-04	1.2	65	Male	0.70	4.96	14.69	28.34	No	No	Yes	No	1482.9	14.2
98	Case	CC	2015-02-03	2015-02-03	1.1	72	Male	3.00	6.02	3386.38	26.51	No	No	Yes	No	3006.3	11.4
99	Case	CC	2015-05-11	2015-05-11	6.6	50	Female	3.15	7.90	8.04	22.93	No	No	Yes	No	1319.4	3.7
100	Case	CC	2015-04-23	2015-04-23	6.4	56	Female	3.58	8.50	45.91	23.53	No	No	Yes	No	1617.4	5.9
101	Case	CC	2013-12-18	2013-12-18	5.1	64	Male	1.22	14.50	6.71	23.29	Yes	No	Yes	No	1706.9	4.8
102	Case	CC	2014-09-19	2014-09-19	1.3	62	Male	1.17	2.68	6.61	23.34	Yes	Yes	Missing	Missing	1595.8	14.4
103	Case	CC	2014-11-05	2014-11-05	0.7	62	Male	2.59	9.06	0.00	23.23	Yes	No	Yes	No	525.0	10.2
104	Case	CC	2015-10-01	2015-10-01	2.4	55	Female	0.68	2.44	4.41	23.83	No	Yes	Missing	Missing	3840.7	5.3

105	Case	CC	2015-07-01	2015-07-01	1.7	62	Male	2.82	4.54	19.74	32.53	Yes	No	Yes	No	2909.7	7.7
106	Case	CC	2015-07-21	2015-07-21	1.8	54	Female	0.69	2.16	6.57	25.44	No	Yes	Yes	No	765.5	3.7
107	Case	CC	2015-04-21	2015-04-21	1.6	77	Male	3.49	2.48	0.00	29.96	No	No	Missing	Missing	2535.5	9.1
108	Case	CC	2014-07-01	2014-07-01	1.1	64	Male	6.07	2.22	10.8	28.33	Yes	Yes	Missing	Missing	2627.1	12.1
109	Case	CC	2014-05-29	2014-05-29	5.4	59	Female	2.53	10.00	13.02	22.62	No	No	Yes	Yes	1233.5	5.3
110	Case	CC	2015-07-02	2015-07-02	1.3	74	Male	2.17	5.18	14.48	24.75	No	No	Yes	No	1517.3	5.0
111	Case	CC	2015-03-25	2015-03-25	1.3	63	Male	3.14	4.07	15.87	31.12	No	Yes	Yes	No	704.8	6.2
112	Case	CC	2015-04-27	2015-04-27	2.0	64	Male	2.38	3.59	5.75	23.95	Yes	No	Missing	Missing	780.1	5.8
113	Case	CC	2015-10-08	2015-10-08	2.0	65	Female	0.77	2.24	3.69	23.46	No	No	No	No	2127.2	5.5
114	Case	CC	2015-06-13	2015-06-13	1.7	61	Female	1.35	5.50	28.27	24.77	No	No	Yes	No	1283.6	12.4
115	Case	CC	2014-12-27	2014-12-27	6.0	69	Female	2.44	10.30	27.04	22.64	No	Yes	Yes	No	1222.7	2.5
116	Case	CC	2015-02-25	2015-02-25	1.1	71	Female	0.65	2.22	5.07	33.53	No	No	Yes	No	1958.3	6.6
117	Case	CC	2015-12-30	2015-12-30	2.2	71	Male	1.60	3.09	4.89	23.18	Yes	Yes	Yes	No	1534.6	2.7
118	Case	CC	2013-11-30	2013-11-30	0.5	61	Male	8.24	2.74	17.55	22.91	No	No	Missing	Missing	1165.1	4.9
119	Case	CC	2013-03-27	2013-03-27	4.2	61	Male	3.35	4.50	16.98	Missing	No	No	Yes	No	236.6	11.5
120	Case	CC	2016-09-04	2016-09-04	2.6	70	Female	2.14	5.16	24.78	26.97	No	No	Yes	No	2811.2	7.1
121	Case	CC	2015-08-04	2015-08-04	6.8	74	Male	1.09	2.22	7.23	24.99	No	No	Yes	No	1296.3	4.9
122	Case	CC	2011-05-07	2011-05-07	2.5	77	Male	1.28	0.01	5.26	23.22	Yes	Yes	Yes	No	1557.0	10.5
123	Case	CC	2013-05-30	2013-05-30	4.4	72	Male	2.28	0.22	21.66	24.54	Yes	No	Yes	No	1721.7	15.1
124	Case	CC	2014-06-17	2014-06-17	0.3	68	Male	1.40	2.24	0.00	19.72	No	No	Yes	Yes	1705.2	10.0
125	Case	CC	2015-10-17	2015-10-17	1.9	69	Female	1.05	2.35	2.32	20.70	No	No	Yes	Yes	1108.4	4.6
126	Case	CC	2013-08-12	2013-08-12	0.3	78	Male	1.10	2.26	8.14	Missing	No	No	Missing	Missing	3571.2	12.3
127	Case	CC	2013-02-01	2013-02-01	4.3	73	Male	5.43	12.30	15.83	29.32	Yes	No	Yes	No	1367.1	5.7
128	Case	CC	2016-08-12	2016-08-12	7.8	60	Female	1.86	6.00	2.49	27.16	No	No	Yes	No	1506.0	16.7
129	Case	CC	2015-12-02	2015-12-02	2.1	63	Male	1.57	3.02	6.49	29.40	Yes	No	Yes	No	435.8	7.7
130	Case	CC	2014-03-18	2014-03-18	5.4	63	Male	3.66	4.60	4.62	17.07	Yes	No	Yes	No	1588.1	4.4
131	Case	CC	2015-04-20	2015-04-20	1.7	69	Male	0.87	8.80	6.99	31.60	Yes	Yes	Missing	Missing	4679.7	7.6
132	Case	CC	2012-12-04	2012-12-04	4.0	73	Male	3.41	14.60	18.46	26.56	No	No	Yes	No	1189.3	7.0

133	Case	CC	2013-08-10	2013-08-10	4.7	73	Female	3.40	8.20	2.00	31.47	No	No	Yes	No	311.7	3.6
134	Case	CC	2016-05-19	2016-05-19	2.5	66	Male	0.96	4.12	11.87	35.29	Yes	Yes	Yes	No	1415.1	11.1
135	Case	CC	2015-12-08	2015-12-08	2.1	68	Female	0.81	3.45	15.88	26.93	Yes	No	Missing	Missing	1808.6	7.8
136	Case	CC	2010-04-08	2010-04-08	1.3	72	Female	2.78	0.25	5.24	19.63	No	No	Yes	No	1105.8	6.0
137	Case	CC	2013-10-25	2013-10-25	0.4	77	Female	0.60	1.24	2.13	21.79	No	No	Missing	Missing	1610.2	3.5
138	Case	CC	2013-07-07	2013-07-07	4.8	70	Male	1.80	1.06	14.19	27.73	Yes	Yes	Yes	No	211.6	4.9
139	Case	CC	2015-03-23	2015-03-23	1.5	60	Female	1.12	4.31	4.13	29.09	No	No	Missing	Missing	3861.1	14.5
140	Case	CC	2013-11-26	2013-11-26	4.8	66	Female	0.61	16.30	22.96	21.70	No	No	No	No	634.7	10.8
141	Case	CC	2013-07-11	2013-07-11	0.5	68	Female	2.40	3.69	21.39	23.08	No	No	Yes	No	1524.5	2.9
142	Case	CC	2009-08-13	2009-08-13	0.8	60	Female	0.49	1.43	8.58	26.23	No	No	Yes	No	3738.3	7.7
143	Case	CC	2015-08-23	2015-08-23	1.5	73	Male	1.89	6.97	5.23	22.96	Yes	No	Yes	No	1218.7	6.7
144	Case	CC	2013-07-31	2013-07-31	0.6	71	Male	4.00	4.21	0.00	22.61	Yes	No	Yes	No	683.0	9.9
145	Case	CC	2016-02-23	2016-02-23	2.2	78	Female	4.02	2.63	49.02	27.77	No	No	Yes	No	378.0	13.7
146	Case	CC	2015-08-16	2015-08-16	6.8	76	Female	1.69	0.46	17.35	Missing	No	No	Yes	No	1169.9	8.3
147	Case	CC	2013-09-16	2013-09-16	0.9	62	Female	0.68	2.22	2.23	25.42	Yes	No	Yes	No	2629.0	4.0
148	Case	CC	2012-06-25	2012-06-25	3.7	68	Male	0.71	2.05	5.37	24.30	Yes	Yes	Yes	No	1525.1	5.2
149	Case	CC	2009-12-18	2009-12-18	1.1	73	Male	2.81	3.68	0.00	30.86	No	No	Yes	No	284.3	4.1
150	Case	CC	2011-08-01	2011-08-01	2.8	69	Female	1.44	4.54	0.00	23.61	No	No	Yes	No	1638.9	4.6
151	Case	CC	2015-03-16	2015-03-16	1.4	69	Male	2.79	2.85	7.45	20.37	Yes	Yes	Yes	No	1177.1	0.8
152	Case	CC	2014-01-16	2014-01-16	0.6	71	Male	3.60	3.11	4.92	23.63	Yes	Yes	Missing	Missing	1807.1	6.8
153	Case	CC	2016-03-24	2016-03-24	2.3	65	Male	7.17	2.24	17.78	28.73	Yes	Yes	Yes	No	3166.9	16.5
154	Case	CC	2014-12-06	2014-12-06	6.1	68	Female	0.92	6.40	11.48	23.26	No	No	Yes	No	1105.9	12.3
155	Case	CC	2009-12-09	2009-12-09	1.1	67	Male	0.70	2.64	2.18	25.46	Yes	Yes	Yes	No	3069.8	7.3
156	Case	RC	2013-11-10	2013-11-10	4.8	79	Male	1.19	68.60	12.47	22.77	Yes	Yes	Yes	No	897.6	3.7
157	Case	RC	2013-11-10	2013-11-10	0.8	68	Male	1.13	3.00	4.99	20.08	No	No	Yes	No	630.5	7.4
158	Case	RC	2010-01-09	2010-01-09	1.3	67	Female	0.07	0.01	1.54	23.06	No	No	Yes	No	1298.3	13.0
159	Case	RC	2016-10-08	2016-10-08	3.0	64	Male	0.97	3.64	2.72	22.95	No	Yes	Yes	No	2114.2	3.0
160	Case	RC	2014-07-02	2014-07-02	5.4	60	Male	3.20	11.30	14.78	22.51	Yes	Yes	Yes	No	300.4	7.2

161	Case	RC	2016-03-17	2016-03-17	2.1	67	Male	1.02	5.01	5.66	26.08	Yes	Yes	No	No	2032.8	5.5
162	Case	RC	2013-05-13	2013-05-13	0.2	65	Male	0.52	5.20	6.03	23.70	No	Yes	Yes	No	1608.8	10.5
163	Case	RC	2015-01-12	2015-01-12	1.6	68	Male	1.14	1.14	3.36	22.79	Yes	Yes	Missing	Missing	2139.8	6.2
164	Case	RC	2012-02-23	2012-02-23	3.1	65	Male	2.74	14.10	34.68	23.59	Yes	Yes	Yes	No	1449.2	4.2
165	Case	RC	2013-11-10	2013-11-10	0.8	62	Male	0.74	3.23	2.9	23.32	Yes	Yes	Yes	No	2086.9	6.5
166	Case	RC	2016-10-10	2016-10-10	7.8	69	Male	3.44	8.00	86.03	25.50	No	No	Yes	No	498.0	8.0
167	Case	RC	2013-11-10	2013-11-10	0.1	62	Male	1.38	2.06	5.11	25.83	No	No	Yes	No	1255.3	5.4
168	Case	RC	2010-12-18	2010-12-18	2.2	62	Male	5.72	9.70	8.86	19.38	Yes	No	Yes	No	1545.0	3.4
169	Case	RC	2012-09-24	2012-09-24	3.8	57	Female	3.10	12.90	26.51	20.36	No	No	Yes	No	1192.2	5.6
170	Case	RC	2009-06-15	2009-06-15	0.5	75	Male	2.77	4.80	16.56	21.28	Yes	No	Yes	No	6230.1	11.1
171	Case	RC	2010-05-26	2010-05-26	1.4	75	Male	3.35	4.20	7.09	24.54	No	No	Yes	No	314.7	5.2
172	Case	RC	2016-10-24	2016-10-24	2.9	72	Male	0.77	2.08	3.28	30.44	No	No	Yes	No	592.4	5.4
173	Case	RC	2013-11-10	2013-11-10	4.9	65	Male	6.13	6.20	55.94	20.92	No	No	Yes	No	377.1	6.7
174	Case	RC	2013-11-10	2013-11-10	5.0	85	Male	6.49	9.00	12.46	21.23	Yes	Yes	Yes	No	1798.7	5.8
175	Case	RC	2014-12-11	2014-12-11	6.0	59	Male	1.72	1.14	4.64	29.05	Yes	Yes	Yes	No	1195.9	7.7
176	Case	RC	2013-11-10	2013-11-10	4.9	70	Female	2.07	0.69	8.75	22.68	No	No	Yes	No	943.3	5.1
177	Case	RC	2013-11-10	2013-11-10	0.9	67	Female	1.62	5.62	0.00	22.48	No	No	Yes	No	1103.6	3.2
178	Case	RC	2014-01-20	2014-01-20	0.1	68	Male	2.05	2.92	4.93	28.09	Yes	No	Yes	No	2093.0	16.4
179	Case	RC	2014-08-24	2014-08-24	0.8	59	Male	2.58	7.18	0.00	25.65	Yes	Yes	Yes	No	848.7	12.7
180	Case	RC	2014-01-27	2014-01-27	0.3	70	Male	1.30	4.66	0.00	32.27	No	Yes	Yes	No	1635.7	2.3
181	Case	RC	2013-11-30	2013-11-30	0.1	63	Female	0.95	2.66	4.67	23.59	No	No	Missing	No	1160.3	15.8
182	Case	RC	2011-09-01	2011-09-01	2.8	60	Female	4.46	0.55	0.01	24.01	No	No	Yes	No	1757.8	3.9
183	Case	RC	2011-02-18	2011-02-18	2.3	64	Male	5.29	2.64	15.34	20.20	Yes	Yes	Yes	No	619.8	9.5
184	Case	RC	2013-10-16	2013-10-16	0.1	66	Male	5.23	5.51	17.99	23.14	Yes	Yes	Missing	Missing	4077.4	10.7
185	Case	RC	2016-08-22	2016-08-22	2.7	61	Female	1.38	2.52	5.29	29.05	No	No	Yes	No	1479.3	5.7
186	Case	RC	2013-11-10	2013-11-10	1.0	58	Female	1.55	2.89	0.00	21.76	No	No	Yes	No	1529.7	18.1
187	Case	RC	2010-09-30	2010-09-30	1.8	60	Female	2.47	7.10	4.75	25.39	No	No	Yes	No	1175.7	4.2
188	Case	RC	2010-11-24	2010-11-24	1.7	67	Male	2.35	12.40	13.60	24.57	No	No	Yes	No	1255.7	12.1

189	Case	RC	2014-01-03	2014-01-03	0.5	62	Male	8.39	1.54	7.05	27.40	Yes	No	Missing	Missing	4125.7	6.8
190	Case	RC	2016-08-15	2016-08-15	2.7	72	Male	1.30	3.70	6.63	26.67	No	No	Yes	No	1297.7	18.1
191	Case	RC	2013-11-10	2013-11-10	4.9	68	Male	3.75	8.00	12.90	17.51	Yes	Yes	No	No	390.3	5.7
192	Case	RC	2012-06-15	2012-06-15	3.4	57	Female	1.72	7.60	2.00	21.25	No	No	Yes	No	2216.9	6.8
193	Case	RC	2013-03-29	2013-03-29	4.4	65	Male	2.16	6.20	4.94	24.91	Yes	No	Yes	No	2017.2	6.4
194	Case	RC	2016-03-14	2016-03-14	2.8	62	Male	1.65	1.86	0.00	27.83	Yes	Yes	Missing	Missing	3897.7	9.0
195	Case	RC	2011-09-19	2011-09-19	2.9	75	Female	4.10	6.50	4.98	20.59	No	No	No	No	1515.4	2.0
196	Case	RC	2015-12-09	2015-12-09	2.4	53	Female	1.21	5.73	11.09	24.88	No	No	Missing	Missing	1566.1	2.6
197	Case	RC	2016-12-23	2016-12-23	3.2	62	Female	0.86	4.78	2.58	24.26	No	No	No	No	2095.6	7.3
198	Case	RC	2012-09-15	2012-09-15	3.9	70	Female	2.34	13.90	6.19	32.20	No	Yes	Yes	No	1513.5	5.1
199	Case	RC	2016-02-26	2016-02-26	2.4	68	Female	1.47	2.91	4.45	27.66	Yes	No	Yes	No	459.8	8.9
200	Case	RC	2013-11-10	2013-11-10	0.8	69	Female	0.96	3.64	4.9	24.78	No	No	Yes	No	1123.6	15.0
201	Case	RC	2013-11-10	2013-11-10	0.2	72	Female	1.83	8.21	4.52	24.35	No	No	Yes	No	2371.3	5.5
202	Case	RC	2013-11-10	2013-11-10	4.7	82	Female	695.70	12.60	310.65	23.14	No	No	No	No	480.5	12.6
203	Case	RC	2012-06-20	2012-06-20	3.4	68	Female	1.76	11.70	18.82	25.27	No	No	Yes	No	817.1	4.4
204	Case	RC	2012-04-23	2012-04-23	3.2	63	Female	2.09	11.30	3.88	26.39	No	No	Yes	No	264.6	4.7
205	Case	RC	2014-12-18	2014-12-18	1.1	75	Male	0.85	6.51	6.53	31.81	Yes	No	Yes	No	1610.2	13.8
206	Case	RC	2009-03-14	2009-03-14	0.4	74	Female	4.99	16.40	29.10	20.95	No	No	Yes	No	2686.0	5.3
207	Case	RC	2013-11-10	2013-11-10	0.9	64	Female	0.85	4.13	3.22	25.10	No	Yes	Yes	Yes	943.9	7.3
208	Case	RC	2013-11-10	2013-11-10	0.2	64	Female	2.86	4.74	0.00	21.86	No	No	Yes	No	1279.4	6.6
209	Case	RC	2016-10-12	2016-10-12	8.1	81	Female	1.57	3.89	28.63	21.52	No	No	No	No	187.2	13.1
210	Case	RC	2013-05-19	2013-05-19	4.4	75	Female	1.30	0.51	5.25	27.32	No	No	Yes	No	653.3	4.6
211	Case	RC	2012-04-06	2012-04-06	3.2	70	Female	2.11	0.01	22.82	20.11	No	No	Yes	No	2079.5	7.3
212	Case	RC	2016-02-23	2016-02-23	2.5	72	Female	1.09	2.27	0.00	22.27	No	No	Missing	Missing	2810.9	3.1
213	Case	RC	2016-08-10	2016-08-10	7.6	73	Female	2.12	10.10	13.56	21.34	No	No	Yes	No	312.7	8.9
214	Case	RC	2013-11-10	2013-11-10	4.8	77	Female	2.30	4.60	17.87	22.52	No	No	Yes	No	420.3	9.4
215	Case	RC	2010-11-22	2010-11-22	1.9	77	Female	4.03	4.80	10.87	26.02	No	No	Yes	No	117.9	7.5
216	Case	RC	2012-10-19	2012-10-19	4.0	67	Male	3.74	4.10	16.48	17.37	Yes	No	Yes	No	2164.6	5.5

217	Case	RC	2011-11-14	2011-11-14	2.9	60	Female	4.31	0.97	24.74	21.99	No	No	Yes	No	1223.2	5.8
218	Case	RC	2016-09-15	2016-09-15	2.8	62	Female	0.47	6.12	2.49	Missing	No	No	Yes	No	1208.0	7.2
219	Case	RC	2012-11-05	2012-11-05	4.0	56	Female	1.08	7.90	2.89	24.84	No	No	Yes	No	1760.6	10.0
220	Case	RC	2009-11-18	2009-11-18	0.8	81	Male	4.74	10.90	8.99	22.69	Yes	Yes	Yes	No	5151.0	5.2
221	Case	RC	2016-10-11	2016-10-11	2.7	67	Male	0.97	3.26	10.73	24.22	Yes	No	No	No	1840.3	10.9
222	Case	RC	2011-08-27	2011-08-27	2.8	64	Male	1.16	0.63	5.60	19.36	Yes	Yes	Yes	No	216.1	6.6
223	Case	RC	2012-12-17	2012-12-17	4.2	71	Male	1.12	2.90	0.00	19.15	Yes	No	Yes	No	1649.5	5.2
224	Case	RC	2013-07-12	2013-07-12	4.8	51	Male	1.50	8.46	10.41	18.25	Yes	Yes	No	No	679.4	5.2
225	Case	RC	2012-04-13	2012-04-13	3.5	57	Female	1.60	4.68	8.04	26.56	No	No	Yes	No	1123.8	7.2
226	Case	RC	2009-03-07	2009-03-07	0.3	77	Male	3.45	8.70	22.65	25.99	Yes	No	Yes	No	1426.1	9.8
227	Case	RC	2016-03-23	2016-03-23	7.4	71	Male	2.74	14.30	2.98	23.51	Yes	Yes	Yes	No	1892.4	3.0
228	Case	RC	2016-11-10	2016-11-10	7.6	76	Male	0.51	3.40	31.64	24.80	No	Yes	Yes	No	1309.5	8.4
229	Case	RC	2016-10-13	2016-10-13	2.6	78	Male	2.92	5.13	5.82	24.86	No	No	Yes	No	862.1	8.7
230	Case	RC	2013-11-26	2013-11-26	1.0	65	Male	1.66	8.23	14.07	19.49	No	No	Yes	No	1899.5	10.6
231	Case	RC	2016-08-29	2016-08-29	7.7	63	Male	2.73	13.90	5.00	25.95	Yes	No	Yes	No	339.0	4.0
232	Case	RC	2013-12-01	2013-12-01	0.1	65	Male	0.69	3.12	3.01	24.24	Yes	No	Missing	Missing	1140.6	3.4
233	Case	RC	2015-11-13	2015-11-13	2.2	90	Male	1.14	3.84	3.62	22.55	No	No	Missing	Missing	2050.0	6.9
234	Case	RC	2015-06-09	2015-06-09	1.8	76	Female	0.52	3.09	0.00	25.12	No	Yes	Missing	Missing	5277.2	11.7
235	Case	RC	2016-09-20	2016-09-20	3.0	62	Female	2.20	2.65	0.00	26.06	No	No	Missing	Missing	3857.3	15.2
236	Case	RC	2016-03-05	2016-03-05	2.5	61	Female	0.60	2.28	0.00	24.68	No	No	Missing	Missing	1689.0	11.4
237	Case	RC	2014-03-04	2014-03-04	5.0	58	Female	2.21	8.00	12.69	25.67	No	No	Yes	No	1148.6	4.1
238	Case	RC	2012-11-28	2012-11-28	3.9	78	Male	2.03	6.70	4.61	25.97	No	No	Yes	No	415.6	9.4
239	Case	RC	2016-04-13	2016-04-13	2.3	69	Male	1.20	3.22	5.96	27.43	Yes	No	Yes	No	3232.8	5.0
240	Case	RC	2012-08-14	2012-08-14	3.7	75	Female	1.92	0.38	17.01	27.41	No	No	Yes	No	2294.0	5.4
241	Case	RC	2013-04-13	2013-04-13	4.3	76	Female	1.61	7.20	7.05	21.27	No	No	Yes	No	2591.2	8.3
242	Case	RC	2011-10-06	2011-10-06	2.8	66	Female	1.81	11.70	2.00	27.16	No	No	Yes	No	1379.1	10.3
243	Case	RC	2012-07-07	2012-07-07	3.5	69	Female	2.74	7.10	6.85	26.30	No	No	Yes	No	1268.3	8.1
244	Case	RC	2011-09-27	2011-09-27	2.8	65	Male	1.23	14.20	12.95	24.75	No	No	No	No	322.0	4.5

245	Case	RC	2016-08-04	2016-08-04	2.4	73	Male	1.59	5.63	5.57	31.73	Yes	Yes	Yes	No	1141.8	10.2
246	Case	RC	2009-03-13	2009-03-13	0.0	63	Female	1.23	13.30	29.73	26.87	No	No	Yes	No	1402.3	9.7
247	Case	RC	2012-05-20	2012-05-20	3.5	59	Male	1.77	0.21	9.95	Missing	Yes	Yes	Yes	No	1718.9	5.6
248	Case	RC	2013-11-10	2013-11-10	0.9	58	Male	0.70	3.11	5.42	22.69	No	No	Yes	Missing	1471.2	8.9
249	Case	RC	2012-05-10	2012-05-10	3.5	61	Male	0.46	6.81	5.99	24.15	No	No	Yes	No	412.4	7.0
250	Case	RC	2013-11-10	2013-11-10	4.7	57	Female	1.50	731.50	11.42	19.31	No	No	Yes	No	1254.6	6.6
251	Case	RC	2013-12-02	2013-12-02	4.9	62	Male	2.31	11.80	9.64	28.40	Yes	Yes	Yes	No	1018.4	10.3
252	Case	RC	2012-05-22	2012-05-22	3.5	55	Female	0.41	7.13	17.25	23.31	No	No	Yes	No	1712.4	11.2
253	Case	RC	2013-07-04	2013-07-04	0.1	53	Female	0.69	2.61	0.00	24.84	No	No	Missing	Missing	2661.2	8.0
254	Case	RC	2013-07-29	2013-07-29	0.1	62	Male	1.24	2.12	8.88	20.03	Yes	Yes	Missing	Missing	3939.6	11.7
255	Case	RC	2013-05-29	2013-05-29	0.5	64	Male	4.06	2.13	35.86	28.97	Yes	Yes	Yes	No	2205.3	9.6
256	Case	RC	2013-11-10	2013-11-10	0.2	70	Male	0.67	2.56	2	29.76	No	Yes	Yes	No	3108.2	2.7
257	Case	RC	2013-08-05	2013-08-05	0.9	66	Male	2.14	4.55	18.17	23.14	No	No	Yes	No	1274.9	12.1
258	Case	RC	2013-11-10	2013-11-10	5.0	72	Female	3.10	4.70	9.10	20.55	No	No	Yes	No	430.1	1.5
259	Case	RC	2013-11-10	2013-11-10	5.1	62	Female	1.87	1.35	0.00	27.06	No	No	Yes	No	4265.6	4.5
260	Case	RC	2014-01-10	2014-01-10	0.2	56	Female	2.71	6.65	0.00	26.03	No	No	Yes	No	644.6	13.2
261	Case	RC	2013-11-18	2013-11-18	5.1	66	Male	0.31	2.09	8.68	23.24	No	No	Yes	No	2245.7	7.0
262	Case	RC	2015-05-04	2015-05-04	1.4	57	Male	2.32	4.17	32.04	30.76	Yes	No	Yes	No	565.7	7.6
263	Case	RC	2011-01-10	2011-01-10	2.3	63	Female	2.41	13.15	0.01	31.39	No	No	Yes	No	613.2	10.0
264	Case	RC	2016-03-21	2016-03-21	2.5	61	Male	1.17	2.76	7.29	25.92	No	No	Yes	No	2106.2	6.1
265	Case	RC	2014-02-02	2014-02-02	0.3	76	Male	9.55	11.21	4.3	22.95	Yes	No	No	No	1219.9	8.4
266	Case	RC	2013-06-16	2013-06-16	0.6	61	Male	0.54	3.18	10.98	28.80	Yes	Yes	Yes	No	3102.4	2.3
267	Case	RC	2012-08-10	2012-08-10	3.8	61	Male	0.37	2.44	10.65	22.58	Yes	No	Yes	No	2094.4	7.6
268	Case	RC	2010-03-10	2010-03-10	1.0	59	Male	1.43	12.20	6.56	21.37	Yes	No	Yes	No	1088.7	7.1
269	Case	RC	2012-04-16	2012-04-16	3.3	61	Male	2.47	10.20	5.41	22.56	No	No	Yes	No	2295.2	9.5
270	Case	RC	2016-03-30	2016-03-30	2.5	72	Male	1.73	5.73	11.94	22.99	Yes	No	Yes	No	1187.5	1.3
271	Case	CC	2016-02-09	2016-02-09	2.2	49	Male	2.35	3.45	0.00	23.39	Yes	No	Yes	No	2018.5	12.0
272	Case	RC	2015-01-06	2015-01-06	6.1	54	Female	0.16	0.18	11.58	25.15	No	No	Yes	No	1181.2	7.0

273	Case	RC	2014-06-16	2014-06-16	0.7	62	Male	2.79	2.06	0.00	23.34	No	Yes	Yes	No	1689.7	5.8
274	Case	RC	2014-11-03	2014-11-03	0.7	66	Female	0.65	2.63	3.85	31.11	No	Yes	Yes	No	673.4	4.8
275	Case	RC	2014-10-24	2014-10-24	1.0	60	Male	0.50	1.41	0.00	25.92	No	No	No	No	1316.8	8.4
276	Case	RC	2014-12-10	2014-12-10	1.0	61	Male	2.76	2.88	2.17	25.82	Yes	No	Yes	No	1766.2	10.0
277	Case	RC	2014-04-21	2014-04-21	0.9	67	Male	10.94	1.98	3.96	25.82	Yes	Yes	Missing	Missing	4261.1	2.1
278	Case	RC	2013-11-30	2013-11-30	0.4	57	Female	0.74	2.24	3.09	Missing	Missing	Missing	Missing	Missing	2477.3	8.0
279	Case	RC	2013-11-30	2013-11-30	0.5	56	Female	1.22	5.22	2.85	20.83	No	No	Missing	Missing	3908.7	3.4
280	Case	RC	2013-11-30	2013-11-30	0.5	81	Male	3.09	1.20	798.73	17.90	No	No	Missing	Missing	2929.7	3.3
281	Case	RC	2013-12-17	2013-12-17	0.1	73	Male	1.41	4.72	4.96	25.86	Yes	Yes	Yes	No	2229.4	11.6
282	Case	RC	2014-07-07	2014-07-07	0.8	62	Male	15.88	2.91	4.16	23.22	Yes	Yes	Yes	No	2014.0	9.1
283	Case	RC	2014-10-08	2014-10-08	1.1	79	Female	1.16	4.15	11.01	26.53	No	No	No	No	2366.6	10.5
284	Case	RC	2014-07-21	2014-07-21	5.8	71	Female	1.69	1.36	17.38	20.82	No	No	Yes	No	954.2	11.7
285	Case	RC	2015-03-23	2015-03-23	1.2	59	Male	0.81	1.91	2.46	Missing	Yes	Yes	No	No	2189.1	10.8
286	Case	RC	2014-03-03	2014-03-03	0.6	61	Female	1.78	4.15	0.00	26.49	No	No	Missing	Missing	1557.8	2.8
287	Case	RC	2014-12-04	2014-12-04	6.1	58	Female	1.51	3.91	24.00	30.15	No	No	Yes	No	486.1	10.7
288	Case	RC	2014-11-17	2014-11-17	1.2	62	Female	3.17	1.89	31.33	22.64	Yes	No	Yes	No	1164.7	7.3
289	Case	RC	2014-07-03	2014-07-03	0.7	71	Female	0.90	2.16	2.3	29.48	No	No	Yes	No	2296.8	10.0
290	Case	RC	2014-08-19	2014-08-19	0.8	63	Female	1.53	4.18	5.46	26.84	No	No	No	No	1225.5	9.5
291	Case	RC	2015-04-27	2015-04-27	1.8	64	Female	1.46	3.90	29.64	21.50	No	No	Missing	Missing	3606.7	11.7
292	Case	RC	2014-07-01	2014-07-01	5.3	62	Male	3.58	11.30	19.09	20.20	Yes	Yes	Yes	No	1421.4	7.9
293	Case	RC	2015-03-25	2015-03-25	1.7	64	Male	8.32	1.77	0.00	27.87	Yes	Yes	Missing	Missing	3796.2	12.0
294	Case	RC	2014-07-07	2014-07-07	0.9	60	Male	10.55	2.01	0.00	23.76	No	No	Missing	Missing	2819.8	5.3
295	Case	RC	2014-09-15	2014-09-15	0.8	68	Female	2.21	3.34	13.43	Missing	No	No	Yes	No	2731.2	10.5
296	Case	RC	2015-01-22	2015-01-22	1.2	59	Male	2.24	2.80	0.00	22.86	Yes	Yes	No	No	1432.1	9.4
297	Case	RC	2014-02-24	2014-02-24	0.4	66	Female	1.64	4.53	11.4	20.01	No	No	Yes	No	1780.2	6.2
298	Case	RC	2014-03-24	2014-03-24	0.4	63	Female	19.88	2.24	5.14	28.06	Yes	Yes	No	No	1545.0	5.0
299	Case	RC	2015-02-26	2015-02-26	1.4	69	Male	1.50	3.71	0.00	24.96	Yes	Yes	Yes	No	1261.3	9.5
300	Case	CC	2015-04-06	2015-04-06	6.5	73	Female	1.94	0.74	57.68	23.48	No	No	Yes	No	171.3	11.8

301	Case	RC	2014-03-27	2014-03-27	5.4	69	Male	1.63	0.49	12.03	22.77	No	Yes	Yes	No	1430.9	11.2
302	Case	RC	2015-08-19	2015-08-19	1.7	77	Male	0.77	4.10	75.35	31.60	No	No	No	No	1509.7	10.4
303	Case	RC	2015-08-04	2015-08-04	2.5	67	Male	1.22	6.59	4.42	29.21	No	No	Yes	No	1275.5	3.7
304	Case	CC	2014-09-18	2014-09-18	1.2	73	Male	1.21	5.30	4.05	21.01	Yes	Yes	Missing	No	2513.2	6.7
305	Case	CC	2018-11-17	2018-12-31	10.0	62	Female	3.99	0.54	29.87	Missing	No	No	Yes	No	2712.2	6.0
306	Case	CC	2018-10-31	2018-12-31	10.0	72	Female	2.82	0.33	15.13	29.00	No	Yes	Yes	No	1057.7	9.2
307	Case	CC	2017-03-07	2018-12-31	8.0	66	Male	2.73	12.40	11.16	22.15	Yes	No	Yes	No	1731.9	7.4
308	Control			2018-12-31		67	Male	3.35	8.10	14.16	21.40	Yes	Yes	Yes	No	427.9	5.9
309	Control			2018-12-31		72	Male	2.50	21.70	11.83	30.04	Yes	Yes	Yes	No	280.3	7.1
310	Control			2018-12-31		64	Female	2.60	8.70	26.44	22.21	No	No	Yes	No	445.9	4.6
311	Control			2018-12-31		68	Male	4.08	16.80	14.74	19.95	Yes	Yes	Yes	No	445.6	8.5
312	Control			2018-12-31		64	Male	3.69	9.80	2.00	19.24	Yes	No	Yes	No	357.9	8.1
313	Control			2018-12-31		75	Male	2.80	10.30	2.00	27.54	Yes	No	Yes	No	241.9	9.5
314	Control			2018-12-31		70	Male	2.28	17.40	2.70	25.41	Yes	Yes	No	No	335.6	12.6
315	Control			2018-12-31		58	Female	0.50	9.10	2.00	28.20	No	No	Yes	Yes	293.6	10.2
316	Control			2018-12-31		71	Male	2.90	1.39	9.64	25.76	Yes	No	Yes	No	1215.9	6.6
317	Control			2018-12-31		74	Male	2.13	2.56	0.74	26.38	No	No	Yes	No	1359.2	13.8
318	Control			2018-12-31		63	Female	0.63	2.34	21.98	21.37	No	No	Yes	No	2058.3	3.0
319	Control			2018-12-31		50	Female	0.29	0.01	5.08	21.23	No	No	No	No	1984.8	8.9
320	Control			2018-12-31		61	Male	0.59	0.02	2.65	26.07	Yes	Yes	Yes	No	1580.7	6.2
321	Control			2018-12-31		63	Male	0.20	0.01	6.68	22.72	Yes	Yes	Yes	No	1187.5	3.5
322	Control			2018-12-31		61	Male	0.58	1.31	13.55	21.41	Yes	Yes	Yes	No	1055.3	15.4
323	Control			2018-12-31		51	Female	0.87	1.49	13.46	23.84	No	No	Yes	No	1013.5	5.4
324	Control			2018-12-31		59	Female	0.17	0.01	7.53	26.10	No	No	Yes	No	1240.9	8.9
325	Control			2018-12-31		67	Male	0.96	10.56	29.94	24.88	No	No	No	No	1410.9	7.0
326	Control			2018-12-31		73	Male	0.44	0.01	1.52	25.84	No	No	Yes	Yes	1298.0	8.3
327	Control			2018-12-31		60	Male	1.21	1.16	12.09	25.30	Yes	Yes	No	No	316.5	14.0
328	Control			2018-12-31		68	Male	0.17	0.96	3.55	24.17	No	No	No	No	1235.8	7.3

329	Control	2018-12-31	77	Male	0.85	4.33	5.84	28.86	Yes	Yes	Yes	No	1452.3	4.7
330	Control	2018-12-31	72	Male	0.55	0.32	4.57	18.97	Yes	Yes	No	No	1100.9	14.1
331	Control	2018-12-31	70	Female	0.71	4.90	3.31	20.08	No	Yes	Yes	No	1857.2	7.3
332	Control	2018-12-31	74	Male	4.05	5.10	9.68	25.87	Yes	No	Yes	No	385.2	8.4
333	Control	2018-12-31	61	Male	1.39	14.40	12.80	25.60	Yes	No	Yes	No	909.2	6.4
334	Control	2018-12-31	68	Female	1.15	5.10	11.26	23.92	No	No	Yes	No	1573.2	11.3
335	Control	2018-12-31	67	Male	1.84	13.00	10.70	27.34	Yes	Yes	Yes	No	219.4	8.9
336	Control	2018-12-31	61	Male	2.05	8.70	12.82	27.25	Yes	Yes	Yes	No	680.1	1.5
337	Control	2018-12-31	78	Male	1.93	11.00	4.90	21.12	Yes	Yes	Yes	No	273.3	8.6
338	Control	2018-12-31	70	Male	3.23	18.40	5.00	27.45	Yes	No	Yes	No	260.5	2.2
339	Control	2018-12-31	82	Female	1.14	9.10	2.00	19.47	No	No	Yes	No	247.2	11.4
340	Control	2018-12-31	73	Male	1.97	15.30	22.65	19.37	Yes	No	Yes	No	1844.4	9.1
341	Control	2018-12-31	65	Male	2.22	5.50	2.00	19.69	Yes	Yes	Yes	No	202.9	2.2
342	Control	2018-12-31	61	Male	3.29	6.90	4.94	24.89	No	No	Yes	No	1487.3	11.7
343	Control	2018-12-31	62	Male	2.24	4.40	12.72	25.86	No	No	Yes	No	2191.7	11.5
344	Control	2018-12-31	57	Female	3.54	6.00	26.46	23.77	No	No	Yes	No	415.5	6.3
345	Control	2018-12-31	61	Male	1.05	4.80	10.17	24.91	Yes	No	Yes	No	642.6	6.8
346	Control	2018-12-31	60	Female	0.54	7.60	2.20	24.70	No	No	Yes	No	1327.3	7.6
347	Control	2018-12-31	61	Male	2.92	5.00	20.92	23.15	No	Yes	Yes	No	1136.8	4.0
348	Control	2018-12-31	60	Female	1.54	6.30	4.81	24.65	No	No	Yes	No	1336.0	8.0
349	Control	2018-12-31	58	Male	2.93	9.10	7.35	25.36	Yes	Yes	Yes	No	364.4	9.4
350	Control	2018-12-31	69	Male	2.93	10.50	12.46	19.25	Yes	Yes	Yes	No	1310.1	10.3
351	Control	2018-12-31	72	Male	1.90	5.96	7.79	18.87	Yes	Yes	Yes	No	1637.1	2.8
352	Control	2018-12-31	67	Male	2.25	8.10	16.18	25.22	Yes	Yes	Yes	No	1285.6	11.5
353	Control	2018-12-31	75	Female	1.48	14.10	6.16	21.22	No	No	Yes	No	1544.8	5.7
354	Control	2018-12-31	63	Male	0.99	1.87	7.80	25.83	No	No	Yes	No	951.3	7.4
355	Control	2018-12-31	71	Male	3.15	8.00	4.52	23.67	Yes	No	Yes	No	1885.5	11.2
356	Control	2018-12-31	60	Female	1.75	17.00	4.20	25.96	No	No	Yes	No	302.0	8.4

357	Control	2018-12-31	62	Male	2.58	9.30	29.69	28.15	No	No	Yes	No	895.6	17.4
358	Control	2018-12-31	63	Male	2.38	7.30	5.18	25.06	Yes	No	Yes	No	1524.2	6.1
359	Control	2018-12-31	63	Female	2.10	6.80	3.11	22.73	No	No	Yes	No	1141.7	12.2
360	Control	2018-12-31	81	Female	2.27	8.70	3.90	19.91	No	No	Yes	Yes	1137.4	8.6
361	Control	2018-12-31	81	Female	1.71	10.30	4.09	25.14	Yes	No	Yes	No	699.6	10.8
362	Control	2018-12-31	79	Female	1.06	6.40	2.00	Missing	No	No	Yes	No	1319.2	14.9
363	Control	2018-12-31	76	Female	3.67	12.70	2.54	27.87	No	No	Yes	No	673.5	7.0
364	Control	2018-12-31	77	Male	2.38	7.20	5.55	21.70	No	No	Yes	No	1675.3	4.5
365	Control	2018-12-31	72	Female	1.60	5.70	5.68	20.23	No	No	Yes	Yes	1795.6	10.3
366	Control	2018-12-31	72	Female	2.08	17.90	7.05	25.40	No	No	Yes	No	1213.8	1.6
367	Control	2018-12-31	70	Female	0.86	8.40	7.17	21.79	No	No	Yes	No	380.2	4.2
368	Control	2018-12-31	69	Male	1.65	6.70	16.72	25.16	Yes	No	Yes	No	394.0	5.7
369	Control	2018-12-31	59	Male	2.31	13.30	2.00	23.44	Yes	Yes	Yes	No	546.3	11.0
370	Control	2018-12-31	59	Male	4.71	8.30	8.07	22.96	Yes	Yes	Yes	No	300.2	6.3
371	Control	2018-12-31	60	Male	2.03	4.60	15.03	21.45	Yes	Yes	Yes	No	326.5	6.5
372	Control	2018-12-31	66	Female	1.32	11.70	9.74	26.11	No	Yes	Yes	No	347.1	6.9
373	Control	2018-12-31	58	Male	0.97	6.85	32.73	26.69	Yes	Yes	Yes	No	1480.6	10.5
374	Control	2018-12-31	58	Male	0.56	0.12	7.56	21.90	Yes	Yes	Yes	No	1282.0	5.9
375	Control	2018-12-31	63	Female	0.54	14.46	8.45	24.30	No	No	Yes	Yes	2748.0	7.5
376	Control	2018-12-31	72	Male	2.56	4.70	21.62	20.73	No	Yes	Yes	No	1698.9	4.9
377	Control	2018-12-31	76	Male	1.46	0.45	6.10	21.30	No	Yes	Yes	No	664.6	12.2
378	Control	2018-12-31	57	Female	0.94	5.72	20.49	19.95	No	No	Yes	No	304.4	6.9
379	Control	2018-12-31	67	Male	1.34	2.88	7.25	24.22	Yes	Yes	Yes	No	1643.0	5.1
380	Control	2018-12-31	69	Male	1.85	1.26	11.81	28.37	Yes	Yes	Yes	No	1661.2	6.9
381	Control	2018-12-31	69	Female	0.48	7.43	12.44	25.48	No	No	Yes	No	1576.7	6.6
382	Control	2018-12-31	63	Female	1.05	2.22	10.78	22.85	No	No	No	No	1178.3	2.4
383	Control	2018-12-31	62	Male	3.81	1.60	0.01	26.43	Yes	Yes	Yes	No	1104.9	7.4
384	Control	2018-12-31	60	Male	2.25	9.70	3.96	28.52	No	Yes	Yes	No	1174.9	9.1

385	Control	2018-12-31	58	Female	0.82	15.30	2.43	24.65	No	No	Yes	No	476.0	4.7
386	Control	2018-12-31	56	Female	0.86	15.30	3.89	24.62	No	No	Yes	No	1495.6	11.2
387	Control	2018-12-31	62	Male	2.33	4.80	11.15	24.84	Yes	Yes	Yes	No	1293.0	7.7
388	Control	2018-12-31	59	Female	1.94	5.60	7.11	21.78	No	No	Yes	No	588.5	3.6
389	Control	2018-12-31	61	Male	3.16	10.50	16.80	20.69	Yes	Yes	Yes	No	1500.0	8.4
390	Control	2018-12-31	62	Male	4.56	7.40	4.26	21.16	Yes	No	Yes	No	410.8	4.2
391	Control	2018-12-31	61	Female	0.92	4.55	0.00	32.66	Yes	Yes	Missing	Missing	1076.4	7.0
392	Control	2018-12-31	63	Male	4.00	0.44	0.08	23.42	Yes	No	Yes	No	1651.0	7.6
393	Control	2018-12-31	59	Male	0.54	0.26	4.31	30.86	Yes	No	No	No	1120.7	5.5
394	Control	2018-12-31	59	Male	1.47	0.01	1.86	25.06	Yes	Yes	Yes	No	1879.2	5.1
395	Control	2018-12-31	50	Female	0.85	12.19	12.72	24.46	No	No	Yes	No	1474.1	11.5
396	Control	2018-12-31	78	Male	1.56	0.71	12.38	26.49	No	No	No	No	1144.3	15.2
397	Control	2018-12-31	77	Male	3.77	0.73	31.61	21.19	Yes	No	Yes	No	1032.1	5.0
398	Control	2018-12-31	66	Female	0.44	1.11	15.59	22.21	No	No	Yes	No	430.3	3.1
399	Control	2018-12-31	78	Male	0.29	0.47	1.79	22.99	No	No	Yes	Yes	1652.4	4.3
400	Control	2018-12-31	75	Male	3.60	5.33	19.34	23.05	Yes	Yes	No	No	1233.8	11.2
401	Control	2018-12-31	75	Male	2.26	0.58	0.02	20.20	No	Yes	Yes	No	1475.5	12.5
402	Control	2018-12-31	70	Male	2.77	0.67	0.01	25.21	Yes	No	No	No	367.1	11.0
403	Control	2018-12-31	66	Male	1.32	3.57	7.65	25.56	No	Yes	Yes	No	1090.9	4.5
404	Control	2018-12-31	61	Male	1.72	2.39	12.16	26.70	Yes	No	Yes	No	855.5	4.7
405	Control	2018-12-31	65	Male	1.77	0.16	29.31	23.05	No	No	No	No	1755.8	6.6
406	Control	2018-12-31	64	Female	2.38	1.59	13.46	30.33	No	No	Yes	No	1695.6	2.4
407	Control	2018-12-31	73	Male	2.19	1.56	24.31	23.34	No	No	Yes	No	1127.2	13.1
408	Control	2018-12-31	67	Male	0.48	4.89	15.70	23.33	Yes	No	Yes	Missing	1036.6	11.1
409	Control	2018-12-31	63	Female	2.98	0.30	13.35	18.90	No	No	Yes	No	1164.2	4.5
410	Control	2018-12-31	66	Male	2.93	3.00	10.40	24.39	Yes	Yes	Yes	No	1445.1	9.3
411	Control	2018-12-31	65	Male	3.46	4.97	24.23	27.08	No	Yes	Yes	No	1381.6	5.9
412	Control	2018-12-31	64	Male	0.52	0.47	8.13	20.08	Yes	No	Yes	No	1274.1	7.9

413	Control	2018-12-31	74	Male	0.61	1.23	4.78	25.91	Yes	Yes	Yes	No	103.7	3.9
414	Control	2018-12-31	58	Female	3.52	1.07	13.39	25.21	No	No	Yes	No	1337.0	12.5
415	Control	2018-12-31	69	Male	3.11	1.64	20.43	22.86	Yes	Yes	Yes	No	1643.1	16.0
416	Control	2018-12-31	62	Male	1.70	7.89	13.01	22.27	No	No	Yes	No	1176.3	12.1
417	Control	2018-12-31	72	Male	0.62	4.60	12.38	27.04	No	Yes	Yes	No	1797.2	10.9
418	Control	2018-12-31	67	Male	1.00	2.43	9.40	26.67	Yes	No	Yes	No	1849.4	4.5
419	Control	2018-12-31	81	Male	1.57	3.14	26.08	24.84	Yes	Yes	Yes	No	1259.7	5.1
420	Control	2018-12-31	73	Male	0.00	6.80	0.00	24.11	Yes	No	No	No	1111.1	14.1
421	Control	2018-12-31	64	Male	0.55	5.73	18.53	25.39	Yes	No	Yes	No	1155.8	14.4
422	Control	2018-12-31	72	Female	0.22	0.36	4.82	19.61	No	No	Yes	No	335.9	5.4
423	Control	2018-12-31	59	Female	0.49	0.19	10.78	27.25	No	No	Yes	No	313.6	8.5
424	Control	2018-12-31	62	Male	2.39	4.01	10.59	23.94	Yes	Yes	Yes	No	320.1	4.2
425	Control	2018-12-31	62	Male	1.41	0.01	0.01	20.38	Yes	No	Yes	Yes	651.8	8.3
426	Control	2018-12-31	62	Female	0.87	7.90	31.71	25.77	No	No	No	No	504.5	7.0
427	Control	2018-12-31	63	Male	4.50	2.05	1.81	21.47	No	Yes	Yes	No	1507.3	7.0
428	Control	2018-12-31	68	Female	0.88	0.79	28.38	23.73	No	No	Yes	No	323.5	2.6
429	Control	2018-12-31	63	Male	2.36	2.01	21.26	17.90	Yes	Yes	No	Missing	474.5	3.5
430	Control	2018-12-31	63	Male	3.35	0.44	0.01	21.72	No	Yes	Yes	No	1520.6	2.8
431	Control	2018-12-31	66	Male	1.89	0.24	19.63	31.90	No	No	Yes	No	757.7	5.9
432	Control	2018-12-31	62	Male	0.91	2.56	4.02	23.72	Yes	Yes	Yes	No	1876.8	9.6
433	Control	2018-12-31	62	Male	2.50	1.90	21.16	20.98	Yes	Yes	Yes	No	357.1	0.5
434	Control	2018-12-31	62	Male	0.10	0.82	4.24	19.49	Yes	No	No	No	565.5	9.9
435	Control	2018-12-31	62	Male	4.12	1.15	10.26	21.22	Yes	No	Yes	No	1049.3	5.9
436	Control	2018-12-31	64	Male	1.30	2.08	20.85	25.35	Yes	Yes	Yes	No	401.1	15.9
437	Control	2018-12-31	66	Male	0.94	1.20	2.4	22.49	Yes	Yes	Missing	Missing	2903.8	6.5
438	Control	2018-12-31	78	Male	0.68	13.22	3.72	27.48	No	Yes	Missing	Missing	2528.0	6.2
439	Control	2018-12-31	67	Male	1.61	2.84	8.66	30.49	No	Yes	Missing	Missing	522.1	9.8
440	Control	2018-12-31	81	Female	1.37	12.70	11.54	19.02	No	No	Yes	Yes	1226.4	4.9

441	Control	2018-12-31	75	Male	1.19	13.00	3.95	25.14	No	No	Yes	No	1716.8	6.7
442	Control	2018-12-31	57	Male	4.30	11.70	10.81	20.88	Yes	No	Yes	No	185.0	12.7
443	Control	2018-12-31	61	Male	2.41	11.40	4.66	24.87	Yes	Yes	Yes	No	1822.9	12.9
444	Control	2018-12-31	68	Male	2.40	9.40	27.42	24.82	No	Yes	Yes	No	1105.0	5.5
445	Control	2018-12-31	77	Male	3.08	3.35	0.00	24.74	Yes	Yes	Missing	Missing	1661.7	6.8
446	Control	2018-12-31	65	Male	4.69	9.90	20.66	21.23	Yes	No	Yes	No	1485.6	6.5
447	Control	2018-12-31	64	Male	1.79	6.60	5.33	23.31	Yes	Yes	Yes	No	1217.4	15.6
448	Control	2018-12-31	67	Male	4.63	9.50	24.40	18.60	No	No	Yes	No	1188.6	5.0
449	Control	2018-12-31	67	Female	1.48	12.20	8.50	27.67	No	No	Yes	No	1258.7	4.5
450	Control	2018-12-31	68	Male	0.59	3.95	10.13	19.20	No	No	Yes	Yes	1182.1	13.8
451	Control	2018-12-31	62	Male	1.01	1.60	7.30	23.23	Yes	Yes	Yes	Missing	127.0	5.5
452	Control	2018-12-31	69	Male	0.96	4.79	9.26	22.68	Yes	Yes	No	No	1539.5	10.9
453	Control	2018-12-31	62	Female	0.66	0.23	20.97	32.79	No	No	Yes	No	1699.5	1.3
454	Control	2018-12-31	63	Male	0.58	0.52	11.21	28.40	No	No	Yes	No	1595.0	6.5
455	Control	2018-12-31	74	Male	0.72	4.05	7.25	25.65	No	No	Yes	No	1127.1	9.3
456	Control	2018-12-31	74	Female	0.16	0.88	2.15	21.48	No	No	Yes	No	1330.7	8.2
457	Control	2018-12-31	77	Female	2.20	2.00	6.90	23.73	No	No	Yes	No	1054.3	4.5
458	Control	2018-12-31	72	Female	0.79	1.76	34.20	25.88	No	Yes	No	Missing	1250.4	8.8
459	Control	2018-12-31	71	Male	3.40	1.40	0.01	24.68	Yes	No	Yes	No	1510.3	9.4
460	Control	2018-12-31	62	Male	3.42	3.23	3.28	23.51	Yes	No	Missing	Missing	1432.2	16.4
461	Control	2018-12-31	74	Male	2.28	0.52	27.76	22.27	Yes	No	Yes	No	126.6	11.3
462	Control	2018-12-31	58	Female	1.99	0.16	0.01	20.72	Yes	No	Yes	No	1711.8	9.5
463	Control	2018-12-31	58	Male	1.01	0.45	5.52	24.39	Yes	No	Yes	No	1308.2	5.1
464	Control	2018-12-31	60	Female	1.36	10.42	15.06	27.18	No	No	Yes	No	960.7	6.2
465	Control	2018-12-31	68	Male	0.40	0.60	4.40	26.35	No	No	Yes	No	1812.7	8.6
466	Control	2018-12-31	70	Male	2.20	0.10	5.20	16.73	Yes	No	Yes	No	1197.7	8.3
467	Control	2018-12-31	77	Male	1.56	9.43	24.87	23.71	Yes	No	Yes	No	1227.7	5.4
468	Control	2018-12-31	61	Female	0.26	0.43	3.65	21.97	No	No	Yes	No	934.7	10.2

469	Control	2018-12-31	64	Male	2.64	4.26	0.00	22.10	Yes	Yes	Missing	Missing	3144.9	6.8
470	Control	2018-12-31	69	Female	0.78	0.07	3.85	26.14	No	No	Yes	No	1041.3	5.1
471	Control	2018-12-31	60	Female	0.58	0.34	9.09	23.81	No	No	No	No	1339.9	5.6
472	Control	2018-12-31	62	Female	2.47	1.92	22.04	22.05	No	No	Yes	No	573.9	8.2
473	Control	2018-12-31	62	Male	2.03	7.20	4.41	26.67	No	Yes	Yes	No	488.8	6.9
474	Control	2018-12-31	64	Male	1.44	1.00	16.50	21.95	Yes	No	Yes	No	1143.9	3.1
475	Control	2018-12-31	63	Female	0.13	0.01	15.41	19.43	No	No	Yes	No	1481.7	7.0
476	Control	2018-12-31	66	Male	2.87	0.62	15.13	20.50	Yes	Yes	Yes	No	1532.5	3.8
477	Control	2018-12-31	64	Male	0.91	6.05	3.02	24.31	No	No	Missing	Missing	3353.8	4.6
478	Control	2018-12-31	69	Female	0.59	3.36	5.37	27.23	No	No	Yes	No	1149.0	6.1
479	Control	2018-12-31	68	Male	4.94	7.20	6.41	23.15	No	No	Yes	No	1304.0	6.1
480	Control	2018-12-31	68	Male	1.81	13.10	8.65	24.96	Yes	Yes	Yes	No	1146.9	12.7
481	Control	2018-12-31	66	Male	2.39	4.00	6.48	27.24	No	No	Yes	No	1369.5	8.9
482	Control	2018-12-31	71	Male	2.84	9.60	6.83	21.09	Yes	No	Yes	No	1127.7	8.4
483	Control	2018-12-31	64	Male	2.27	9.70	18.91	24.38	Yes	Yes	Yes	No	613.3	9.2
484	Control	2018-12-31	80	Male	3.16	5.50	8.72	23.94	Yes	Yes	Yes	No	1647.7	4.5
485	Control	2018-12-31	67	Female	2.03	8.20	7.75	21.36	No	No	Yes	No	1266.9	12.1
486	Control	2018-12-31	61	Female	2.09	14.20	11.58	23.15	No	No	Yes	No	977.2	5.5
487	Control	2018-12-31	66	Male	2.38	5.90	4.21	28.31	No	No	Yes	No	1112.3	4.8
488	Control	2018-12-31	63	Male	1.37	9.90	6.97	20.44	Yes	Yes	Yes	No	1583.2	15.1
489	Control	2018-12-31	59	Male	1.83	7.30	28.23	17.79	No	No	Yes	No	1720.4	11.0
490	Control	2018-12-31	78	Male	1.77	5.10	3.36	20.60	No	No	Yes	No	1154.0	2.9
491	Control	2018-12-31	71	Male	2.57	6.30	7.04	22.70	Yes	Yes	Yes	No	1561.6	1.9
492	Control	2018-12-31	72	Male	3.32	5.90	25.95	25.51	Yes	Yes	Yes	No	1028.5	6.2
493	Control	2018-12-31	64	Male	2.52	3.70	2.61	17.66	No	No	Yes	No	1478.0	7.0
494	Control	2018-12-31	62	Male	4.21	19.00	7.21	26.50	Yes	No	Yes	No	1469.0	11.0
495	Control	2018-12-31	63	Male	3.27	13.70	24.98	24.62	No	No	Yes	No	1279.8	2.4
496	Control	2018-12-31	57	Female	2.46	2.45	5.46	28.76	No	No	Yes	No	179.2	6.2

497	Control	2018-12-31	64	Male	0.77	4.57	2.9	24.24	No	Yes	Missing	No	799.2	7.6
498	Control	2018-12-31	64	Male	2.35	7.90	3.49	20.96	No	No	Yes	No	520.2	9.5
499	Control	2018-12-31	71	Male	4.33	14.90	34.09	20.66	Yes	Yes	Yes	No	1105.9	5.4
500	Control	2018-12-31	66	Male	3.59	7.70	19.79	24.03	No	No	Yes	No	2132.1	10.2
501	Control	2018-12-31	59	Female	2.89	15.60	2.00	16.45	No	No	Missing	No	1253.7	7.1
502	Control	2018-12-31	57	Female	2.10	8.20	17.63	26.25	No	No	Yes	No	352.7	3.9
503	Control	2018-12-31	57	Female	9.57	169.30	2.00	21.91	No	No	Yes	No	807.7	12.8
504	Control	2018-12-31	62	Male	1.63	3.79	4.21	22.86	No	Yes	Missing	Missing	859.7	9.8
505	Control	2018-12-31	65	Male	0.73	0.83	11.11	20.31	Yes	Yes	Yes	No	1584.8	18.2
506	Control	2018-12-31	71	Male	1.02	0.33	14.21	23.15	Yes	Yes	Yes	No	1172.9	7.2
507	Control	2018-12-31	55	Male	0.36	0.13	12.94	21.72	Yes	No	Yes	No	1756.6	9.2
508	Control	2018-12-31	68	Male	0.65	1.20	11.40	28.84	Yes	No	Yes	No	1181.2	7.0
509	Control	2018-12-31	70	Female	0.59	0.43	3.91	24.24	No	No	Yes	Yes	1296.2	7.2
510	Control	2018-12-31	76	Female	0.45	0.30	3.22	20.66	No	No	Yes	No	1293.0	8.3
511	Control	2018-12-31	59	Male	1.12	0.43	12.76	26.18	No	No	Yes	No	958.1	9.4
512	Control	2018-12-31	55	Female	4.29	8.68	34.79	23.31	No	No	Yes	No	1145.3	8.6
513	Control	2018-12-31	61	Female	2.74	0.20	15.35	22.52	No	No	Yes	No	1642.2	9.0
514	Control	2018-12-31	71	Female	1.61	5.60	20.27	20.24	No	No	Yes	No	306.4	4.7
515	Control	2018-12-31	76	Female	1.41	4.58	9	18.23	No	No	Yes	No	1032.8	3.2
516	Control	2018-12-31	85	Male	0.20	1.04	4.42	27.89	No	No	Yes	No	1238.5	6.4
517	Control	2018-12-31	61	Male	1.31	4.33	4.31	23.46	Yes	Yes	Yes	No	749.2	4.9
518	Control	2018-12-31	62	Male	1.46	17.70	11.17	25.68	Yes	No	Yes	No	1191.9	7.1
519	Control	2018-12-31	64	Female	1.19	5.80	16.22	22.66	No	No	Yes	No	1809.1	6.4
520	Control	2018-12-31	62	Male	2.13	10.30	3.34	20.81	Yes	No	Yes	No	1814.3	3.2
521	Control	2018-12-31	62	Female	0.58	5.10	2.73	19.31	No	No	Yes	No	635.5	9.5
522	Control	2018-12-31	59	Female	1.34	8.90	8.59	24.28	No	No	Yes	No	435.2	5.2
523	Control	2018-12-31	80	Male	2.60	16.30	13.63	25.15	Yes	Yes	Yes	No	227.7	10.3
524	Control	2018-12-31	61	Male	2.82	8.00	10.01	20.96	No	No	No	No	1364.9	6.4

525	Control	2018-12-31	61	Female	2.56	8.30	22.16	22.55	No	Yes	No	Yes	1541.2	12.0
526	Control	2018-12-31	65	Male	2.33	7.20	4.56	23.52	Yes	Yes	Yes	No	358.3	6.5
527	Control	2018-12-31	66	Male	1.06	2.89	7.7	27.61	No	No	Missing	Missing	1095.2	6.7
528	Control	2018-12-31	58	Female	1.62	9.50	3.87	22.88	No	No	Yes	No	685.5	12.3
529	Control	2018-12-31	75	Male	2.23	7.60	3.78	25.49	Yes	Yes	Yes	No	971.7	8.0
530	Control	2018-12-31	68	Female	1.59	9.50	4.03	29.76	No	No	Yes	No	1334.0	11.8
531	Control	2018-12-31	71	Male	2.06	7.10	10.10	22.98	Yes	Yes	Yes	No	1274.4	5.4
532	Control	2018-12-31	72	Female	1.08	13.50	8.63	20.24	No	No	Yes	No	359.6	2.8
533	Control	2018-12-31	76	Female	1.39	10.50	14.15	22.52	No	No	Yes	No	575.6	6.3
534	Control	2018-12-31	74	Male	1.72	13.10	3.25	19.90	Yes	Yes	Yes	No	587.4	1.6
535	Control	2018-12-31	69	Male	2.93	18.10	36.59	19.26	Yes	Yes	Yes	No	1344.2	3.7
536	Control	2018-12-31	78	Male	0.69	5.67	2.33	20.93	Yes	Yes	Missing	Missing	3245.6	8.2
537	Control	2018-12-31	65	Female	0.82	0.72	13.00	21.63	No	No	Yes	Yes	166.5	7.6
538	Control	2018-12-31	62	Male	2.26	0.01	7.04	19.24	Yes	No	No	No	214.9	16.1
539	Control	2018-12-31	59	Male	1.40	0.72	3.75	26.47	No	No	No	No	206.2	2.5
540	Control	2018-12-31	70	Female	1.09	6.70	8.25	27.34	No	No	No	Yes	1286.0	13.4
541	Control	2018-12-31	68	Male	1.01	3.52	33.93	22.33	Yes	Yes	Yes	No	1129.5	10.2
542	Control	2018-12-31	66	Male	3.42	1.14	7.12	21.94	Yes	Yes	Yes	No	1409.4	7.6
543	Control	2018-12-31	77	Female	0.57	1.01	17.28	19.06	No	No	Yes	No	339.1	3.0
544	Control	2018-12-31	62	Male	4.29	8.15	19.33	17.47	Yes	Yes	Yes	No	1055.3	12.5
545	Control	2018-12-31	69	Male	2.01	8.18	20.16	25.43	No	No	No	No	903.9	10.4
546	Control	2018-12-31	49	Male	0.23	0.01	3.23	24.36	Yes	No	No	No	367.8	4.2
547	Control	2018-12-31	62	Male	2.17	2.88	3.44	22.50	No	Yes	Missing	Missing	1108.9	6.2
548	Control	2018-12-31	69	Female	1.09	5.70	10.08	20.28	No	No	Yes	No	566.3	12.3
549	Control	2018-12-31	56	Female	0.70	2.73	5.38	24.52	No	No	Missing	Missing	246.3	1.7
550	Control	2018-12-31	63	Female	1.44	7.70	7.17	24.12	No	No	Yes	No	1727.1	11.4
551	Control	2018-12-31	73	Female	2.31	13.20	22.39	25.10	No	No	Yes	No	1498.4	8.3
552	Control	2018-12-31	70	Male	2.83	7.50	10.12	23.42	No	No	Yes	No	1275.4	5.1

553	Control	2018-12-31	73	Female	1.77	3.80	21.27	23.12	No	No	Yes	Yes	1649.5	8.4
554	Control	2018-12-31	74	Male	1.43	10.70	10.30	28.73	No	No	No	No	1214.8	7.5
555	Control	2018-12-31	74	Male	3.20	0.60	24.40	22.10	Yes	Yes	Yes	No	640.6	9.8
556	Control	2018-12-31	75	Female	1.69	1.69	24.66	24.98	No	No	Yes	No	1002.4	7.4
557	Control	2018-12-31	67	Male	1.58	2.73	7.72	29.76	Yes	Yes	Missing	Missing	1429.6	10.5
558	Control	2018-12-31	56	Female	2.08	7.80	11.56	28.68	No	No	Yes	Yes	1538.9	11.3
559	Control	2018-12-31	72	Female	1.37	8.60	2.56	25.27	No	No	Yes	No	1182.1	2.5
560	Control	2018-12-31	70	Female	1.45	6.30	10.36	20.95	No	Yes	Yes	No	1449.6	3.1
561	Control	2018-12-31	70	Female	1.44	5.90	6.12	26.23	No	No	Yes	No	1389.1	3.4
562	Control	2018-12-31	73	Female	3.04	17.80	8.83	24.34	No	No	Yes	No	1335.9	6.0
563	Control	2018-12-31	60	Female	1.64	5.40	5.62	21.23	No	No	Yes	No	200.6	6.4
564	Control	2018-12-31	56	Female	1.50	6.80	12.17	21.63	No	No	No	No	1002.5	10.1
565	Control	2018-12-31	68	Female	1.42	8.60	27.50	28.35	No	No	No	No	772.1	7.3
566	Control	2018-12-31	56	Female	0.92	3.60	4.98	23.23	No	No	Yes	No	1464.9	2.8
567	Control	2018-12-31	70	Female	1.00	17.30	8.04	21.75	No	No	Yes	No	1242.2	1.6
568	Control	2018-12-31	62	Female	1.21	2.45	2.57	22.60	No	No	Missing	Missing	1375.8	7.5
569	Control	2018-12-31	64	Female	1.86	2.24	16.51	25.92	Yes	Yes	Missing	Missing	2476.3	11.9
570	Control	2018-12-31	73	Female	0.10	0.89	7.41	19.38	No	No	Yes	No	1042.5	10.5
571	Control	2018-12-31	63	Female	0.33	1.71	3.10	26.00	No	Yes	Yes	No	1578.6	9.3
572	Control	2018-12-31	71	Female	2.50	5.76	12.44	30.26	No	No	Yes	No	1532.2	10.9
573	Control	2018-12-31	72	Female	1.54	1.24	7.03	25.54	No	No	Yes	No	301.0	8.6
574	Control	2018-12-31	70	Female	0.76	0.16	5.99	31.56	No	No	Yes	No	1321.1	11.4
575	Control	2018-12-31	53	Female	0.85	7.31	12.83	29.55	No	No	Missing	Missing	2693.2	5.3
576	Control	2018-12-31	81	Female	1.51	0.29	4.15	19.91	No	No	Yes	No	158.1	7.0
577	Control	2018-12-31	77	Female	0.94	0.31	0.94	23.53	No	No	Yes	No	1283.2	10.2
578	Control	2018-12-31	60	Female	1.05	3.30	5.19	30.30	No	No	Yes	Missing	2437.7	18.8
579	Control	2018-12-31	60	Female	0.77	4.73	2.14	26.00	No	No	Yes	Missing	175.6	13.1
580	Control	2018-12-31	55	Female	1.50	1.20	13.30	23.87	No	No	Yes	No	2034.1	5.8

581	Control	2018-12-31	66	Female	2.30	3.50	3.70	22.19	No	No	Yes	No	260.5	6.4
582	Control	2018-12-31	77	Female	0.80	2.70	31.90	21.64	No	No	Yes	No	642.5	3.7
583	Control	2018-12-31	72	Male	0.70	9.00	0.01	22.10	Yes	Yes	Yes	No	180.0	4.1
584	Control	2018-12-31	57	Female	0.81	2.86	0.00	20.78	No	No	Missing	Missing	1353.3	5.5
585	Control	2018-12-31	75	Female	1.93	18.80	8.85	21.18	No	No	No	No	1113.4	10.5
586	Control	2018-12-31	81	Female	1.13	3.40	4.53	22.99	No	No	Yes	No	1588.2	9.5
587	Control	2018-12-31	84	Male	2.83	7.60	17.56	30.09	Yes	No	No	No	1299.5	16.1
588	Control	2018-12-31	72	Female	2.50	9.60	26.17	18.82	No	No	Yes	No	1101.8	6.7
589	Control	2018-12-31	71	Female	1.70	4.50	19.73	23.31	No	No	No	No	1368.7	6.8
590	Control	2018-12-31	61	Female	1.85	12.70	6.66	20.89	No	No	Yes	No	487.1	6.6
591	Control	2018-12-31	59	Female	2.61	10.00	20.15	27.03	No	No	Yes	No	1733.0	6.5
592	Control	2018-12-31	59	Female	1.94	11.80	4.23	22.21	No	No	No	No	1347.6	9.4
593	Control	2018-12-31	54	Female	1.64	4.70	5.85	17.09	No	No	Yes	No	1638.3	5.4
594	Control	2018-12-31	56	Female	0.14	0.55	14.39	20.77	No	No	Yes	No	500.7	7.7
595	Control	2018-12-31	69	Female	0.91	0.26	6.92	19.56	No	No	Yes	No	1247.1	14.8
596	Control	2018-12-31	80	Female	1.84	0.31	20.70	22.97	No	No	Yes	No	1052.9	16.1
597	Control	2018-12-31	63	Female	0.56	1.01	1.70	25.39	No	No	Yes	No	258.0	4.2
598	Control	2018-12-31	77	Female	0.79	0.64	4.69	26.06	No	No	Yes	No	1450.9	4.1
599	Control	2018-12-31	75	Female	1.11	9.88	8.72	18.83	No	Yes	No	No	816.5	6.7
600	Control	2018-12-31	61	Female	1.68	17.90	31.35	20.83	No	No	Yes	No	363.0	13.8
601	Control	2018-12-31	56	Female	2.87	8.30	5.55	24.41	No	No	Yes	No	1754.1	3.9
602	Control	2018-12-31	57	Female	1.04	6.10	2.00	25.71	No	No	Yes	No	635.5	6.5
603	Control	2018-12-31	68	Female	2.22	11.70	12.31	19.54	Yes	No	Yes	No	1268.3	12.6
604	Control	2018-12-31	77	Female	2.23	4.40	12.81	28.31	Yes	No	Yes	No	1051.0	4.9
605	Control	2018-12-31	77	Female	2.61	7.50	11.10	21.33	No	No	Yes	No	1506.1	14.1
606	Control	2018-12-31	71	Female	1.90	3.10	4.72	21.36	No	No	Yes	Yes	1383.9	4.8
607	Control	2018-12-31	61	Female	2.41	10.10	3.49	21.12	No	No	Yes	No	1139.9	10.2
608	Control	2018-12-31	62	Female	1.94	9.60	4.00	32.99	No	No	Yes	No	221.6	5.9

609	Control	2018-12-31	64	Female	1.61	10.90	6.36	24.43	No	No	Yes	No	871.3	3.3
610	Control	2018-12-31	71	Female	0.43	3.85	19.21	20.88	No	Yes	Yes	No	501.5	2.7
611	Control	2018-12-31	79	Female	0.60	2.39	8.90	22.71	No	No	Yes	No	1276.8	7.2
612	Control	2018-12-31	54	Female	1.90	2.02	25.16	20.76	No	No	Yes	No	897.3	6.7
613	Control	2018-12-31	74	Female	1.36	0.25	7.51	20.15	No	No	Yes	No	598.3	16.7
614	Control	2018-12-31	56	Female	1.42	2.14	4.80	27.40	No	Yes	Yes	No	312.6	8.2
615	Control	2018-12-31	64	Male	1.42	1.80	28.36	17.10	Yes	No	No	No	422.8	7.7
616	Control	2018-12-31	68	Female	1.41	4.40	11.71	22.48	No	No	Yes	No	1178.8	7.1
617	Control	2018-12-31	73	Female	3.34	5.20	3.76	23.56	No	No	Yes	No	1545.3	11.0
618	Control	2018-12-31	73	Female	4.62	16.30	25.29	15.06	No	No	Yes	No	1023.2	11.0
619	Control	2018-12-31	66	Female	2.34	4.70	3.80	25.21	No	No	Yes	No	553.5	6.3
620	Control	2018-12-31	55	Female	1.13	2.50	0.00	22.35	No	No	Missing	Missing	1195.8	13.7
621	Control	2018-12-31	62	Male	1.86	15.50	5.32	24.64	Yes	Yes	Yes	No	1577.5	15.6
622	Control	2018-12-31	75	Female	0.24	4.21	13.69	28.76	No	No	No	No	1623.1	11.5
623	Control	2018-12-31	63	Female	0.36	0.09	18.07	19.44	No	No	Yes	No	253.7	7.5
624	Control	2018-12-31	58	Male	0.56	4.84	17.79	25.71	Yes	No	Yes	No	190.1	16.2
625	Control	2018-12-31	59	Male	1.31	1.24	10.75	29.39	No	No	Yes	No	1353.3	9.1
626	Control	2018-12-31	62	Female	1.34	0.45	12.03	26.30	No	No	Yes	No	1307.9	1.5
627	Control	2018-12-31	78	Female	0.34	4.85	10.77	25.56	No	No	Yes	No	315.7	11.9
628	Control	2018-12-31	60	Female	0.20	0.92	5.26	27.57	No	No	Yes	No	231.9	5.5
629	Control	2018-12-31	76	Female	0.78	5.45	29.71	23.46	No	No	Yes	No	136.2	9.1
630	Control	2018-12-31	60	Female	0.60	1.23	9.04	24.97	No	No	Yes	No	1768.6	6.0
631	Control	2018-12-31	53	Female	2.49	11.44	34.81	28.03	No	No	Yes	No	1570.3	12.4
632	Control	2018-12-31	56	Female	1.02	0.05	25.45	24.44	No	No	Yes	No	1127.7	10.9
633	Control	2018-12-31	54	Female	0.43	3.25	9.42	24.91	No	Yes	Missing	Missing	2109.5	7.1
634	Control	2018-12-31	62	Male	0.94	11.30	3.35	29.18	Yes	Yes	No	No	244.2	9.1
635	Control	2018-12-31	78	Female	1.45	11.30	7.34	22.43	No	No	No	No	203.4	14.4
636	Control	2018-12-31	67	Female	2.04	13.50	9.50	33.82	No	No	Yes	No	305.9	0.5

637	Control	2018-12-31	56	Female	2.24	15.70	20.91	25.52	No	Yes	No	No	838.9	3.7
638	Control	2018-12-31	84	Female	3.24	13.10	13.51	15.81	Yes	No	Yes	No	503.2	14.4
639	Control	2018-12-31	76	Female	0.84	10.40	5.21	24.68	No	No	Yes	No	451.2	6.2
640	Control	2018-12-31	61	Female	0.73	12.30	3.37	19.90	No	No	No	Yes	1465.8	7.2
641	Control	2018-12-31	60	Female	3.33	2.13	14.01	26.02	No	No	Yes	No	2172.2	4.1
642	Control	2018-12-31	54	Female	0.53	4.04	3.07	20.28	No	No	Missing	Missing	826.6	4.0
643	Control	2018-12-31	70	Female	1.65	12.80	10.88	27.39	No	No	Yes	No	1511.8	6.7
644	Control	2018-12-31	65	Female	2.28	1.86	5.45	Missing	Yes	Yes	Yes	No	1551.0	10.7
645	Control	2018-12-31	62	Female	0.39	1.59	24.55	22.87	No	No	Yes	No	1252.1	6.0
646	Control	2018-12-31	81	Male	3.58	7.10	23.73	26.62	No	No	Yes	No	1590.5	15.5
647	Control	2018-12-31	51	Female	1.11	5.40	4.38	24.32	No	No	Yes	No	1206.8	6.3
648	Control	2018-12-31	76	Female	3.76	5.67	21.25	23.42	No	No	Missing	Missing	2648.8	10.4
649	Control	2018-12-31	73	Female	2.72	11.00	7.21	24.65	No	No	Yes	No	1459.8	18.2
650	Control	2018-12-31	64	Female	2.91	5.90	6.06	24.65	No	No	Yes	No	1350.9	7.4
651	Control	2018-12-31	77	Female	1.99	12.20	3.22	23.92	No	No	Yes	No	1290.5	5.8
652	Control	2018-12-31	70	Female	0.73	5.20	5.96	23.04	No	No	Yes	No	624.8	7.2
653	Control	2018-12-31	76	Female	2.06	6.50	6.42	27.99	No	No	Yes	No	1561.9	10.5
654	Control	2018-12-31	71	Female	0.59	7.50	5.96	20.17	No	Yes	Yes	No	1195.4	14.6
655	Control	2018-12-31	58	Female	4.45	7.60	8.80	29.68	No	No	No	No	683.2	9.5
656	Control	2018-12-31	78	Male	1.76	10.50	6.43	27.79	Yes	Yes	Yes	No	1095.3	7.2
657	Control	2018-12-31	72	Male	1.89	9.90	12.83	27.43	Yes	No	No	No	985.2	14.4
658	Control	2018-12-31	69	Female	2.99	11.50	8.41	26.75	Yes	No	Yes	No	944.6	4.1
659	Control	2018-12-31	54	Female	1.04	3.81	2.94	25.33	No	No	Missing	Missing	2272.1	11.8
660	Control	2018-12-31	57	Female	0.30	2.11	1.15	19.95	No	No	No	No	130.5	9.5
661	Control	2018-12-31	59	Female	0.72	0.43	8.48	22.94	No	No	Yes	Missing	1373.7	8.1
662	Control	2018-12-31	65	Male	3.86	2.19	0.01	17.25	Yes	Yes	Yes	No	1277.7	5.8
663	Control	2018-12-31	71	Female	3.28	0.28	0.01	34.81	No	No	Yes	No	1193.2	9.5
664	Control	2018-12-31	73	Male	3.13	2.55	20.34	17.98	No	No	Yes	No	470.4	15.3

665	Control	2018-12-31	58	Female	1.74	12.94	6.33	28.73	No	No	Yes	No	1295.4	6.0
666	Control	2018-12-31	76	Female	0.42	2.91	3.35	25.99	No	No	Missing	Missing	1504.9	6.1
667	Control	2018-12-31	63	Female	1.13	4.10	0.00	19.57	No	No	Missing	Missing	1228.6	1.8
668	Control	2018-12-31	71	Female	4.07	13.20	17.08	22.21	No	No	Yes	No	1294.3	16.0
669	Control	2018-12-31	71	Female	4.12	6.80	9.52	21.78	No	No	Yes	No	803.3	9.3
670	Control	2018-12-31	59	Female	1.31	9.20	4.22	20.20	No	No	Yes	No	1620.5	4.9
671	Control	2018-12-31	67	Female	0.31	0.28	6.35	28.15	No	No	Yes	No	262.4	3.9
672	Control	2018-12-31	57	Female	0.75	1.90	21.58	28.15	No	No	Yes	No	513.8	13.7
673	Control	2018-12-31	60	Female	2.12	3.38	24.62	27.22	No	No	Yes	No	358.2	16.6
674	Control	2018-12-31	62	Female	0.86	1.50	13.52	25.15	No	No	No	No	467.2	5.8
675	Control	2018-12-31	77	Female	3.21	11.10	18.84	22.89	No	No	Yes	No	1666.6	4.1
676	Control	2018-12-31	69	Female	0.86	4.80	5.65	29.90	No	No	Yes	No	438.2	6.6
677	Control	2018-12-31	58	Female	1.86	8.10	16.79	25.45	No	No	Yes	No	1100.4	8.8
678	Control	2018-12-31	63	Female	1.53	10.30	8.07	24.80	No	No	Yes	No	1208.8	12.9
679	Control	2018-12-31	60	Female	1.06	15.60	7.06	20.72	No	No	Yes	No	1097.5	10.1
680	Control	2018-12-31	63	Female	1.54	14.70	5.22	24.30	No	No	Yes	No	321.9	6.6
681	Control	2018-12-31	61	Female	1.31	8.60	5.51	25.20	No	No	Yes	No	328.6	6.6
682	Control	2018-12-31	62	Female	1.80	9.30	3.56	24.42	No	No	Yes	No	595.2	8.4
683	Control	2018-12-31	57	Female	2.36	9.60	10.56	24.45	No	No	Yes	No	848.5	4.0
684	Control	2018-12-31	68	Female	3.19	8.70	17.12	25.07	Yes	No	Yes	No	876.0	4.2
685	Control	2018-12-31	72	Male	1.17	12.00	9.26	22.04	Yes	Yes	Yes	No	853.8	6.7
686	Control	2018-12-31	72	Male	2.10	6.80	4.06	25.78	No	No	Yes	No	941.3	9.0
687	Control	2018-12-31	72	Male	0.76	3.90	2.00	23.55	Yes	Yes	Yes	No	1310.8	12.2
688	Control	2018-12-31	65	Male	0.94	11.80	6.25	24.34	Yes	Yes	Yes	No	1902.7	14.4
689	Control	2018-12-31	62	Male	1.87	15.10	2.00	Missing	Yes	Yes	Yes	No	256.3	6.6
690	Control	2018-12-31	61	Male	1.62	8.50	6.22	23.73	Yes	Yes	Yes	No	270.7	8.7
691	Control	2018-12-31	63	Male	1.17	3.64	2.48	18.83	No	No	Missing	Missing	1450.7	6.6
692	Control	2018-12-31	63	Male	1.65	11.00	7.93	23.51	Yes	Yes	Yes	No	1231.3	9.1

693	Control	2018-12-31	62	Male	4.19	4.30	26.96	21.79	No	No	Yes	No	205.8	8.9
694	Control	2018-12-31	67	Male	4.45	7.20	9.79	21.63	Yes	No	Yes	No	954.3	1.1
695	Control	2018-12-31	53	Female	2.03	4.37	0.00	22.93	No	Yes	Missing	Missing	3282.2	3.5
696	Control	2018-12-31	65	Male	2.27	18.60	8.31	30.00	Yes	Yes	Yes	Yes	745.4	6.8
697	Control	2018-12-31	66	Female	0.25	0.40	12.41	19.43	No	No	Yes	No	1523.5	6.6
698	Control	2018-12-31	64	Male	3.92	6.85	1.21	25.01	Yes	Yes	Yes	No	1243.9	4.6
699	Control	2018-12-31	63	Male	1.89	0.56	8.77	22.31	Yes	Yes	Yes	No	1524.2	13.9
700	Control	2018-12-31	64	Male	1.45	2.11	2.78	23.51	Yes	Yes	Missing	Missing	3552.5	4.0
701	Control	2018-12-31	75	Male	2.18	2.94	27.64	23.74	Yes	Yes	No	No	1460.6	15.4
702	Control	2018-12-31	61	Male	2.42	1.38	18.45	26.04	No	No	Yes	No	1749.6	6.4
703	Control	2018-12-31	60	Male	1.06	4.58	0.00	22.76	Yes	Yes	Missing	Missing	1451.8	1.5
704	Control	2018-12-31	64	Female	0.84	3.35	0.00	22.66	No	No	Missing	Missing	3014.4	8.0
705	Control	2018-12-31	68	Male	1.15	3.50	16.64	25.51	Yes	Yes	Yes	No	1316.9	6.6
706	Control	2018-12-31	63	Male	1.63	0.04	3.92	24.11	No	Yes	No	No	819.0	4.0
707	Control	2018-12-31	61	Female	1.72	2.89	31.51	22.98	No	No	Yes	No	787.0	6.7
708	Control	2018-12-31	58	Female	1.07	1.97	21.16	22.07	No	No	Yes	No	1699.4	17.6
709	Control	2018-12-31	59	Male	1.62	1.33	15.51	24.01	No	No	Yes	No	1399.0	2.8
710	Control	2018-12-31	68	Male	1.10	0.40	22.70	24.62	No	No	Yes	No	1201.6	9.4
711	Control	2018-12-31	80	Male	1.15	1.14	19.88	24.98	Yes	No	Yes	No	1466.1	4.6
712	Control	2018-12-31	67	Male	0.69	0.00	3.12	29.05	No	No	No	No	165.5	1.9
713	Control	2018-12-31	59	Male	1.81	1.61	22.44	24.86	No	No	No	No	1120.9	6.8
714	Control	2018-12-31	67	Male	3.96	22.00	16.86	26.57	Yes	No	Yes	No	1557.4	6.9
715	Control	2018-12-31	60	Female	0.48	2.39	5.33	26.69	No	No	Missing	No	1332.0	6.7
716	Control	2018-12-31	62	Male	0.67	5.03	4.16	25.56	Yes	No	Missing	Missing	2451.8	10.2
717	Control	2018-12-31	64	Male	2.18	13.50	6.67	23.99	Yes	No	Yes	No	1566.9	6.7
718	Control	2018-12-31	69	Female	2.90	9.30	2.00	26.69	No	No	Yes	No	1287.1	10.4
719	Control	2018-12-31	68	Male	4.33	16.70	16.46	29.02	No	No	Yes	No	360.0	2.8
720	Control	2018-12-31	66	Male	4.30	7.80	2.00	30.48	No	No	Yes	No	1729.9	13.1

721	Control	2018-12-31	73	Male	3.18	10.50	9.89	27.00	No	No	Yes	No	1527.0	2.3
722	Control	2018-12-31	78	Male	2.87	5.00	6.51	23.47	Yes	Yes	Yes	No	1879.1	10.4
723	Control	2018-12-31	70	Male	1.31	8.00	6.85	31.28	Yes	No	Yes	No	47.7	7.8
724	Control	2018-12-31	75	Male	2.50	7.30	10.00	21.49	No	No	Yes	No	1960.5	8.2
725	Control	2018-12-31	59	Male	1.49	8.20	4.38	27.86	Yes	Yes	Yes	No	1662.3	7.4
726	Control	2018-12-31	68	Male	3.94	10.10	5.64	26.79	Yes	No	Yes	No	1300.0	3.4
727	Control	2018-12-31	62	Female	2.24	4.10	2.00	30.44	No	No	Yes	No	476.3	5.3
728	Control	2018-12-31	66	Male	1.59	6.40	7.31	20.94	Yes	Yes	No	No	304.6	10.8
729	Control	2018-12-31	69	Female	3.51	16.30	18.33	24.00	Yes	No	Yes	No	402.4	1.3
730	Control	2018-12-31	68	Male	3.37	9.30	2.00	22.55	Yes	Yes	Yes	No	532.6	7.4
731	Control	2018-12-31	66	Male	1.23	8.98	4.76	24.61	Yes	Yes	Yes	No	650.8	11.0
732	Control	2018-12-31	63	Female	2.21	10.20	9.57	23.73	No	No	Yes	No	394.7	6.2
733	Control	2018-12-31	59	Male	1.67	11.20	6.06	26.85	No	Yes	No	No	599.4	9.0
734	Control	2018-12-31	66	Female	1.33	11.13	34.62	22.81	No	No	No	No	1451.4	13.4
735	Control	2018-12-31	61	Male	2.66	5.40	5.97	23.77	Yes	No	Yes	No	336.7	4.6
736	Control	2018-12-31	65	Male	1.99	12.10	11.21	29.21	Yes	Yes	Yes	No	302.3	8.4
737	Control	2018-12-31	67	Male	1.56	2.79	29.53	18.36	Yes	Yes	Yes	No	1210.3	9.0
738	Control	2018-12-31	57	Male	1.26	2.31	5.89	25.69	No	No	Yes	No	1660.3	9.3
739	Control	2018-12-31	51	Male	2.62	10.10	2.72	19.25	Yes	Yes	No	No	379.0	8.3
740	Control	2018-12-31	77	Male	2.75	6.70	17.74	17.21	Yes	No	Yes	No	320.6	11.9
741	Control	2018-12-31	67	Male	4.28	0.01	12.93	24.28	Yes	No	Yes	No	810.0	6.4
742	Control	2018-12-31	71	Male	2.07	5.46	0.00	28.23	Yes	Yes	Missing	Missing	2754.8	7.8
743	Control	2018-12-31	61	Male	3.03	8.80	11.32	17.75	No	No	Yes	No	299.3	13.2
744	Control	2018-12-31	65	Male	4.92	9.70	4.23	18.11	Yes	Yes	Yes	No	289.2	6.1
745	Control	2018-12-31	62	Female	2.74	11.80	2.00	20.18	No	No	Yes	No	352.4	2.5
746	Control	2018-12-31	80	Male	2.54	6.70	2.00	23.60	Yes	Yes	Yes	No	730.1	12.7
747	Control	2018-12-31	76	Male	3.56	16.30	13.18	19.96	No	Yes	Yes	No	371.5	1.4
748	Control	2018-12-31	72	Male	4.48	5.80	3.90	22.99	Yes	Yes	Yes	No	1520.6	9.0

749	Control	2018-12-31	63	Male	1.06	1.96	12.16	24.28	No	Yes	Missing	No	535.1	2.3
750	Control	2018-12-31	77	Female	2.69	4.77	13.67	16.17	No	No	Missing	Missing	3037.9	6.3
751	Control	2018-12-31	77	Female	1.15	4.41	0.00	Missing	No	No	Missing	Missing	3665.7	9.7
752	Control	2018-12-31	68	Female	0.77	2.69	0.00	23.50	No	No	Missing	Missing	1337.0	7.4
753	Control	2018-12-31	71	Female	2.07	8.30	24.03	19.53	No	No	Yes	No	502.0	12.0
754	Control	2018-12-31	61	Female	0.88	2.04	5.75	19.22	No	No	Missing	Missing	1198.2	10.2
755	Control	2018-12-31	72	Female	0.48	2.26	2.61	19.47	No	No	Missing	Missing	2694.8	3.8
756	Control	2018-12-31	76	Female	1.88	5.70	21.89	29.41	No	No	Yes	Yes	1286.0	2.3
757	Control	2018-12-31	74	Male	1.97	8.10	8.42	21.26	No	Yes	Yes	No	1325.9	10.1
758	Control	2018-12-31	70	Male	4.56	6.90	11.73	22.22	Yes	No	Yes	No	1116.5	6.0
759	Control	2018-12-31	70	Male	1.97	5.70	8.56	23.04	Yes	Yes	Yes	No	876.6	10.0
760	Control	2018-12-31	72	Male	1.82	12.30	7.37	23.40	Yes	Yes	Yes	No	1231.2	4.8
761	Control	2018-12-31	70	Male	2.63	18.30	12.30	27.23	Yes	Yes	Yes	No	1485.5	6.8
762	Control	2018-12-31	82	Male	3.34	6.20	8.22	25.44	Yes	No	Yes	No	1302.4	11.9
763	Control	2018-12-31	69	Male	2.15	7.30	4.29	23.98	No	No	Yes	No	1185.2	10.0
764	Control	2018-12-31	66	Male	1.14	7.80	4.35	21.89	No	No	Yes	No	1197.2	11.3
765	Control	2018-12-31	53	Female	3.66	5.40	5.98	24.65	No	No	Yes	No	1510.6	9.9
766	Control	2018-12-31	63	Male	3.64	6.50	2.25	24.92	Yes	Yes	Yes	No	767.5	6.1
767	Control	2018-12-31	62	Male	1.55	6.72	6.07	19.77	Yes	No	Missing	Missing	3458.3	6.4
768	Control	2018-12-31	68	Female	1.49	12.10	4.72	25.60	No	No	Yes	Missing	1873.0	7.3
769	Control	2018-12-31	61	Male	2.04	12.50	6.10	25.14	No	No	Yes	No	655.3	0.6
770	Control	2018-12-31	57	Female	1.52	7.40	2.00	22.72	No	No	Yes	Missing	1722.4	4.9
771	Control	2018-12-31	55	Female	1.24	11.10	11.43	21.72	No	No	Yes	No	907.9	3.0
772	Control	2018-12-31	61	Male	3.04	18.60	2.00	14.61	No	No	Yes	No	1121.5	7.9
773	Control	2018-12-31	74	Male	0.97	10.30	2.00	29.78	Yes	No	Yes	No	451.1	7.0
774	Control	2018-12-31	68	Female	1.33	3.90	10.93	20.27	No	No	Missing	Missing	3245.6	2.3
775	Control	2018-12-31	65	Male	0.67	2.84	6.8	29.71	No	No	Missing	Missing	1775.0	2.2
776	Control	2018-12-31	80	Male	4.13	3.02	15.75	19.83	Yes	No	Yes	No	1121.8	5.0

777	Control	2018-12-31	71	Male	0.70	4.98	0.01	23.59	Yes	Yes	Yes	No	1209.2	12.5
778	Control	2018-12-31	75	Female	1.41	23.42	26.90	21.48	No	No	Yes	No	1741.2	11.9
779	Control	2018-12-31	63	Female	1.76	4.33	7.16	28.62	No	No	Missing	Missing	2600.7	3.6
780	Control	2018-12-31	70	Female	0.59	6.27	26.60	21.57	No	No	Yes	No	1706.8	3.5
781	Control	2018-12-31	70	Female	0.44	1.15	4.22	23.94	No	No	Yes	No	1448.2	17.9
782	Control	2018-12-31	56	Female	3.14	1.04	2.98	27.85	No	No	Yes	No	1394.2	4.4
783	Control	2018-12-31	60	Male	3.32	0.32	6.47	27.14	No	No	Yes	No	1455.9	6.7
784	Control	2018-12-31	71	Female	2.30	17.00	33.22	22.03	No	No	No	No	293.8	13.9
785	Control	2018-12-31	64	Male	2.46	22.40	17.16	21.45	Yes	No	No	No	352.6	7.0
786	Control	2018-12-31	72	Female	0.62	1.66	2.55	21.99	No	No	Missing	Missing	1420.8	2.9
787	Control	2018-12-31	70	Male	1.99	11.50	2.00	24.14	No	Yes	Yes	No	358.0	12.4
788	Control	2018-12-31	75	Male	4.15	19.50	30.10	27.68	Yes	No	Yes	No	421.6	2.7
789	Control	2018-12-31	66	Male	3.58	5.70	28.92	18.65	Yes	Yes	Yes	No	529.7	3.6
790	Control	2018-12-31	75	Female	1.59	6.60	16.14	23.67	No	No	Yes	No	319.8	1.4
791	Control	2018-12-31	55	Male	1.13	13.30	6.86	23.60	No	No	Yes	No	1827.9	13.0
792	Control	2018-12-31	67	Male	2.94	2.16	15.76	28.47	Yes	No	Yes	No	1589.7	3.9
793	Control	2018-12-31	73	Male	0.01	3.65	10.96	24.80	No	Yes	Yes	No	1147.7	9.3
794	Control	2018-12-31	73	Male	2.71	0.04	18.11	27.21	No	No	No	No	1193.8	12.8
795	Control	2018-12-31	79	Male	3.20	2.12	18.93	19.06	Yes	Yes	Yes	No	1500.6	6.0
796	Control	2018-12-31	75	Male	5.59	21.70	17.37	29.66	Yes	Yes	Yes	No	1185.6	7.2
797	Control	2018-12-31	73	Male	0.15	0.01	0.95	21.46	Yes	No	Yes	No	1594.2	9.3
798	Control	2018-12-31	53	Female	2.00	5.71	0.00	19.14	Yes	No	Missing	Missing	1364.5	5.5
799	Control	2018-12-31	61	Male	0.64	2.21	5.54	21.31	No	No	Missing	No	526.9	8.1
800	Control	2018-12-31	68	Female	0.67	0.01	3.01	21.57	No	No	Yes	No	778.4	3.8
801	Control	2018-12-31	71	Male	0.84	2.00	0.00	22.46	Yes	Yes	Missing	Missing	2937.5	14.8
802	Control	2018-12-31	56	Female	1.64	14.40	9.98	24.09	No	No	Yes	No	1308.7	9.8
803	Control	2018-12-31	62	Male	2.10	10.70	7.12	28.38	Yes	Yes	Yes	No	1211.4	6.7
804	Control	2018-12-31	65	Male	1.67	7.60	6.25	25.86	Yes	No	Yes	No	1697.3	9.2

805	Control	2018-12-31	65	Male	3.11	13.80	6.15	20.50	No	Yes	No	No	1058.7	2.4
806	Control	2018-12-31	71	Female	1.54	7.70	3.99	23.34	No	No	Yes	No	1174.5	5.7
807	Control	2018-12-31	64	Male	3.39	4.20	5.03	25.07	Yes	Yes	Yes	No	1439.6	6.1
808	Control	2018-12-31	66	Female	1.37	8.70	2.00	21.64	No	No	Yes	No	1345.9	5.0
809	Control	2018-12-31	60	Female	0.52	3.55	0.00	23.63	No	No	Missing	Missing	1312.5	9.7
810	Control	2018-12-31	62	Male	2.54	8.20	10.50	19.30	No	No	Yes	No	920.1	7.5
811	Control	2018-12-31	61	Male	2.10	6.90	15.20	29.06	No	No	Yes	No	1152.1	9.0
812	Control	2018-12-31	54	Female	1.92	14.00	3.05	19.43	No	No	Yes	No	1168.8	5.5
813	Control	2018-12-31	74	Male	3.87	7.80	25.86	22.44	Yes	No	Yes	No	1114.9	8.2
814	Control	2018-12-31	68	Female	1.65	6.70	5.90	20.13	No	No	Yes	No	1237.3	4.4
815	Control	2018-12-31	61	Female	0.69	11.00	6.34	27.68	No	No	Yes	Yes	1061.1	15.9
816	Control	2018-12-31	59	Female	2.35	4.00	19.69	19.43	No	No	Yes	No	1257.5	7.1
817	Control	2018-12-31	61	Male	1.97	7.20	4.25	24.75	Yes	Yes	No	No	1834.3	6.6
818	Control	2018-12-31	62	Male	2.21	4.90	5.39	18.78	Yes	Yes	Yes	No	735.1	5.5
819	Control	2018-12-31	65	Female	1.48	11.30	29.52	22.23	No	Yes	Yes	No	1726.4	14.0
820	Control	2018-12-31	65	Female	1.74	1.92	2.23	18.86	No	No	Yes	No	1201.3	2.8
821	Control	2018-12-31	65	Male	2.56	9.60	7.09	17.20	No	Yes	Yes	No	1113.6	0.7
822	Control	2018-12-31	68	Male	4.00	10.40	2.00	22.37	Yes	No	Yes	No	1442.5	5.2
823	Control	2018-12-31	65	Male	1.56	7.70	7.45	25.10	Yes	Yes	No	No	2094.1	9.1
824	Control	2018-12-31	75	Male	1.36	0.11	11.14	23.15	Yes	Yes	Yes	No	211.0	11.5
825	Control	2018-12-31	75	Male	0.19	0.66	2.51	26.93	No	Yes	Yes	No	1274.3	5.6
826	Control	2018-12-31	70	Male	1.86	0.48	7.14	24.16	Yes	Yes	Yes	Missing	1749.5	12.3
827	Control	2018-12-31	65	Male	0.50	1.64	5.66	25.32	Yes	No	Yes	No	1951.0	4.7
828	Control	2018-12-31	73	Male	0.97	1.74	0.00	24.09	No	Yes	Yes	No	1226.4	3.2
829	Control	2018-12-31	49	Male	0.92	0.32	9.92	25.59	Yes	Yes	Yes	No	1090.7	6.8
830	Control	2018-12-31	72	Male	0.39	4.85	4.83	23.98	No	Yes	Yes	No	1712.4	7.3
831	Control	2018-12-31	59	Male	2.10	3.54	7.22	20.83	No	Yes	Yes	No	1023.2	11.5
832	Control	2018-12-31	76	Male	1.80	0.12	4.47	20.83	No	No	Yes	No	1826.7	8.8

833	Control	2018-12-31	69	Male	2.10	4.61	32.74	22.49	No	No	No	No	1645.2	9.0
834	Control	2018-12-31	70	Male	1.79	3.54	8.60	Missing	Yes	No	Yes	No	1623.5	6.0
835	Control	2018-12-31	67	Male	0.73	5.59	11.40	21.85	No	No	Yes	No	1614.5	10.2
836	Control	2018-12-31	75	Male	3.59	1.29	5.19	23.94	No	Yes	Yes	No	1423.1	13.2
837	Control	2018-12-31	74	Male	1.53	2.74	3.44	22.84	Yes	Yes	Yes	No	1674.1	6.9
838	Control	2018-12-31	73	Female	2.36	2.51	21.22	21.43	No	No	Yes	No	1205.5	5.3
839	Control	2018-12-31	75	Male	1.63	0.39	13.83	27.82	Yes	Yes	Yes	No	852.0	10.3
840	Control	2018-12-31	66	Male	1.88	0.63	5.86	30.11	Yes	Yes	Yes	No	800.7	8.9
841	Control	2018-12-31	64	Male	1.91	1.00	25.58	22.50	Yes	No	No	No	1405.9	7.5
842	Control	2018-12-31	64	Male	1.38	0.94	0.00	23.95	Yes	Yes	Yes	No	1642.9	5.7
843	Control	2018-12-31	82	Male	3.17	1.97	19.81	30.30	No	No	Yes	No	1259.8	5.4
844	Control	2018-12-31	77	Male	1.80	0.53	4.67	26.08	Yes	No	Yes	Missing	1433.1	15.0
845	Control	2018-12-31	71	Male	0.93	4.19	7.77	25.86	No	No	Yes	No	1638.5	1.5
846	Control	2018-12-31	73	Male	1.95	6.23	65.58	22.49	No	No	Missing	Missing	1427.7	2.4
847	Control	2018-12-31	62	Male	3.30	12.63	17.45	19.96	No	No	Yes	No	1362.0	8.0
848	Control	2018-12-31	80	Female	1.11	16.50	17.48	26.79	No	No	Yes	No	1199.3	3.2
849	Control	2018-12-31	71	Female	2.56	12.70	8.24	25.32	No	No	Yes	Yes	1573.3	8.3
850	Control	2018-12-31	75	Male	1.73	7.40	6.48	22.16	Yes	No	Yes	No	1693.4	7.4
851	Control	2018-12-31	59	Female	1.97	24.63	11.39	25.15	No	No	No	Yes	1522.6	9.0
852	Control	2018-12-31	68	Male	0.99	7.60	7.01	29.32	No	No	Yes	Yes	1253.5	0.3
853	Control	2018-12-31	61	Male	2.23	10.50	6.58	26.91	Yes	Yes	Yes	No	1373.3	8.7
854	Control	2018-12-31	68	Female	3.16	3.04	33.95	25.00	No	No	Yes	No	1165.3	3.5
855	Control	2018-12-31	79	Female	2.00	9.70	24.52	24.39	No	No	Yes	No	1766.8	13.0
856	Control	2018-12-31	51	Male	1.78	13.10	5.50	28.41	Yes	No	Yes	No	1705.2	7.8
857	Control	2018-12-31	79	Male	1.00	10.20	20.43	23.53	No	No	Yes	No	456.5	3.7
858	Control	2018-12-31	65	Male	0.68	4.09	2.57	29.03	Yes	Yes	Yes	No	1152.6	6.0
859	Control	2018-12-31	63	Male	3.91	3.90	6.68	20.76	Yes	No	Yes	Missing	1643.6	3.7
860	Control	2018-12-31	61	Female	0.47	2.12	2.79	24.67	No	No	Missing	Missing	1323.8	10.4

861	Control	2018-12-31	64	Male	1.28	4.00	0.00	24.79	No	No	Missing	Missing	2845.2	5.8
862	Control	2018-12-31	69	Female	0.45	1.66	28.32	19.36	No	No	Yes	No	259.5	8.2
863	Control	2018-12-31	66	Male	2.44	13.51	29.84	21.26	No	No	Yes	No	733.5	9.6
864	Control	2018-12-31	67	Male	1.54	10.08	33.08	20.31	Yes	No	Yes	No	332.3	0.9
865	Control	2018-12-31	53	Female	0.33	6.50	0.00	23.15	No	No	Missing	Missing	608.2	10.0
866	Control	2018-12-31	65	Male	1.61	7.58	7.15	30.86	Yes	Yes	Missing	Missing	287.5	7.8
867	Control	2018-12-31	56	Female	0.87	2.44	0.00	25.88	No	No	Missing	Missing	357.7	6.7
868	Control	2018-12-31	62	Male	0.96	3.37	4.36	30.08	No	No	Missing	Missing	151.8	5.5
869	Control	2018-12-31	65	Female	1.19	2.68	7.87	21.29	No	No	No	No	1783.6	9.4
870	Control	2018-12-31	62	Female	1.75	0.76	9.54	21.63	No	No	Yes	No	1500.3	6.0
871	Control	2018-12-31	61	Male	0.99	4.15	13.64	27.18	Yes	No	Yes	No	1304.3	7.1
872	Control	2018-12-31	61	Male	0.22	1.98	6.14	28.96	No	No	Yes	No	1813.6	5.1
873	Control	2018-12-31	66	Female	0.64	5.36	2.46	23.73	No	No	Yes	No	1582.4	7.6
874	Control	2018-12-31	65	Male	1.74	13.96	10.11	25.40	Yes	Yes	Yes	No	1361.8	12.5
875	Control	2018-12-31	75	Male	1.87	6.60	15.77	21.67	No	Yes	Yes	No	1003.8	3.1
876	Control	2018-12-31	62	Male	1.20	2.93	2.21	25.86	No	No	Missing	Missing	1153.9	16.1
877	Control	2018-12-31	69	Female	1.79	7.57	17.11	22.48	No	Yes	No	Yes	1354.3	5.0
878	Control	2018-12-31	68	Male	3.73	3.61	7.19	29.39	No	No	Yes	No	1536.5	4.5
879	Control	2018-12-31	61	Female	0.69	2.51	4.7	24.67	No	No	Missing	Missing	1860.2	8.0
880	Control	2018-12-31	78	Female	3.95	20.00	5.36	26.67	No	No	Yes	No	1097.4	4.9
881	Control	2018-12-31	62	Male	0.47	1.21	3.8	24.86	Yes	No	Missing	Missing	1377.6	7.6
882	Control	2018-12-31	68	Female	0.87	5.72	9.74	22.19	No	No	Yes	No	1213.8	6.9
883	Control	2018-12-31	71	Male	1.59	3.26	5.72	15.43	Yes	Yes	No	No	828.2	6.3
884	Control	2018-12-31	69	Male	2.89	4.33	10.11	25.91	Yes	Yes	Missing	Missing	1349.2	8.1
885	Control	2018-12-31	71	Male	0.47	9.17	1.95	25.35	Yes	Yes	Yes	No	3093.5	4.2
886	Control	2018-12-31	62	Male	1.64	4.71	7.21	23.46	Yes	No	Missing	Missing	1091.2	6.7
887	Control	2018-12-31	69	Male	2.50	0.16	8.06	18.91	No	No	Yes	No	1946.8	8.5
888	Control	2018-12-31	65	Female	1.27	4.51	29.65	17.48	No	No	Missing	No	1676.5	5.7

889	Control	2018-12-31	61	Female	0.66	3.90	6.10	21.93	No	No	Yes	No	1640.4	8.2
890	Control	2018-12-31	78	Male	0.79	3.30	0.00	19.47	Yes	No	No	No	1151.4	10.1
891	Control	2018-12-31	75	Male	4.76	8.86	7.47	24.31	No	No	Yes	No	1930.8	8.7
892	Control	2018-12-31	81	Male	1.84	6.02	7.02	25.64	No	No	Missing	Missing	1332.7	11.8
893	Control	2018-12-31	72	Female	0.46	3.05	0.00	27.38	Yes	No	Missing	Missing	1462.6	9.8
894	Control	2018-12-31	81	Male	2.52	4.31	16.93	17.72	Yes	Yes	Missing	Missing	1430.0	15.5
895	Control	2018-12-31	68	Male	1.47	1.86	7.70	23.74	No	No	Missing	No	859.2	6.9
896	Control	2018-12-31	69	Male	1.43	2.65	52.85	23.83	Yes	Yes	Missing	Missing	256.7	8.0
897	Control	2018-12-31	59	Female	0.87	3.13	4.66	24.88	Yes	No	Yes	No	1864.3	5.2
898	Control	2018-12-31	65	Male	0.71	4.22	3	24.84	Yes	No	Missing	Missing	483.5	5.0
899	Control	2018-12-31	60	Male	0.80	4.95	6.45	24.61	Yes	Yes	Missing	Missing	972.4	2.4
900	Control	2018-12-31	71	Male	1.81	7.40	13.70	Missing	Yes	Yes	Yes	No	2651.5	4.9
901	Control	2018-12-31	73	Male	1.00	6.15	6.67	26.37	No	Yes	Yes	No	2342.6	8.8
902	Control	2018-12-31	65	Male	1.18	1.67	2.7	24.86	Yes	No	Missing	Missing	583.5	14.8
903	Control	2018-12-31	63	Male	0.86	1.99	6.47	20.20	Yes	Yes	Yes	No	1207.2	2.5
904	Control	2018-12-31	64	Male	1.03	4.39	2.22	22.65	No	Yes	Missing	Missing	330.7	8.9
905	Control	2018-12-31	58	Female	2.70	5.95	21.05	23.42	No	No	Yes	No	1353.8	1.0
906	Control	2018-12-31	82	Male	3.00	2.80	13.51	22.03	No	No	Yes	No	1872.2	3.2
907	Control	2018-12-31	77	Female	0.39	2.13	3.35	25.39	No	No	Yes	No	1698.9	9.9
908	Control	2018-12-31	61	Female	0.72	1.11	3.17	27.27	No	No	Yes	No	2207.2	5.2
909	Control	2018-12-31	71	Female	0.64	0.65	12.11	27.99	No	No	Yes	No	1217.5	5.8
910	Control	2018-12-31	69	Female	0.98	2.76	5.23	Missing	No	No	Yes	No	2043.8	14.4
911	Control	2018-12-31	77	Male	0.55	3.53	20.33	29.34	No	No	Yes	No	2094.1	6.7
912	Control	2018-12-31	71	Male	0.94	3.45	4.12	21.31	No	No	Missing	Missing	374.2	8.5
913	Control	2018-12-31	73	Female	3.74	13.90	4.41	23.97	No	No	Yes	No	1349.8	6.5
914	Control	2018-12-31	72	Female	2.17	7.00	12.22	26.48	Yes	No	No	No	1119.8	5.7
915	Control	2018-12-31	66	Male	1.74	162.80	8.13	25.00	Yes	Yes	Yes	No	362.7	5.0
916	Control	2018-12-31	66	Male	6.20	12.20	16.95	22.20	Yes	Yes	Yes	No	1447.2	8.1

917	Control	2018-12-31	65	Male	0.93	10.60	20.18	25.53	Yes	Yes	Yes	No	1167.1	13.5
918	Control	2018-12-31	63	Female	0.48	0.16	6.00	24.39	No	No	Yes	No	741.7	10.6
919	Control	2018-12-31	61	Female	1.71	8.40	13.31	22.37	Yes	No	Yes	No	828.5	16.3
920	Control	2018-12-31	71	Female	Missing	Missing	Missing	21.99	No	No	No	No	728.9	12.4
921	Control	2018-12-31	60	Female	1.13	4.50	8.08	26.25	No	No	No	No	1160.4	15.8

CC, colon cancer; RC, rectal cancer.

[1]Control were age (± 1 year, extended to 5 years) and sex frequency-matched to CRC cases at a ratio of 2:1.

[2]Individuals smoke at least one cigarette per day for more than six months were defined as current smokers (yes).

[3]Individuals drink at least once per week for more than six months were defined as current drinkers (yes).

[4]Regularly exercising for more than 20 min per day over the last six months was defined as physical activity (yes).

[5]Mean of triplicate measurements and the standard error of mean (SEM). Serum piR-54265 levels were determined from baseline blood of individuals when they were recruited in the cohort.

Table S6. Primers and probes used for droplet digital PCR in this study

RNA	Primer type	Sequence (5’ to 3’)
piR-54265	Stem-loop reverse transcription primer	TGACCGTCTGTATGGTTGTTCACGACTCCTTCACCCTATCCAACCATACAGACGGTCAGGTCAGGCT
	Forward primer	CCTGGAGGTGATGAACTGTCTG
	Reverse primer	TATGGTTGTTCACGACTCCTTCAC
	Probe	FAM-CCCTATCCAACCATACAGACGGTCAGG-BHQ1
cel-miR-39	Stem-loop reverse transcription primer	TGAACATCCTCTGGAGGCCAACTGCGTGAGCTTGTTACTCATTTTCTCAGCCTCCAGAGGATGTTCACAAGCTGAT
	Forward primer	CGGCTCACCGGGTGTAAATC
	Reverse primer	CAACTGCGTGAGCTTGTTACTC
	Probe	HEX-ATTTTCTCAGCCTCCAGAGGATGTTCA-BHQ1

Table S7. The piR-54265 levels of 30 serum specimens in two detection batches (copy/μL)

Batch	P5[a]	P25[a]	P50[a]	P75[a]	P95[a]	ICC[b]
1	364.51	1312.73	1876.54	3232.74	4309.18	0.997
2	338.95	1325.74	2013.73	3309.50	4448.75	

[a]P, percentiles.

[b]ICC, intraclass correlation coefficient.

Table S8. Comparison of serum piR-54265 with other blood markers for the efficacy of recognizing patients with CRC

	N[#]	piR-54265 (copy/μL)		Methylated *SEPTIN9* (+ or –)		CEA (ng/mL)		CA19-9 (Unit/mL)		CA125 (Unit/mL)	
		> 1500[a]	%	+[a]	%	> 5[a]	%	> 37[a]	%	> 35[a]	%
All patients	101	86	85.1	62	61.4	18	17.8	11	10.9	10	9.9
Stage I	16	15	93.8	11	68.8	4	25.0	2	12.5	1	6.3
Stage II	38	34	89.5	20	52.6	6	15.8	4	10.5	5	13.2
Stage III	36	26	72.2	24	66.7	7	19.4	4	11.1	4	11.1
Stage IV	11	11	100.0	7	63.6	1	9.1	1	9.1	0	0

[#]data was determined from blood of individuals at diagnosis prior to any treatments. [a]Cut-off value.

Table S9. The ability of prediagnostic serum piR-54265, CEA, CA125 and CA19-9 alone or combination for predicting future CRC

Biomarker	AUC (95% CI) for CRC diagnosis at			
	≤ 1 year (n = 79)	≤ 2 years (n = 132)	≤ 3 years (n = 198)	≤ 9 years (n = 307)
piR-54265	0.743 (0.685−0.802)*	0.711 (0.660−0.763)*	0.687 (0.643−0.731)*	0.642 (0.603−0.681)*
CEA	0.592 (0.520−0.664)	0.557 (0.500−0.614)	0.529 (0.481−0.577)	0.548 (0.508−0.588)
CA125	0.459 (0.403−0.515)	0.436 (0.390−0.482)	0.423 (0.382−0.464)	0.454 (0.416−0.492)
CA19-9	0.429 (0.354−0.504)	0.444 (0.385−0.502)	0.435 (0.386−0.483)	0.487 (0.446−0.528)
piR-54265 + CEA + CA125 + CA19-9	0.766 (0.708−0.824)*	0.741 (0.694−0.789)*	0.714 (0.672−0.756)*	0.654 (0.616−0.692)*

*$P < 0.05$ of Z-score test.

Table S10. Comparison of prediagnostic serum piR-54265 with other blood markers for the efficacy of recognizing future CRC patients

	N[#]	piR-54265 (copy/μl)		SEPTIN9 (+ or –)		CEA (ng/ml)		CA19-9 (Unit/ml)		CA125 (Unit/ml)	
		> 1500[a]	%	+	%	> 5[a]	%	> 37[a]	%	> 35[a]	%
Total cases	143	81	56.6	48	33.6	16	11.2	17	11.9	0	0
0 year–	63	36	57.1	23	36.5	11	17.5	3	4.8	0	0
1 year–	37	23	62.2	13	35.1	2	5.4	2	5.4	0	0
2 years–	37	18	48.7	11	29.7	3	8.1	1	2.7	0	0
3 years–	6	4	66.7	2	33.3	0	0	0	0	0	0

[#]Data was determined from baseline blood of individuals when they were recruited in the cohort.

[a]Cut-off value.
